# Biological relevance and methodological implications of unexpected hearing thresholds in a diving bird

**DOI:** 10.1038/s41598-024-82942-2

**Published:** 2024-12-23

**Authors:** Helen Rößler, Anne May, Michael Dähne

**Affiliations:** 1 Deutsches Meeresmuseum, Katharinenberg 14 – 20, 18439 Stralsund, Germany; 2 Faculty of Mathematics and Natural Sciences, University of Greifswald, Friedrich-Ludwig-Jahn-Straße 15 a, 17487 Greifswald, Germany; 3 Bundesamt Für Naturschutz, Insel Vilm, 18581 Putbus, Germany

**Keywords:** In-air hearing, Penguins, Behaviour, Psychophysics, Training, Threshold, Auditory system, Perception, Animal physiology

## Abstract

Many animals alternate between different media, such as air and water, thanks to specific adaptations. Among birds, penguins (*Sphenisciformes*) have the most extreme morphological, physiological, and behavioural adaptations to their amphibious lifestyle. Their auditory perception of sound, potentially matching different impedances in air and under water, is largely unknown particularly in terms of whether their underwater adaptations may have affected their in-air hearing capacity. In this context, we investigated the hearing ability of four captive Humboldt penguins (*Spheniscus humboldti*) in air using psychophysical hearing tests. The 50% hit rate was found to be below 76 dB rms re 20 µPa between 0.250 kHz and 10 kHz, with most sensitive hearing at 2 kHz (mean threshold of 15.3 dB rms re 20 µPa). The four penguins showed large inter-individual variation in sensitivity at a given frequency but within a common audiogram shape. Despite the variability, penguins detected 0.250 kHz at comparably low sound levels (mean = 36.8 dB rms re 20 µPa) after a rapid decline of sensitivity at 0.500 kHz (mean = 64.1 dB rms re 20 µPa). This finding was unexpected, and it is therefore difficult to interpret whether it is an artefact of the methods or a biologically relevant finding. An extensive discussion is presented and suggests that this finding may be biologically relevant but would need further investigation to reveal the underlying mechanisms.

**Supplementary Information:**

The online version contains supplementary material available at 10.1038/s41598-024-82942-2.

## Introduction

The environment in which animals live, strongly shapes the evolution of their hearing capabilities^1–5^. The life of many aquatic vertebrates takes place both in air and under water, which makes them amphibious in their hearing capabilities as well. The use of sound in both environments has been shown to lead to morphological changes of the ear in seals^6–10^, amphibians^11^, crocodilians^12–14^, and seabirds^15–17^.

In birds, the first mechanism underlying hearing corresponds to the sound entering the external acoustic meatus through a round opening, ordinarily hidden under the feathers at the side of the head and transmitted to the tympanic membrane. The sound wave is transferred from the inner surface of the tympanic membrane by the middle ear structures (the cartilaginous extra-columella, and the columella, a long single shaft bone) to the oval window where they enter the inner ear. In the inner ear, the basilar papillae of the pars cochlearis detects variation in the frequency of airborne pressure waves^4,18–20^.

The middle ear anatomy with its complicated lever structures, tissue tension and geometrical orientation has been shown to vary among species and depends on the mass, stiffness and other individual characteristics of the resonant system^21,22^. The middle ear system is not only crucial for matching the difference in sound impedance between the outer air and the fluid in the inner ear, it also defines the frequency range transmitted to the sensory epithelium^23^.

Recent comparative studies of middle ears in birds found that seabirds using underwater pursuit and deep diving for foraging, showed the largest changes in ear structure relative to terrestrial bird species. In these modified ears, the size of the areas transmitting the sound (i.e., the tympanic membrane and the columella footplate of the middle ear) was reduced. Diving birds using underwater pursuit also typically show a shorter extra stapedius, a reduced cranial air volume and connectivity, and several other modifications in line with reversals of low-to-high impedance-matching^17,24,25^. Penguins (Spheniscidae) are, however, the only bird species with an absent interaural connection^26–28^.

Anatomical ear adaptations to two mediums might come with compromises of perception in each medium, most likely depending on the species-specific needs^17^. However, such perceptual compromises have not been found in several seal species, like ringed seals (*Pusa hispida*)^29^, spotted seals (*Phoca largha*)^30^, harbour seals (*Phoca vitulina*)^31^, and northern elephant seals (*Mirounga angustirostris*)^10,31^, leading to the conclusion that semi-aquatic marine mammals can hear well both in air and under water but with a slightly more restricted frequency in air than under water^10,29^. Similar but opposite to seals, it was reported for crocodilians, who have an evolutionarily closer related ear anatomy to birds^4^, that the auditory sensitive frequency range was found to be slightly more narrow in water than in air^32,33^. The findings in these two very different animal classes suggest that the underlying mechanisms for aerial and aquatic hearing are somehow different and may influence each other but are still widely unknown.

Details of how water birds and seabirds perceive their acoustic surroundings in each medium has yet to be determined using baseline hearing research. The soft tissue and bone structures of the ear are only one complex factor underlying hearing capacity. Additional elements like tympanic pneumatic sinuses^34^, the orientation and innervation of the hair cell bundles^35^, the electrical^36,37^ and mechanical tuning^38^ of the hair cells and the central auditory system and neural processing^23^ are all related to hearing capabilities^39,40^.

The perception and use of any signal depends first on its detectability. Signal characteristics (frequency, amplitude, attenuation etc.) as well as the absolute auditory sensitivity of the signal’s receiver, both influence signal detection^41^. Biological connections with the social environment and attention may additionally influence perception of a signal such as individual identification calls or predatory calls. Penguin ecology is highly variable, potentially leading to acoustic behaviours adapted for the species’ specific environment closely associated with territoriality and habitat. Highly territorial species like brush-tailed penguins (*Pygoscelis*) live in dense colonies defending their rocky nests, while other species meet on large reproductive sites (e.g., the king penguin (*Aptenodytes patagonicus*)), and non-territorial species on cold vast ice sheets (e.g., the emperor penguin (*Aptenodytes forsteri*)). Depending on their habitat they therefore show large differences in visual and vocal displays as well as how they react towards potential threats^42,43^. The penguin species investigated in this study was the territorial Humboldt penguin (*Spheniscus humboldti*). This species lives on warm arid land areas of Chile and Peru, breeds in defended burrows in dense colonies, and forages mainly for fish in the cold Humboldt current^44^.

Behavioural or psychoacoustic measurements of hearing are the most direct and non-invasive assessments of an animal’s hearing capabilities^20^. In terrestrial birds (n = 16 species) the lowest absolute thresholds occur between 2 and 4 kHz at a median best sound pressure level of 8.1 dB re 20 µPa^45^. Between songbirds (Passeri) and non-songbirds tested, songbirds tend to have better high frequency sensitivity and poorer low frequency sensitivity than non-songbirds^45^. Regarding the auditory function and basic auditory capabilities of water birds and seabirds, only sparse details related to these measurements are known^46^. Psychophysical studies in air of a long-tailed duck (*Clangula hyemalis*)^47^, lesser scaups (*Aythya affinis*)^48^, mallard ducks (*Anas platyrhynchos*)^49^, and of a great cormorant (*Phalacrocorax carbo*)^50^ indicate that these birds on average hear best at 2 kHz - 3 kHz with absolute thresholds measured between 10 and 18 dB re 20 µPa sound pressure level, respectively. Psychoacoustic underwater measurements have been performed with long-tailed ducks (*Clangula hyemalis*), where thresholds could only be defined in ranges. At 0.5 kHz these thresholds were between 97 and 147 dB re 1 µPa, and at frequencies of 1.0, 2.0 and 2.86 kHz they were between 77 and 127 dB re 1 µPa^47^. The only other psychoacoustic underwater measurements have so far been performed with a great cormorant (*Phalacrocorax carbo*) at only 2 kHz and sensitivity was determined to be 64 dB re 1 μPa rms^51^. The fact that seabirds can hear underwater was supported by recent studies of Gentoo penguins (*Pygoscelis papua*) and common murres (*Uria aalge*) reacting aversively to frequency centred white noise above 100 dB re 1 µPa under water^52,53^.

Absolute hearing thresholds in highly specialised non-flight wing-propelled sea bird species such as penguins were so far only tested by cochlear potentials in African penguins (*Spheniscus demersus*) with most sensitive hearing ranges being found between 600 Hz and 4 kHz (mean of ~ 2.3 kHz) and within this range a minimum sound pressure of -6 dB re 20 µPa was required to produce a standard response of 0.1 µv at the round window^54^. These results agree with previous estimations by calculating the length of the cochlea^27,55^. How behavioural audiograms of penguins fit in line with those findings has not been determined, yet.

Here we investigated the absolute hearing threshold in air of four captive Humboldt penguins by using psychoacoustic measurements for the first time in any penguin species.

## Material and methods

### Animals and study site

Psychoacoustic trials in air were conducted with four Humboldt Penguins (*Spheniscus humboldti*) raised from three parental pairs in 2017 (two males: Lemmy and Jakob) and 2018 (two females: Frieda and Gustel), based at the Ozeaneum in Stralsund (Germany). The outdoor area of the penguin colony, with 13 burrows and a surrounding 1.2 m deep 120 m^3^ pool, is located on the roof top of the building and houses 10 Humboldt penguins. For winter, when warmer protection is needed an area underneath the outdoor rocky burrow area is directly accessible for the penguins through a closable entrance hole and for humans through an additional door. In this room a separated section was defined as training area for the sessions. All animals were in good health with no known history of ear injury or exposure to ototoxic medication.

### Training

The penguins were trained through standard operant conditioning and positive reinforcement techniques to indicate whether a tone was present or not by pointing to a round target when a tone was present (Go, signal trial), and remaining stationed at a cross when there was no tone played (No-Go, catch trial). If the penguin remained stationed when a tone was present it was recorded as a miss, and if the penguin moved towards the response target when no tone was present, it was recorded as a false alarm. The two males began training in May 2018 (age: eleven months) while the two females started to be trained in November 2018 (age: four months). The training sessions of the four penguins were conducted one to three times during weekdays. Penguins had access to fish only during training sessions until saturation with an average consumption of 200 g of halved sprat (*Sprattus sprattus*, Girgensohn, 1846) per session. In the absence of training activities (e.g., weekend), penguins were fed in the morning and/or at the end of the day. For more detail on the full training procedure, see (Rößler et al.^56^).

### Experimental set up and psychophysical trial procedure

**Fig. 1:**
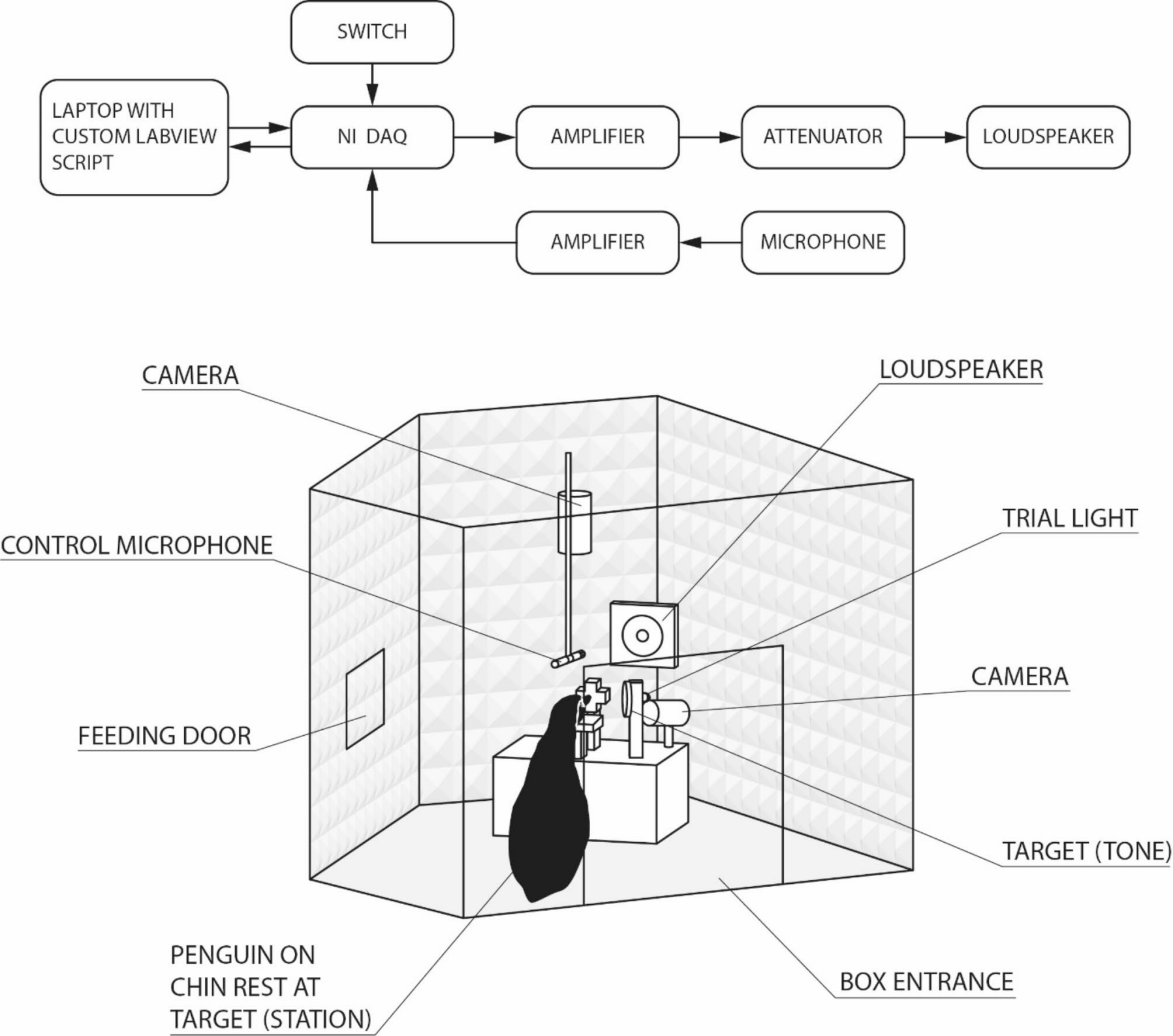
Top: diagram of equipment schematic for the signal production and receiving chains; Bottom: diagram of the behavioural audiogram set up during a hearing test session inside the anechoic box.

A custom-built anechoic pentagon shaped box (1.2 m high and 1.8 m at its widest) was assembled in the training area in April 2019. The inside of the box was covered with pyramidal acoustic foam on all sides and the top (aixFOAM SH003 MH, 70 mm thick, Schaumstoffe Helgers GmbH, Eschweiler, Germany), and convoluted foam on the bottom (Pursinus70, 70 mm thick, Flexolan GmbH, Ulm, Germany). This box had several openings to stepwise train the penguins to come inside the box and voluntarily partake in progressively closed, non-visual training (Fig. 1). Finally, the box was completely closed to have a double-blind testing set up. The trainers then only saw the penguins via video screen showing the penguin in the box from two different positions (above and front) as live feed from video cameras (Samsung, SEB-1005R) connected to a DVR recorder (Samsung, SDE-5001N; Fig. 1). Several positions were tested to optimise the position of the speaker, response target and penguin and was finalised with the speaker being positioned 75 cm away and at the upper front of the stationed penguin, the response target right of the stationed penguin and the reward window left of the stationed penguin (Fig. 1).

To minimise issues with surrounding noise sources in the room (e.g. water circulation pumps), acoustic foam was positioned on the walls of the whole training area and moveable acoustic dampening walls were also built and positioned within the room.

A customised LabVIEW (version 2017, National Instruments Corp., USA) script was written for automated, randomised, and double-blind psychophysical test sessions (supplementary material 1). This was run from a laptop computer connected with USB to a Multifunction I/O Device (USB-6366, National Instruments Corp., USA). The output was connected to an amplifier (NS-10G PRO Hi-Fi DSP 100W, Nobsound Douk Audio, Shenzhen, China) adjusting the input signal to avoid a fixed built-in squelch. An adjustable attenuator was used to prevent unwanted harmonics (ATN1270, etec, Denmark) and attached to a 13 cm diameter loudspeaker (SWR5004, 4 Ω, 80 W, LTC audio, Lotronic international, Belgium) inside the anechoic box (top schematic in Fig. 1).

The trials were started with a custom-made switch. A trial light inside the box in front of the penguin was activated and showed the start of a trial to the animal 300 ms before a possible tone (600 ms long) appeared, and the light turned off 1.1 s after the start of the trial (Fig. 1). Two LEDs (green and red) on the switch were used to indicate to the trainer what trial type (signal or non-signal) was presented. To obtain a measure of spontaneous responding, catch trials were inserted for 40% of the trials in one session, during which no test signal was presented, which results in a 60:40 signal to non-signal ratio. A response during a catch trial or during a waiting interval resulted in a station period adjusted to the specific penguin with the trial light being switched off. The length of the station period was manipulated to control the false alarm rate of the birds. If the bird responded especially clearly and correctly, he/she was rewarded with one whole fish instead of half a fish.

### Acoustic stimuli procedure

#### During training periods

The training tone was a manually activated 2 kHz sinusoidal soundwave played for 1–3 s, from a signal generator (HPG1, Velleman Instruments, Belgium) at approximately 55 dB re 20 µPa. The longer signal was to help establish stimulus control with the task.

#### During constant stimulus sessions to estimate approximate hearing

The first preliminary hearing test sessions with constant frequency and constant sound pressure level (SPL) with randomised catch trials (= c session type;^57,58^) started at the end of March 2020. A 600 ms long 2 kHz pure tone, with a 5% ramping to prevent acoustical smearing, was played at 59 dB re 20 µPa received level. Typically, the penguins participated in one or two audiometric sessions per day, five days per week.

**Fig. 2:**
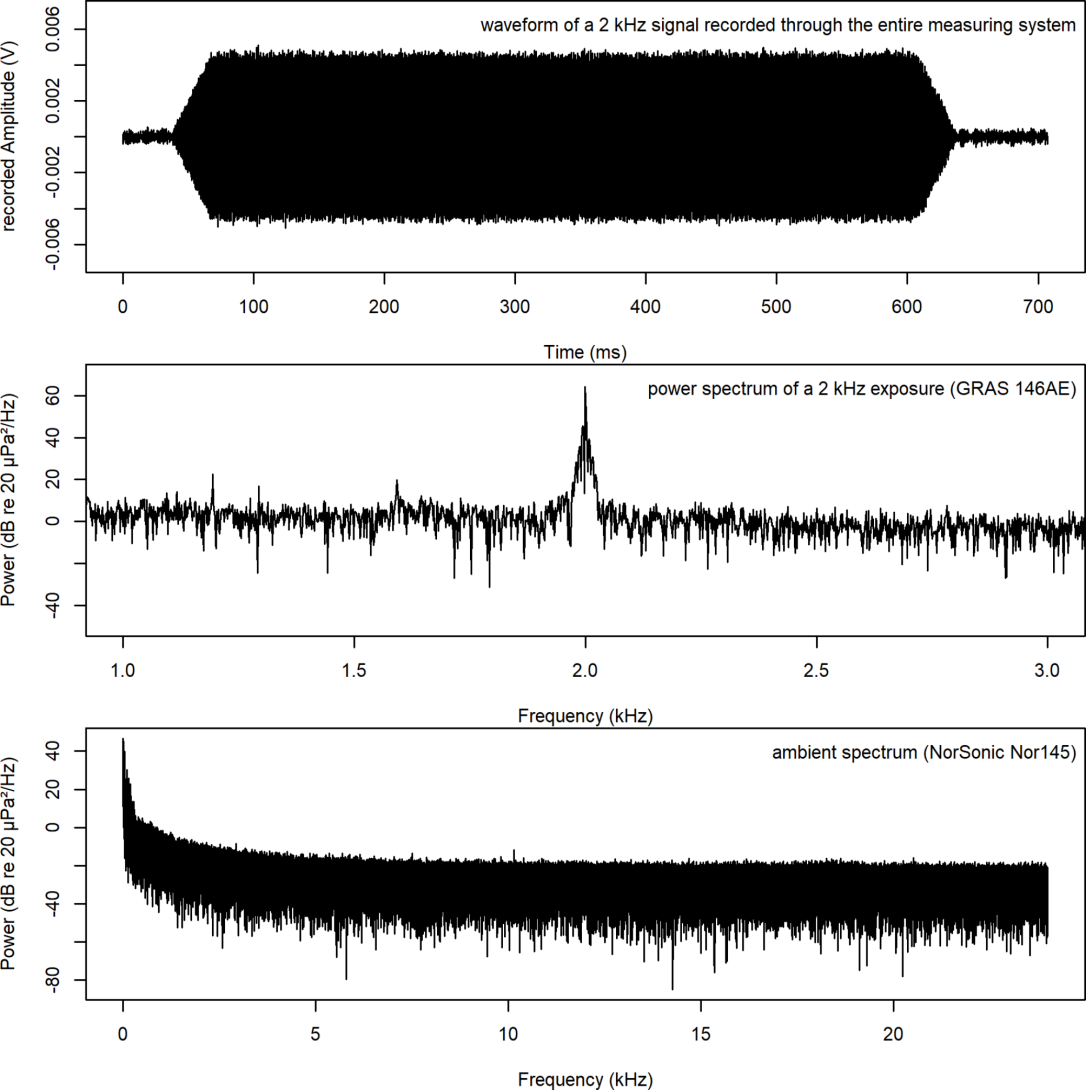
Sound stimulus recording at 2 kHz with background noise (sampling rate 48 kHz, 24 bits signal at -36 dB re 1 V output at the maximum tested level; top in time domain and middle in frequency domain (FFT size 4096). Bottom: Self-noise of Norsonic sound level instrument (sampling rate 48 kHz, 16 bits, FFT size 4096).

The test sessions gave the answer criterion (detection probability) for each penguin^59,60^. Reliability for each penguin was different with Jakob displaying the most stable response, Lemmy, Frieda and then Gustel with most changing responses between sessions (with an average of 21 trials per session and max. 80 trials per session) at the same sound pressure level. After one week the sound pressure level at 2 kHz was reduced in 6 dB steps until -36 dB (= 23 dB re 20 µPa). In the next step, frequency was changed to 250 Hz, 500 Hz, 1 kHz, 3 kHz, 4 kHz, 8 kHz, 10 kHz. At each frequency, different maximum sound pressure levels were used with 6 dB reduction steps until the penguins were close to the 50% chance level of detection rate^61^. Tests were only carried out when the recorded background and electronic noise was sufficiently low to detect the signal (Fig. 2). When the penguins did not hear the tone, it became difficult to make sure that they did not change their answer criterion to a more liberal one^59,62^. It therefore became necessary to come back to the training tone to “recalibrate” the penguins after every new period of trials (approximately 1 week or less). Every day, every trial result was monitored and if necessary, the number of trials, frequency and amplitude or reinforcement was appropriately changed. With this constant method (c session type = one frequency with one SPL per session), a first estimation of the penguins’ approximate hearing threshold was gathered.

#### During method of constant stimuli to determine audiogram

In February 2021, the literature-supported method of constant stimuli (sc session type = one frequency with randomised^61^ changing sound pressure levels in one session^59^) was used for all further test sessions for the audiogram determination (with an average of 15 trials per session and max. 56 trials per session) until December 2021. We tested, with the method of constant stimuli (sc), at 125 Hz, 250 Hz, 500 Hz, 1 kHz, 2 kHz, 3 kHz, 4 kHz, 8 kHz, and 10 kHz, using predefined sound pressure levels of -12 dB, -6 dB, 0 dB (previously estimated expected threshold from the c session type), 6 dB and 12 dB. The first three trials in a session were always with the loudest stimulus well above threshold. This way, we made sure that the penguin was made aware of the stimulus type used. Only sessions with a high motivation and concentration level (2.5 and 3; category 2.5: penguin paying full attention to trainer with good response to task but restless and very greedy for food; category 3: penguin being fully focused and paying full attention to trainer and task with perfect response to task., see Rößler et al.^56^) were used for data analysis.

### Stimuli and background noise measurements, and calibration

Signals were measured at a range of amplitudes and evaluated in the time and frequency domains to ensure integrity. For sound check during the trials (with animals’ present), the played signals as well as background noise (Fig. 2) were detected with a microphone (146AE, GRAS Sound & Vibration A/S, Denmark) positioned 75 cm away from the speaker and 10 cm directly above the penguin head and run with a power amplifier (12 AX, 4 channel power and gain module, GRAS Sound & Vibration A/S, Denmark; Fig. 1). The microphone was calibrated every day after each session (Brüel & Kjaer Sound Level Calibrator, Type 4230, 94 dB re 20 µPa at 1 kHz). Sound level during the sessions was manually determined from the recorded audio files of each session via power spectrum of each stimulus (Adobe Audition 3.0. Ink).

After every session, in the absence of the animal, the stimuli and background noise were also measured at the location corresponding to the centre of the penguins’ head with a sound level meter (Nor150, Norsonic, Lierskogen Norway, Tippkemper GmbH, Germany) connected to a 1/2'' preamplifier (Norsonic type 1209) and microphone (Norsonic type 1225; Fig. 2). The unweighted measured spectral noise levels (recorded in dBZ values) were then estimated using Norsonic NorConnect software 2.2. The sound level meter was calibrated by the manufacturer before, as well as by us before and after the data collection period (EXTECH Instruments Sound Level Calibrator, Model 407,744, 94 dB re 20 µPa at 1 kHz). The spatial variation of the sound field within the space of the penguin’s head position (10 cm^3^) during the sessions was less than 3 dB.

### Data analysis

All sound pressure levels at each frequency presented to each bird during the same session type were binned in 5 dB increments as the median in Excel (Microsoft).

Before pooling the session data, we calculated the false alarm (FA) rate for each session (in R; R Core Team, 2020, version 4.0.2 with R Studio, version 1.1.456) and excluded sessions with a FA-rate higher than 35% (for sc and c trial type) from further calculations, as an indication that the decision criteria used by the bird was kept relatively constant^60,63^.

Sessions of one bird, one frequency, one sound pressure level were pooled for each session type separately (c = constant stimulus and sc = method of constants) and used to calculate:

- **d’** (= d-prime, is a statistic of measuring perceptual sensitivity or detectability of the signal, high d’ value good performance, low d’ value poor performance; d’ = Z (hit rate) – Z (false alarm rate), where Z is the inverse of the cumulative normal distribution function (with mean 0 and variance 1; see Gescheider^58^, Chapter 5, for details).
- **β** (criterion beta shows the line of neural activity where the yes/no response limit sits and therefore the bias or tolerance of the animal. The value for beta is the ratio of the normal density functions at the criterion of the Z values used in the computation of d’. If β = 1, it is neutral, if β < 1 it is a low criterion → liberal, β > 1 it is a high criterion → conservative).
- **False Alarm (FA)-rate** was calculated as (sum (n_fa))**/**(sum (n_fa + n_cr)) where n_fa is the number of false alarms at a non-signal trial and n_cr is the number of correct rejections at a catch trial.
- **Hit-rate (hr)** was calculated as (sum (n_hit))**/**(sum (n_hit + n_miss)) where n_hit is the number of hits at a signal trial and n_miss is the number of misses at a signal trial.

Additionally, we determined **accuracy** for each bird, also before pooling in c session type, to ensure that all animals were ready for the more difficult method of constants (sc session type). **Accuracy** was calculated as (n_hit + n_cr)**/**(n_trials), (Abdi, 1987) where n_hit is the number of hits in one session, n_cr is the number of correct rejections in one session, and n_trials is the number of trials in one session.

All statistical tests were done in R for Windows (R Core Team, 2020, version 4.0.2) with R Studio (version 1.1.456). Signal detection theory parameters were calculated with R package psycho and function dprime^60^. Different threshold estimates were calculated using different methods. First, like in traditional psychoacoustics, we chose the threshold limit for each individual and frequency at a 50% hit rate and fixed d-prime at 1 and 1.4 as a threshold determination criterion. Second, to provide a reliable fit to the actual data distribution, we chose d-prime and hit rate limit for each individual and frequency to accurately represent the proportion of response distribution of 50% of the cumulative normal distribution function. By further fitting a psychometric function, plotting d-prime and alternatively hit-rate data as a function of signal level for each frequency and individual, we determined the threshold level by using a probit regression model analysis for the hit rate data and a linear regression model for the d’ data^61^.

To determine variations caused by different threshold determination criterions, we calculated and presented mean and standard deviation values for each threshold determination criterion per animal and frequency, as well as fit of regression model per frequency and animal by slope, intercept, R^2^ and p values (Table 1).

**Table 1:**
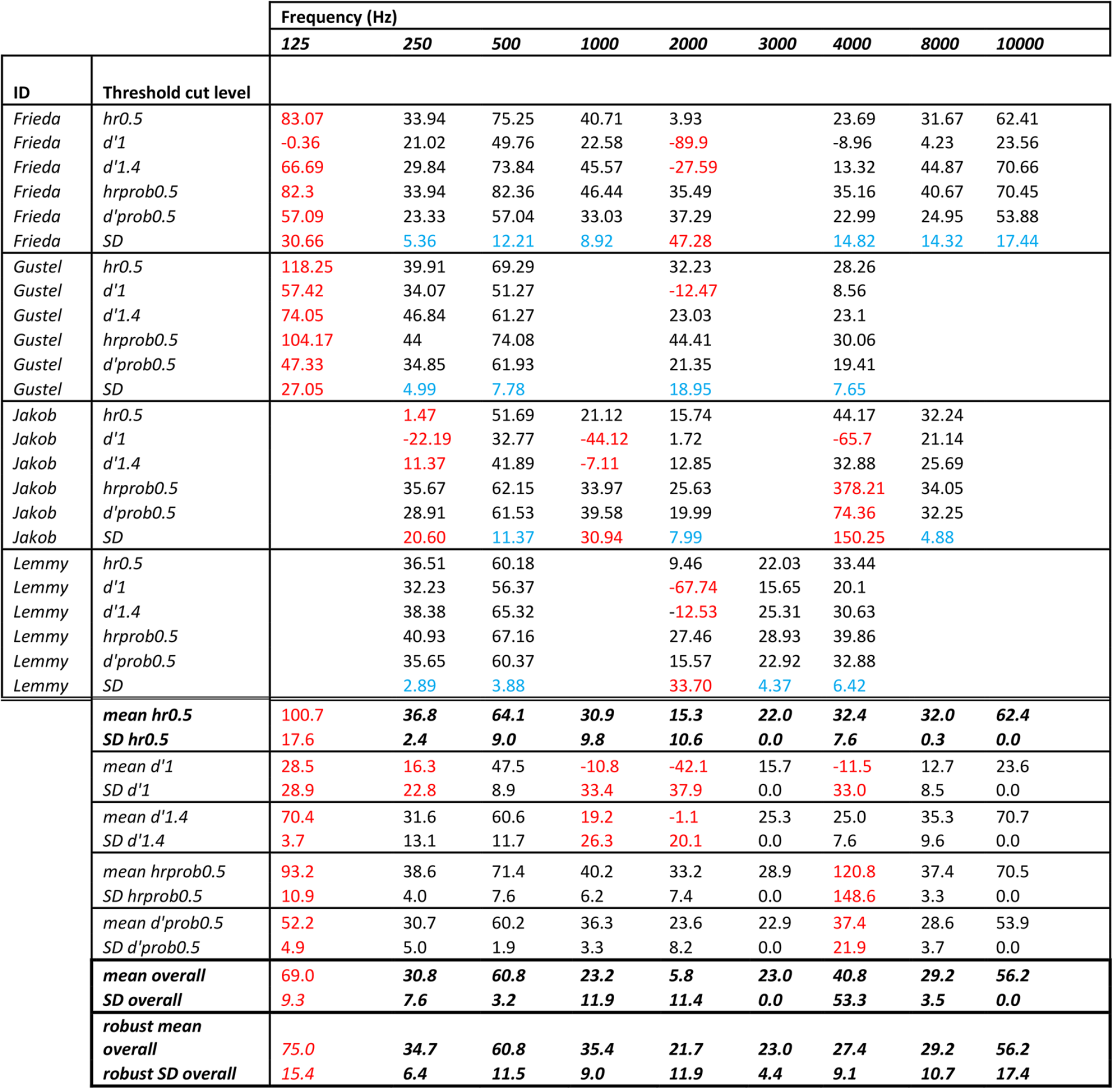
Thresholds (dB rms re 20 µPa) of four Humboldt penguins at 9 different frequencies (Hz) calculated at five different threshold determination criterions (hr0.5 = hit rate 50%, d’1 = d-prime value 1, d’1.4 = d-prime value 1.4, hrprob0.5 = flexible hit-rate value at a level of the 50% probability of the cumulative normal distribution function, ranging from a hit rate of 20 to 80%, and d’prob0.5 = flexible d-prime value at a level of the 50% probability of the cumulative normal distribution function).

Non robust values, due to poorly fitted regressions, are marked in red; Standard deviation of different threshold determination criterions of each animal at each frequency are marked in blue; data in bold and italic were used for the mean thresholds in Fig. 4. For Fig. 7, data of mean and SD of hr0.5 = hit rate 50% were used.

Regressions were regarded as robust, when p < 0.05 and R^2^ > 0.45 indicated significant regressions within an animal and between animals (see Table 2, Fig. S2).

**Table 2:** Model parameters of the linear regression model fitting the d-prime data vs sound pressure level (sc trial type) presented as R^2^ and p values as well as the probit fitted regression of hit rate data (sc trial type) as intercept and slope for each animal at each frequency.

ID	Regression parameter d’	Frequency (Hz)							
		250	500	1000	2000	3000	4000	8000	10,000
Frieda	R^2^	0.569	0.14	− 0.1	− 0.046	Na	**0.468**	− 0.06	− 0.432
	*p*	0.087	0.155	0.501	0.472	Na	**0.006**	0.466	0.799
Gustel	R^2^	**0.527**	**0.663**	Na	0.077	Na	**0.569**	Na	Na
	*p*	**0.025**	**0.003**	Na	0.208	Na	**0.03**	Na	Na
Jakob7	R^2^	− 0.008	0.537	0.014	**0.772**	Na	− 0.129	0.232	Na
	*p*	0.365	0.06	0.335	**0**	Na	0.78	0.301	Na
Lemmy	R^2^	**0.739**	0.401	Na	− 0.024	0.468	0.443	Na	Na
	*p*	**0.0018**	0.054	Na	0.405	0.124	0.062	Na	Na

	Regression parameter hit rate								
Frieda	intercept	− 0.932	− 0.532	− 0.567	0.465	Na	0.156	− 0.11	− 0.372
	slope	0.042	0.014	0.026	0.009	Na	0.015	0.019	0.014
Gustel	intercept	− 0.808	− 1.028	Na	0.059	Na	− 0.482	Na	Na
	slope	0.033	0.022	Na	0.014	Na	0.035	Na	Na
Jakob	intercept	0.009	− 1.211	− 0.123	0.129	Na	0.467	− 2.474	Na
	slope	0.487	0.033	0.029	0.024	Na	0.001	0.092	Na
Lemmy	intercept	− 1.152	− 1.193	Na	0.354	− 0.298	− 0.492	Na	Na
	slope	0.045	0.028	Na	0.015	0.036	0.03	Na	Na

Bold numbers indicate best fitted data.

The number of sessions used in the final data per animal, frequency and sound pressure level varied from 1 to 20 (data of < 35% FA rate, Fig. S2). In the sc trial type, each sound pressure level had a minimum of 5 trials per session and frequency, and a minimum of 20 trials per frequency when pooled, with a minimum of 4 signal trials per frequency present. In the c trial type, each session had a minimum of 20 trials per frequency and sound pressure level with a minimum of 10 signal trials when pooled (for more detail see supplementary data).

## Results

**Fig. 3:**
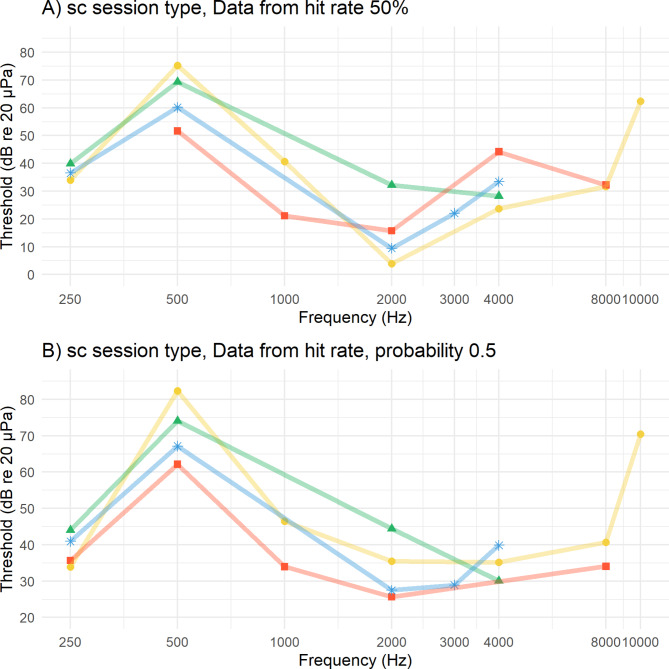
In-air psychoacoustics audiogram for the four Humboldt penguins. The hearing thresholds (individual symbols) were derived from signal detection theory at A) 50% hit rate, B) 50% probability of cumulative distribution function of hit rate. Yellow Dot = Frieda (female), Red Square = Jakob (male), Green Triangle = Gustel (female), Blue Star = Lemmy (male). The same-coloured straight lines connect the data points of the individuals. Not all frequencies were usable for all individuals and therefore missing in the figure. See supplementary figures for d’ audiograms of individuals.

The behavioural audiograms indicate a functional hearing range from 0.250 to 10 kHz (Fig. 3, Table 1). The best hearing sensitivity (2 kHz or 4 kHz) depended on individuals and which threshold determination criterion (50% hit rate or 50% probability of cumulative distribution function of hit rate) was taken (Fig. 3, Table 1). When the auditory threshold was determined by the 50% hit rate (most common for psychophysical audiograms), mean most sensitive threshold was at 2 kHz at 15 dB rms re 20 µPa (SD ± 10.6 dB, n = 4). In contrast, the poorest hearing sensitivity was always found, independently of the threshold determination criterion or animal, at 0.500 kHz (Fig. 3, Table 1). When the auditory threshold was determined by the 50% hit rate, a rapid decline of sensitivity showed at 0.500 kHz for all animals a mean value of 64 dB rms re 20 µPa (SD ± 9 dB, n = 4). Again, independently of the threshold determination criterion or animal the 0.250 kHz was consistently detected at lower intensity than the 0.500 kHz (Fig. 3, S4, Table 1). When the auditory threshold was determined by the 50% hit rate, mean sensitivity at 0.250 kHz was 37 dB rms re 20 µPa (SD ± 2.4 dB, n = 3; Fig. 3, Table 1).

**Fig. 4:**
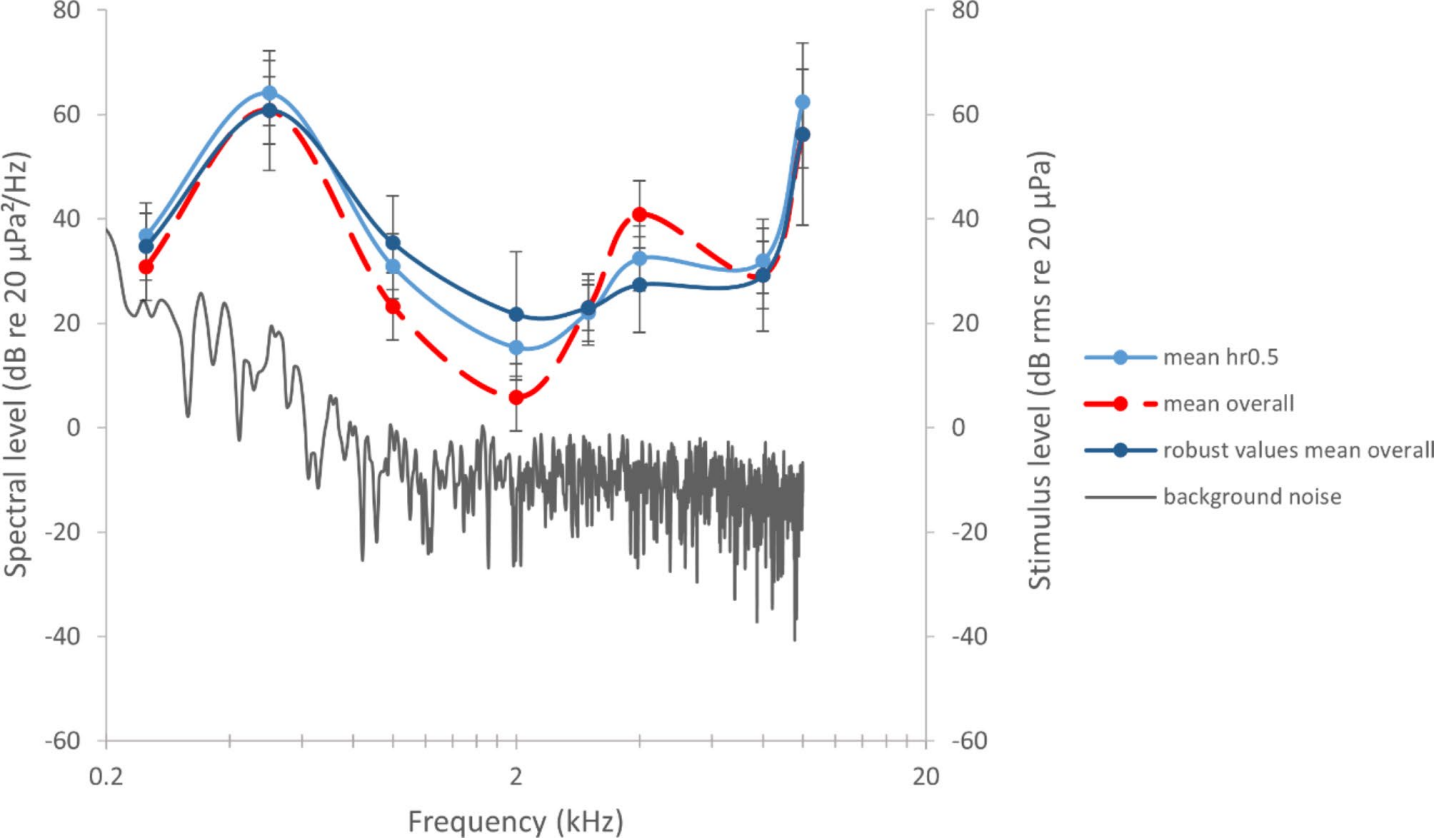
Audiogram of mean thresholds of all four animals from different cut off limits (mean hr0.5 = mean values at threshold cut level of 50% hit rate, mean overall = mean data from all cut off levels including all animals also if data had outliers, robust values mean overall = mean data of only the most robust data points were included, amount of data points changed for animal and frequency; for specific data used see Table 1).

Expected from psychophysical audiogram measurements of other aquatic bird species, the means audiogram shape of the Humboldt penguins had a U-Shape between 0.500 kHz and 4 kHz (Fig. 4). But an unexpected rapid decrease in sensitivity at 0.500 kHz with increase in sensitivity at 0.250 kHz and in two penguins a slower decrease in sensitivity above 4 kHz (Fig. 4, Fig. 7). The means absolute threshold shape stayed the same independent of threshold determination criterion (Fig. 4, S4 and Table 1).

Response variability at each frequency was high but consistent for each animal. Due to a high intra-individual variability between sessions, it was not possible to gather the necessary amount of consistent data points for each sound pressure level over time for all frequencies for all animals (Fig. S2).

**Fig. 5:**
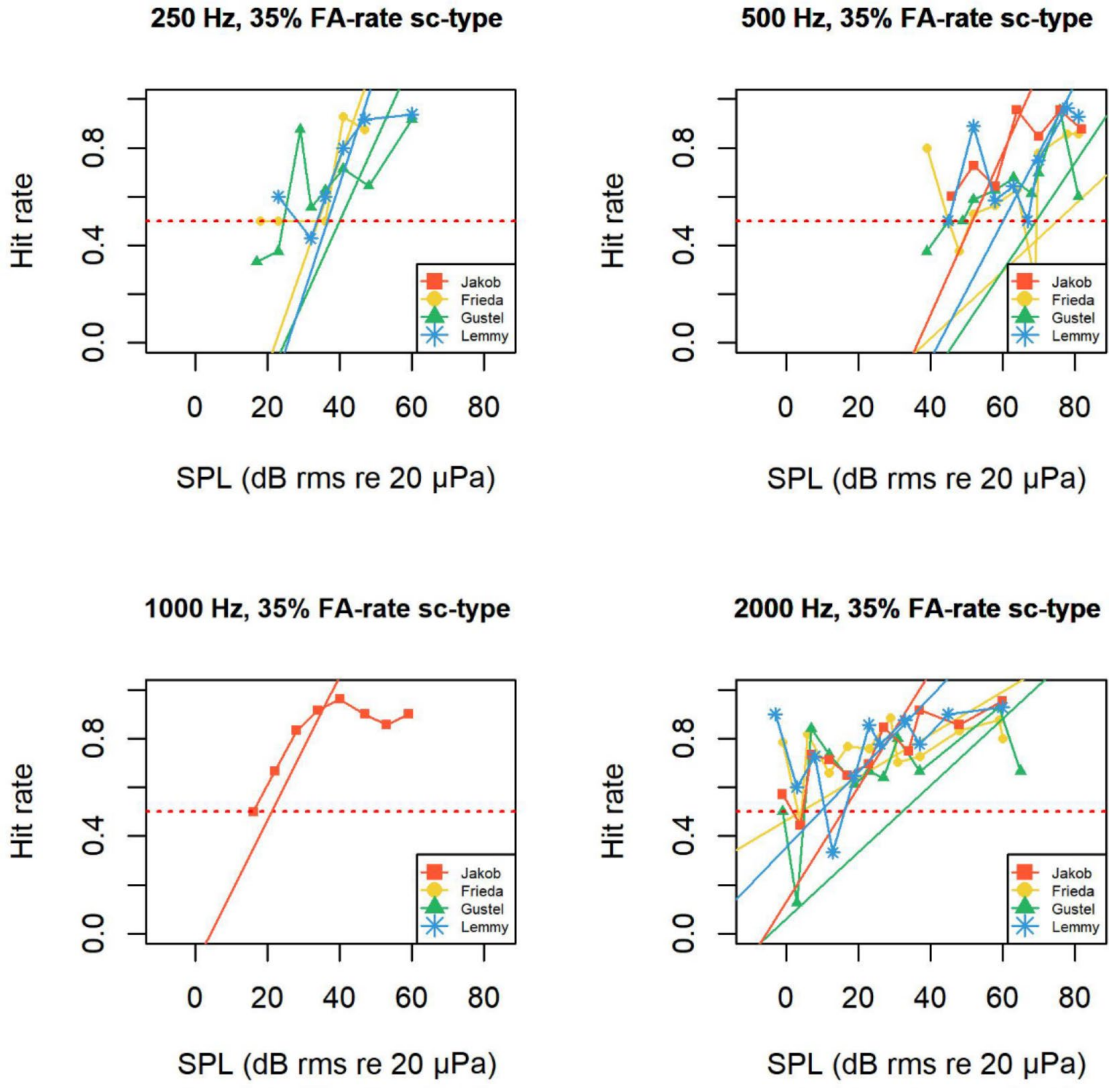
Hit rate as a function of sound pressure level (SPL) shown per frequency for one to four animals after cleaning the data. Psychometric functions generated by probit fitting for each tested frequency and individual. See supplementary material Fig. S5 for other frequencies tested in the study.

Psychometric functions and regressions from fitted models were often poor fitting or had a very flat slope (Table 2) which made determination of the interception point for threshold determination challenging. High variability in accuracy was seen within each animal between frequencies as well as between animals at the same frequency (see intercept values of Table 2, Fig. S1). A steeper slope like a higher d’ value means a stronger relationship between the ability to detect the sound depending on different sound pressure levels (Table 2, Fig. S3). Jakob most often showed the best discrimination capabilities paired with the highest precision of the four penguins (Fig. 5, Fig. S3).

Data from all individuals showed an inconsistency in the fitted probit function form between frequencies (Fig. 5). The interception point differed significantly within one animal at one frequency (Fig. 3, S4) depending on which determination factor was taken (50% probability of cumulative distribution function of hit rate and d’, 50% hit rate, d’ 1 or d’ 1.4).

Psychometric functions showed overall similarities between animals within one frequency although all individuals showed high hit rate variation between sound pressure levels often with a reoccurring increase of detectability at low sound pressure levels and decrease at highest sound pressure levels. This made functions ill-fitting and very difficult to determine adequate intercepts (Fig. 5 and Table 2).

At some frequencies (e.g., 0.250 and 0.500 kHz), all animals showed a low inter – and intra-individual variance (Table 1, 2 and Fig. 3, 5) indicating reliable results.

The signal detection theory parameter beta (β) showed that each animal criterion was generally unbiased at the 2 kHz tone and c type data (mean values: Jakob = 0.9, Lemmy = 1, Frieda = 1, Gustel = 0.9). All four penguins reacted more conservatively when the stimulus level became lower and more liberally when the stimulus level was high. They seemingly became more conservative at the low and high end of frequencies.

In the sc type trials at 2 kHz tone, the animals had a minimal biased criterion beta (β) of a conservative answer behaviour (mean values: Jakob = 1.4, Lemmy = 1.2, Frieda = 1.5, Gustel = 1.4). All four penguins reacted even more conservatively when the stimulus level became lower. Therefore, all animals changed their behaviour to a more conservative one when tasks became more challenging independently of session type (Fig. S2).

**Fig. 6:**
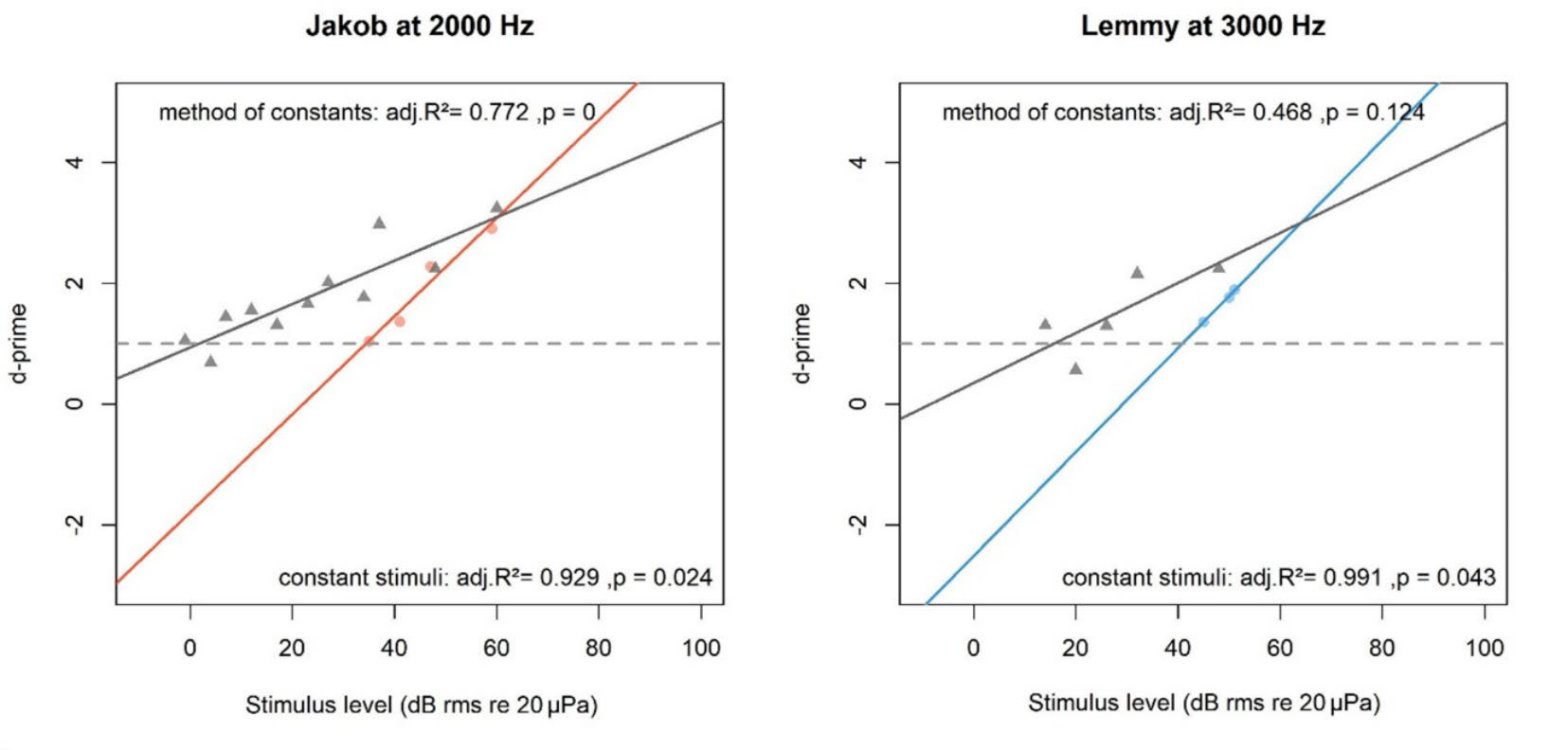
Sound pressure level as a function of d-prime plotted with a linear regression for each session type in an example of two different animals at two different frequencies. Each coloured dot represents pooled sessions per sound pressure level with constant stimulus (c) session type and each grey triangle representing pooled sessions per sound pressure level of the methods of constants (sc) session type. Grey horizontal line is at a commonly used d-prime value of 1. Session types were not combined for threshold determination due to different slope steepness. Adjusted R^2^ and p values are seen in the plot for each session type.

In accordance with psychophysical methods known from the literature^53,54^, slopes of the regressions were steeper in the c session type than in the sc session type. Therefore, thresholds detected with the sc session type were found to be consistently lower (Fig. 6) but also had higher residual variation (Fig. 5, 6).

## Discussion

For the first time, psychophysical hearing thresholds were measured here in penguins. All four penguins showed that the task was within their cognitive abilities with expected species-specific best hearing frequency. However, a surprisingly conservative answer criterion was found for more difficult tasks with high individual variability in precision and sensitivity. Our results show some robust findings for absolute penguin aerial hearing thresholds, but also encompass some surprising findings.

### Comparison to other aquatic bird species

**Fig. 7:**
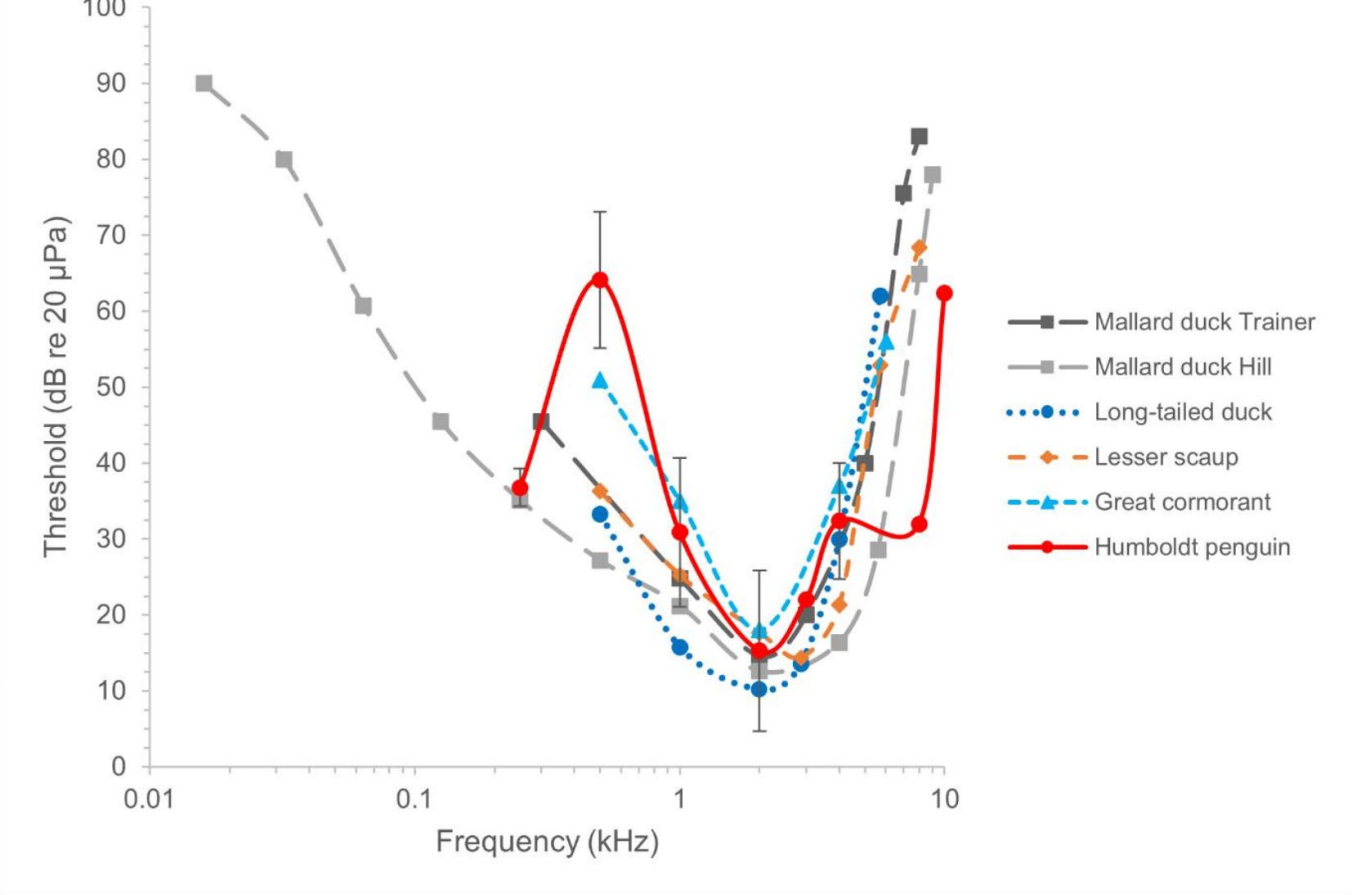
Audiogram of psychophysical hearing thresholds of currently available water birds. (Data acquired from Hill, 2017; Maxwell et al., 2017; Therrien, 2014; Trainer, 1946 (in Hill, 2017)) now also including the Humboldt penguin (mean 50% hit rate ± SD, n = 1 – 4, see Table 1) from this study.

From the current study, Humboldt penguins were found to have the highest hearing sensitivity within a frequency range (between 1 and 4 kHz with 2 kHz as their mean most sensitive frequency) comparable to that found in other terrestrial (e.g., pigeons)^45,64^ and aquatic bird species^20,45,48,49,64–69^. This also aligns with findings of the cochlear potentials of African penguins^54^. Surprisingly, the roll off at the high frequencies appeared to be slower than in other physiological and behavioural studies on penguin and water bird species^48,49,54^. Moreover, the roll off at lower frequencies was highly irregular with a steep roll off towards 0.500 kHz of all four penguins but a common robust increase in sensitivity at 0.250 kHz for three of the four animals. Accuracy of all birds was already low at 10 kHz and 0.500 kHz at the loudest possible sound pressure level of the set up. The hearing thresholds of the four penguins showed best alignment between 0.250 kHz and 2 kHz. The mean best sensitivity values are comparable to other behavioural audiograms measured in aquatic birds (Fig. 7;^47–50,64^).

### High sensitivity at 0.250 kHz after very low sensitivity at 0.500 kHz

The ability to detect low frequencies of 0.250 kHz likely has ecological implications. Penguins are highly social animals and use their visual and auditory senses to constantly interact with their surroundings for orientation, to detect threats or assurances from predators, heterospecifics, partners, chicks or the geophysical environment^70^. Environmental sounds surrounding penguins, like wind and waves, have most power below 0.500 kHz^71^. The fundamental frequency of the Humboldt penguin vocal repertoire is below 0.500 kHz, but their minimum and maximum frequency range varies between individuals. Vocalisations of Humboldt penguins span from 0.260 ± 0.16 kHz to 2.92 ± 1.21 kHz for ‘haws’ (call to keep in contact with partner or juvenile animal when one is in water and one on land), and from 0.41 ± 0.30 kHz to 5.57 ± 1.82 kHz for ‘bray’ calls (call to attract a mate or advertise territory) with high individual variation^72^. This high individuality also makes it easier to identify and locate their colony and mate when returning from foraging^43,73^. To identify an individual over greater distance, low-frequency hearing sensitivity is important as lower frequencies have lower propagation loss^74,75^. Low frequency hearing is also supposedly important for reproduction as nest reared chicks (Adélie penguins (*Pygoscelis adeliae*)) react especially well to the low frequency part of the spectrum and can identify their parents pitch with an accuracy of 25 Hz^76^.

### Changes in detectability at variable sound pressure levels tested

Strangely enough, all our animals showed a low detectability at the highest sound pressure levels and again an increase in detectability below 50% threshold. This unusual result made analysis highly difficult and model slopes flat or ill fitting. Even after filtering out high false alarm rates, low trial sessions and possible biased sessions due to seasonal variation these phenomena remained present. We therefore suggest several plausible explanations.

### Anatomical, neurological, and physiological explanations

Hearing conspecifics in a densely packed colony is likely as important as filtering out conspecifics to detect important information at a certain time (e.g., the individual specific call of the partner at the time of arrival). Previous studies have showed that penguins have a well-developed system to orient in the environs and detect conspecifics in a noisy crowd^77–79^. Penguins can discriminate signals in noise extremely well. This discrimination capacity varies between species, ranging from 0 dB signal to noise ratio (SNR) in nesting Adélie penguins^76^, down to -6 dB SNR in open space King penguins^77^. How acoustic filtering mechanisms work at a neurological, mechanical and/or physiological level in burrowing Humboldt penguins remains, however, unclear.

Frogs, bats and cetaceans are able to actively adjust their auditory sensitivity to prevent damage to their ear from either their own emitted sound signals, or from external loud surrounding sounds^80–83^. Auditory adjustments in those species have been shown to be regulated by cognitive, neural, as well as mechanical middle ear muscle processes^81,84–87^. Temporal threshold shifts and the mechanical processes depend on the modification of the auditory sensory organ through evolutionary selective pressure to best process communication or navigation signals vital to survival and reproduction. Habitat therefore has a major influence on the evolution of spectral peripheral auditory filters^88^.

Evolutionary pressure also leads to niche-specific cerebrotypes in birds (related primarily to behaviour and ecology) with the strongest evidence provided by similarities in prey capture^89^. Cognitive processing of sound in turn, has been shown to change as ear anatomy evolves, especially when considering the changes from aquatic to terrestrial lifestyles and the independent convergent evolution of ear morphology^4,90^.

Due to their water related environment, aquatic birds have been found to be able to control the outer opening to the ear and prevent water from entering^17,19^. The external meatus can also have an influence on sound properties for attenuation or gain depending on its tissue specificity^18^. How wet feathers or in water physiological ear mechanisms, when the penguin came straight from the water to the testing chamber, could decrease sensitivity or change mechanisms by closing off the canal, is unclear^91^. In psychophysical in air tests of ringed seals, the seal’s thresholds showed a 34 dB decrease of sensitivity when tested at the water surface compared to when the thresholds were collected in an acoustic chamber during extended periods hauled out^29^. However, the authors could also not explain why this difference between the conditions affected auditory capabilities.

Anatomical features of the middle ear such as columella-length and tympanic membrane-to-footplate area ratio were previously best explained by the affiliation to an ecological group. Penguin ears show unique morphological adaptations, such as a negative relationship between tympanic membrane-to-footplate area ratio and head mass, a shorter extra-columella and a smaller area of the columella footplate^17^. Tension and geometrical orientation of complicated mechanical middle ear lever structures, have been shown to be responsible for changes in the resonance system in the bird ear. This includes the extra-columella (three cartilaginous structures: extra-stapedius (ES), infra-stapedius (IS) and supra-stapedius (SS)) and the tympanic processes of the extra-columella that connect the long single shaft of the columella to the inner surface of the tympanic membrane^22^. In pigeons and chickens, translational motions in the low frequencies (between 0.250–4 kHz) could be detected and the rotational angle between middle ear structures changed mechanically. This change leads to an increased energy transfer causing potentially more acute hearing sensitivity^21^.

The penguin specific reduction in length of the extra-stapedius and reduced columella volume might explain less energy transfer loss due to a lower mass of the transmitting apparatus which increases sensitivity especially at higher frequencies and may lead to the slower roll off at higher frequencies seen in our study^18^. The shorter extra-columella with reduced levers is supposedly stiffer and would consequently increase the transfer of all frequencies. The smaller tympanic membrane area with a flattened shape, a smaller and stiffer extra-columella and the smaller columella footplate, makes the transfer of higher pressure possible and would consequently also lead to a slower roll off in sensitivity at higher frequencies, as found in our study^21,92–94^.

Additional tension change of a passive or active nature from the columellar muscle could potentially influence vibrational capacities. Contraction of the middle ear muscle changes both the tension of the tympanic membrane and the position of the lever system of the middle ear. This means that when the tympanic membrane is tensed the columella and the extracolumellar cartilage is moved towards the tympanic membrane, and the base of the columella moves away from the vestibular window. Activation of the columellar muscle should therefore decrease the amount of sound energy transmitted to the inner ear^95,96^. Applying this mechanism to our results suggests that a decreasing hit rate at higher sound pressure levels may represent a protection mechanism for penguins against overstimulation from outside (such as low-frequency wind noises or noisy conspecifics) and from their own vocalisations. This mechanism would also explain the noticeable increase of hit rate at lower sound pressure levels and at ecologically relevant frequencies, like a fine-tuning mechanism^97^.

When comparing penguins to other birds or amphibious living animals, it is necessary to consider the purpose of the middle ear as an impedance matching mechanism of air to the cochlear fluids at the interface of middle and inner ear. Too much amplification creates physiological and morphological conditions that can be as ineffective for sound transmission as too little amplification. At frequencies beyond the processing capacity of the basilar papilla of the inner ear, less of the input energy from the middle ear is accepted by the inner ear, leading to a rise in impedance that causes the lever system to bend and to be reduced in its efficiency to transmit the signal adequately^92,94^. Our results show a decrease in sensitivity at 0.500 kHz and 10 kHz commonly observed in birds, which most likely shows evolutionary processing capacity restrictions of the basilar papilla also in penguins^39,96^.

Research on infrasonic hearing capabilities in birds found that the cochlea’s inertial fluid filled pathways or canals, and their impedance deviations were suggested to cause changes of resonance capabilities in birds^98^. Fluid spaces of the inner ear and its interchange with the brain cavity has been shown to have an impact on low frequency impedance. At the apex of the cochlea, at the so-called apical interscalar canal, communication of the perilymph fluid between the scala vestibula and scala tympani occurs^99^. The cochlear aqueduct is an additional third fluid-filled canal which allows transmission between the perilymph of the inner ear and the cerebrospinal fluid of the brain cavity. It is present in all birds, but most enlarged in penguins^17,100,101^. The cochlear aqueduct serves as a connection between the ear and the brain and supposedly acts as a low-pass filter, and therefore is relevant for low frequency hearing. Low frequency impedance through lower stiffness and shunting of fluid paths of the inner ear can be reached by a large round window, large cochlear aqueduct, large interscalar canal, and a soft-tissue cochlear partition^98^. A large cochlear aqueduct was also previously proposed to enhance sound sensitivity through transmission of fluid vibrations within the brain cavity to the inner ear^102,103^. The increase in size of the cochlear aqueduct accompanied by considerable reduction of cranial air volume in penguins, most likely for dive related pressure protection, would also increase reduction of acoustic impedance while under water and a general increase of acoustic transfer through cerebrospinal and perilymph fluid vibrations at lower frequencies.

Another penguin specific anatomical feature possibly responsible for the observed changes in hearing sensitivity is the complete closure of the interaural canal and of the interbullar passage. This decoupling of the ears in penguins most likely has an influence on how sound is processed in the central auditory system as well as how low - and high - frequencies are differently processed^23,104–106^. Low frequency sound detection is used for navigation and orientation purposes^107^. Mechanisms of directional hearing and low frequency detection may have their origin in accompanied specific morphological adaptation in the ear and cranium.

Finally, multiple neural structures involved in hearing capabilities may also influence absolute auditory thresholds and psychophysical behaviour^108^. Neurological processes starting in the periphery with hair cells in the cochlea propagate to the nervous system and end with the processing in the brain.

Information flow in the nervous system is bidirectional. Stimulus information from the sensory organs is transmitted to the nervous system, while neural signals are also carried back from the brain to the periphery. This feedback can modify responsiveness of the receptive sensory cells in the cochlea and thus prolong regulation effects before and after a stimulus^39^. Loud sounds would therefore activate a top-down attenuation mechanism towards less responsive auditory structures for following sounds and change the perception of a sound early in the auditory process^39,109^. Ecological specialisation for activities such as nocturnal hunting in owls or cave dwelling in oil birds have shown specialisation for different kind of hair cells across different areas of the papilla. These specialisations include different afferent and efferent innervation patterns as well as changes in the hair – cell bundles orientation across the papilla to detect extremely small amplitude sounds or especially high frequency signals^104,110–112^. In African penguins (*Spheniscus demersus*) general auditory structures in the cochlea are shown to be organised like in other common birds and show similarity to caimans^113^. How and if a specialisation for under water pursuit in diving birds possibly developed in the auditory neural system is so far mainly speculation due to a lack of supportive evidence.

In general we can state that the current information on anatomical features would benefit from further research into the whole mechanism from the sound reception at the tympanic membrane, including fluid dynamics of the inner ear and the semicircular canals including the cochlear shunt to the final neurophysiological trait of sound reception in the hair cells using fine-scaled finite-element modelling, like recently presented for *Eudyptula minor* (Little blue penguins)^114^.

### Personal traits explanations

Penguins show a range of personality traits and adaptations at a species^115,116^, population^117^ and individual level^118–122^. Therefore, in a colonial seabird context, the development of different personality traits for successful reproduction and survival strategies should be considered in behavioural testing methods.

High intra-individual consistency of variance shown in our results within one frequency but constant differences between individuals across frequencies might depict personalities and personal specific energy distribution in task execution^119,121,123,124^. From our direct observations of the behaviour of penguins in their colony, during training and data collection sessions, Lemmy appeared to be a very active, curious, and investigative penguin. He was bold and aggressive towards unknown people and exhibited quick movements. Lemmy mostly chased shadows for play. He always demanded lap sitting time and stroking with preening from well-known humans. In contrast, Gustel’s demeanour was very calm and passive. It took a lot for her to show aggressive behaviour. She showed aggressive behaviours only towards Frieda due to mating choice competition, and foreign human intrusions in certain areas. She was curious especially towards specific objects like shoestrings or hairbrushes. It took her more time to react to commands. She had times when she was very impatient, jumpy, and erratic during sessions. Gustel mostly chased moving objects for play. Jakob radiated poise. He picked up on new commands quickest and was the most attentive. He executed movements precisely and consistently. He showed the most logical control over situational behaviour. Jakob did not play much but overlooked play time and enjoyed company. Frieda was shy and fled when new people or situations occurred. She showed preferences to people and locations, with changes in thresholds resulting in quick bites and touchy behaviour. When she felt safe, she became very playful and demanded play time and discovery time for new locations. She mostly chased light reflections for play.

The described personalities support the type of variability in the psychometric functions and animal criterion in our data. It implies that different personalities have an influence on decisions and flexibility in behaviour. Studies in dogs have shown that variability is most likely caused by the fact that coexisting individuals employ different decision rules to choose between the behavioural alternatives. Where more responsive individuals use a fine-tuned rule that conditions the behaviour to the most common situations, unresponsive individuals employ a general-purpose rule that does not distinguish between circumstances. Answer decision (Go or No-go) and cooperation can therefore be a by-product of stable individual differences at the level of behavioural organisation^123,125^. When comparing the differences of slopes of the psychometric functions in our results we detected differences in steepness between individuals. The detection task appeared more difficult for Frieda (shy) than for Jakob (quiet). When the psychometric slope was steeper, the animal showed a stronger reaction to smaller stimulus, therefore indicating better distinction capabilities. All animals were within their cognitive abilities but variation between individuals in one frequency and the stability when compared between frequencies indicates individuality in cognitive or sensory capabilities^126–128^. Additionally, individuality in reward processing might have influenced variability within one animal and between animals^129^.

### Environmental explanations

The high variability between sessions of all penguins, independent of the method used, most likely reflects the penguins’ daily and very seasonal concentration window which reduced greatly with age. This was especially true after Jakob took a partner as he participated less often in training sessions. Overall, the motivational state of the birds was not the same throughout one year and between individuals^56^. The seasonal changes of an animal’s behaviour are often in alignment with hormonal changes. Hormone levels have also been reported to influence auditory and cognitive capacities^130–134^. For our penguins in air hearing this might imply that their attentiveness within a session was different in different seasons. This change in cognitive attentiveness could result in poorer performance in the signal detection task. Additionally, when motivation and concentration levels were considered high during the sessions, it might be that during certain periods of mating, breeding, or foraging (pre moulting), auditory behaviours possibly changed due to the biological relevance of seasonal auditory sensitivity change.

Seasonal influences not only include direct changes to hormones but also indirect changes through environmental factors such as temperature and light^56^. It has been shown that the pineal gland, which regulates melatonin levels, is influenced by temperature and light levels and has a direct influence on chronical thermoregulatory processes^135^. Body temperature and metabolism in birds exhibit not only seasonal but also daily variation as a direct effect of changes to ambient temperatures but also as an endogenously controlled process^136,137^. Penguins have a complex thermoregulatory system and can specifically regulate and change the blood flow to certain body parts for precise thermoregulation^138,139^. How this blood flow changes around the ear during cold periods for example, or how increases or decreases to blood flow (also during additional digestive processes) might influence hearing and processing capabilities has not been reported for penguins, yet. The animals in our study live outside in an unregulated habitat where temperatures and light levels in the data collection and training area changed dependent on outside environmental conditions.

### Methodological explanations

The method with constant sound pressure level stimulus had a better goodness of fit but as expected a steeper slope than the method of constants (with randomised sound pressure levels per session). The constant sound pressure level was a useful method to determine if the penguins understood their task and determine individual reinforcement contingencies^140^. With the constant sound pressure level stimulus, it was also easier for the trainer to detect false patterns and reactions towards false stimuli (e.g., the trial light). This method was crucial to make sure that the animals were ready and understood the task at hand. The more consistent results with the constant sound pressure level stimulus also support the findings of the more variable method of constants.

The fast and irregular behaviour of the penguins depending on daily and seasonal level, and personality made it difficult to gather the same amount of trials per session and frequencies^56^. We could see variability in concentration and motivation within one session depending on food saturation and onset of digestive processes, which was counteracted by waiting periods and manual dismissing of trials but could not be fully excluded from the data collection process, especially with the method of constants. This made the process to gather the same amount of data at all sound pressure levels very challenging, time consuming and partly impossible. Due to the randomised sound pressure levels at one session, the number of trials for all sound pressure levels per session could not be controlled, as a session stopped when the animal stopped cooperating. This may have led to a higher variability in our collected data with an increase in statistical uncertainty.

In addition, not all penguins were at the same training level at the same time. Jakob was ready and high in accuracy very early in the data collection process, but later started to mate which led to a loss in data points at other collected frequencies and possible increase in variability. Gustel, on the other hand, took longer to station still and had several periods during data collection where she was highly impatient, and concentration levels were low which led to unusable sessions and higher variability in the beginning and in between data collection period^56^.

Most puzzling was the increase in signal detection at low sound levels, after an expected decrease of detectability towards the 50% hit rate. We eliminated the possibility of signal interference but considered that a possible influence might have been the set position of the threshold sound pressure level for a session’s sound pressure level range. Setting the threshold level of some sessions at a lower level might have changed the concentration level and increased detectability, as manipulations of stimulus context can evoke changes in both decisional and sensory processes^141^. It has been shown that the stimulus SPL-ranges of a session can influence loudness judgments due to a response bias in judgement. Thus, an identical stimulus can receive a different judgement when it appears in different stimulus sets^109^. We tried to prevent this by inserting manual silent intervals but there are no previous studies to judge whether those worked sufficiently well. Another factor of loudness judgement is the correlation of the sound pressure level position relative to an internal adaptation level, which is itself determined by the distribution of the stimulus levels in the set (adaptation-level model;^142^). Thus, a given stimulus will be judged as weaker by an individual when it falls below the internal adaptation level than when it falls above that adaptation level. Different internal adaptation levels for each individual animal possibly also explain some of the inter-individual data variation. Selective attention can change depending on age and the ability to inhibit interference during the test process^143^.

The same reason that makes psychophysical tests so great, that is the inclusion of all physical and psychological aspects of the whole animal, is also why we cannot fully distinguish the reason for the increased hit rate below a presumable threshold. It is likely that the details in the methodological approach might need further adaptations for seabirds to take species specific behavioural strategies for survival and reproduction more into consideration.

## Conclusion

Humboldt penguins in air hearing thresholds were determined and the most sensitive hearing frequency is comparable to other aquatic bird species. Aquatic morphological adaptations of the middle and inner ear do not seem to have a negative effect on hearing capabilities in air but possibly change sensitivity at certain (roll off at higher and specific lower) frequencies in air. We could not determine an acoustic or obvious methodological error for the increasing hit rate at the lowest sound pressure level or at low frequencies and therefore suggest a biological effect in the form of an anatomical, physiological, or neurological adaptation of penguins.

Our study provides supporting evidence that penguins have retained the ability to perceive quiet low frequency airborne sounds despite, or possibly because of adaptations related to their amphibious colonial lifestyle. Species and individual specific tissue flexibility, muscle activation and frequency dependent impedance change due to inner ear fluid flow could explain the increase in sensitivity at 0.250 kHz for our penguins. Now, our results on the in-air hearing sensitivity of penguins must be extended under water.

## Electronic supplementary material

Below is the link to the electronic supplementary material.

Supplementary Material 1

Supplementary Material 2

Supplementary Material 3

Supplementary Material 4

Supplementary Material 5

Supplementary Material 6

Supplementary Material 7

Supplementary Material 8

## Data Availability

The datasets generated or analysed during this study are included in this published article [and its supplementary information files].
