# Supplementary material for: Biological relevance and methodological implications of unexpected hearing thresholds in a diving bird
Source: Sci Rep. 2024 Dec 23;14:30592. doi: 10.1038/s41598-024-82942-2 (PMC11666583; doi:10.1038/s41598-024-82942-2)
Supplement: Supplementary file 7 — Supplementary Material 7 [file 41598_2024_82942_MOESM7_ESM.pdf]

Frieda, 125 Hz ,35% FA−rate sc−type

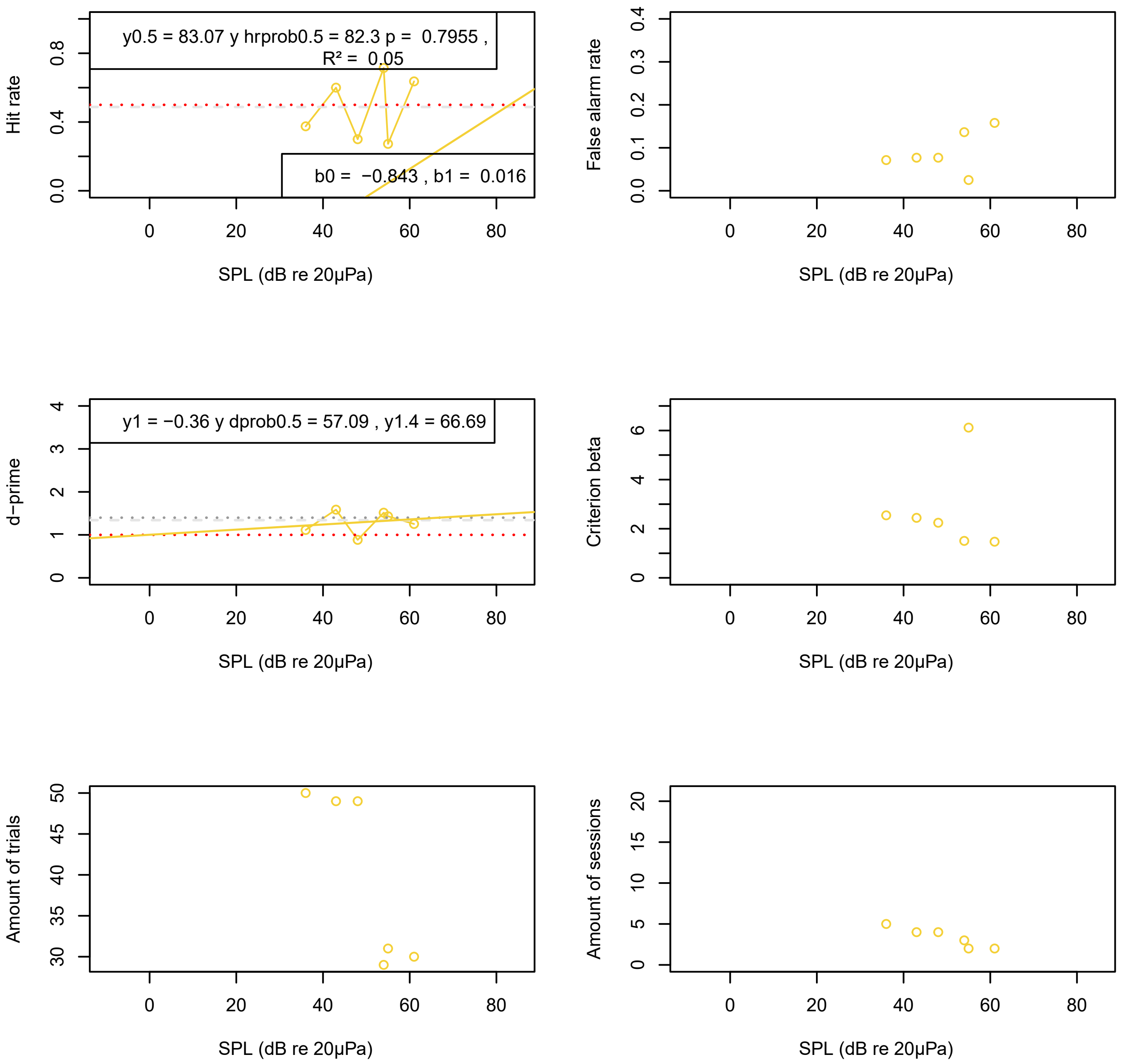

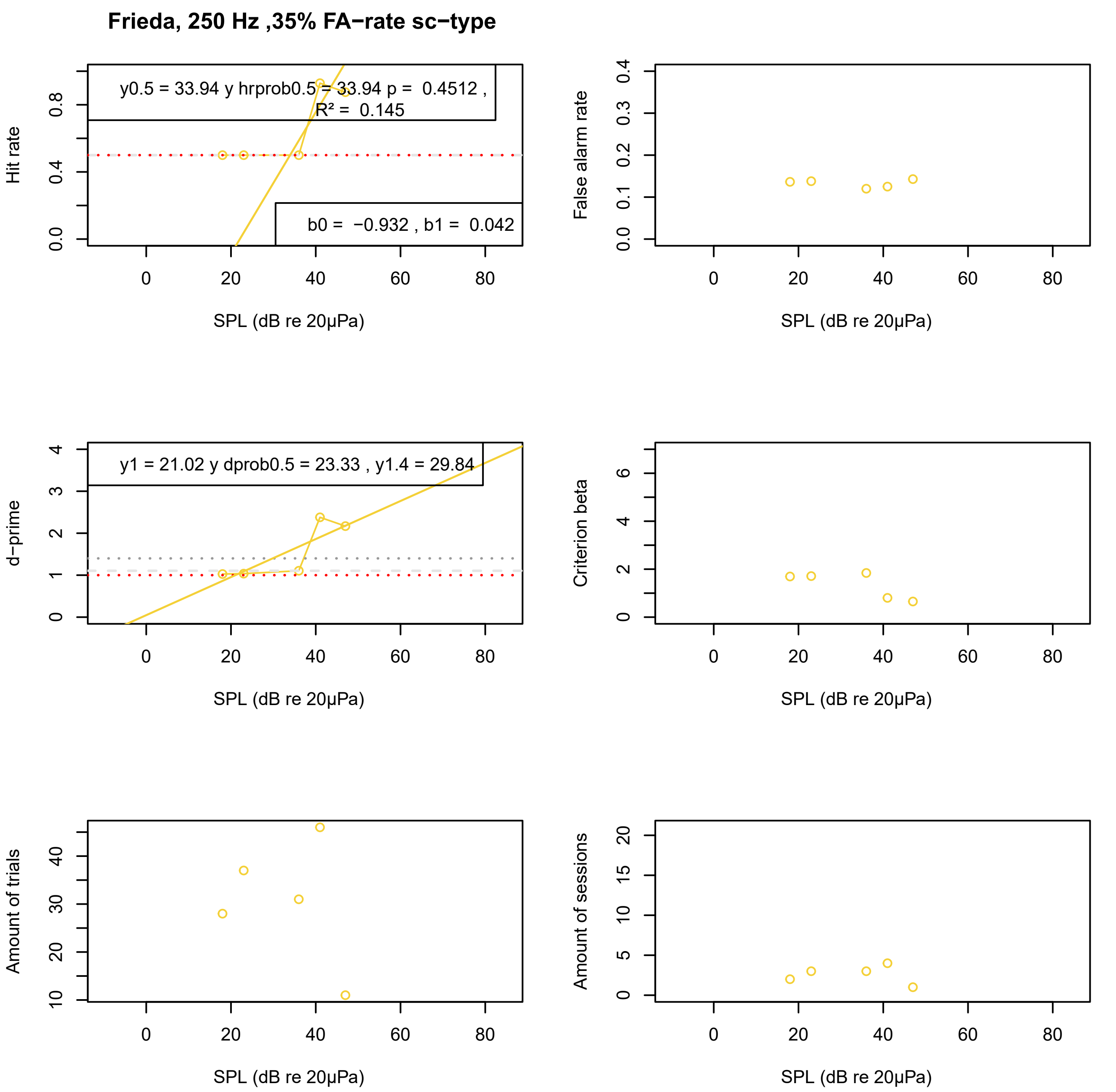

Frieda, 250 Hz ,35% FA−rate sc−type
y0.5 = 33.94 y hrprob0.5 = 33.94 p = 0.4512 , R² = 0.145
b0 = −0.932 , b1 = 0.042
Hit rate
False alarm rate
y1 = 21.02 y dprob0.5 = 23.33 , y1.4 = 29.84
d−prime
Criterion beta
Amount of trials
Amount of sessions
SPL (dB re 20µPa)

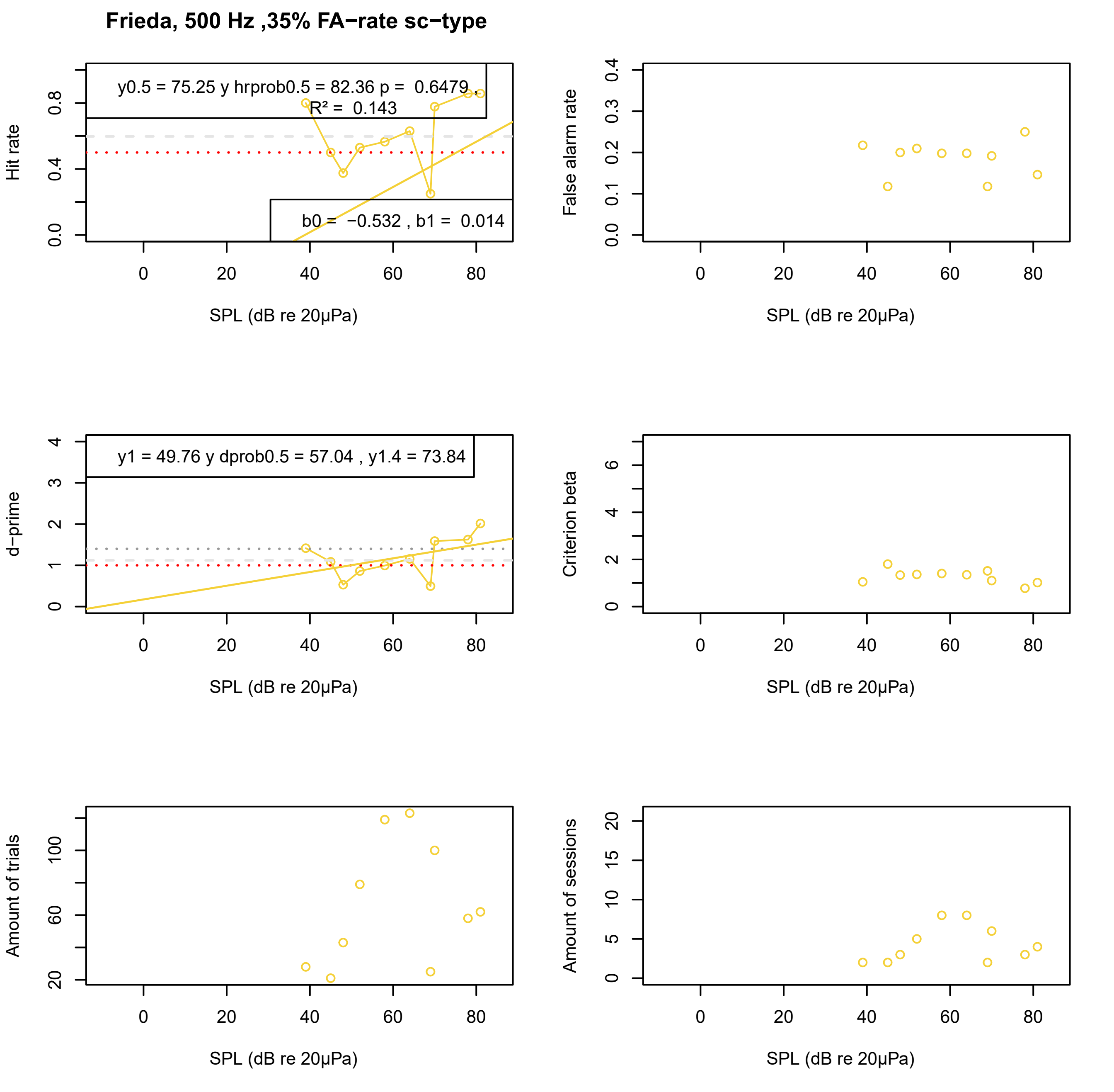
Frieda, 500 Hz ,35% FA−rate sc−type
y0.5 = 75.25 y hrprob0.5 = 82.36 p = 0.6479 ,
R² = 0.143
b0 = −0.532 , b1 = 0.014
Hit rate
False alarm rate
y1 = 49.76 y dprob0.5 = 57.04 , y1.4 = 73.84
d−prime
Criterion beta
Amount of trials
Amount of sessions
SPL (dB re 20µPa)

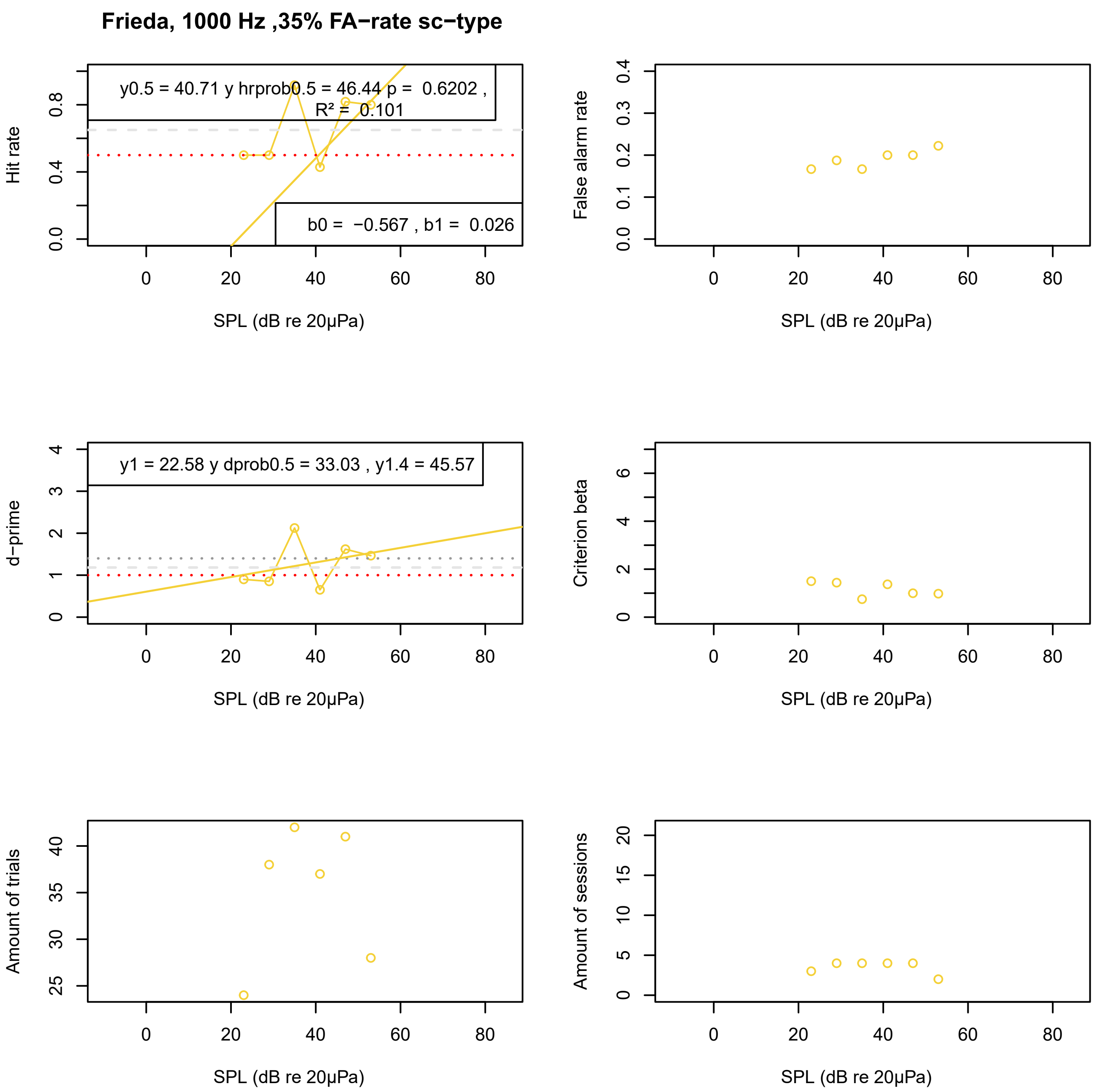
Frieda, 1000 Hz ,35% FA−rate sc−type
y0.5 = 40.71 y hrprob0.5 = 46.44 p = 0.6202 , R² = 0.101
b0 = −0.567 , b1 = 0.026
Hit rate
False alarm rate
y1 = 22.58 y dprob0.5 = 33.03 , y1.4 = 45.57
d−prime
Criterion beta
Amount of trials
Amount of sessions
SPL (dB re 20µPa)

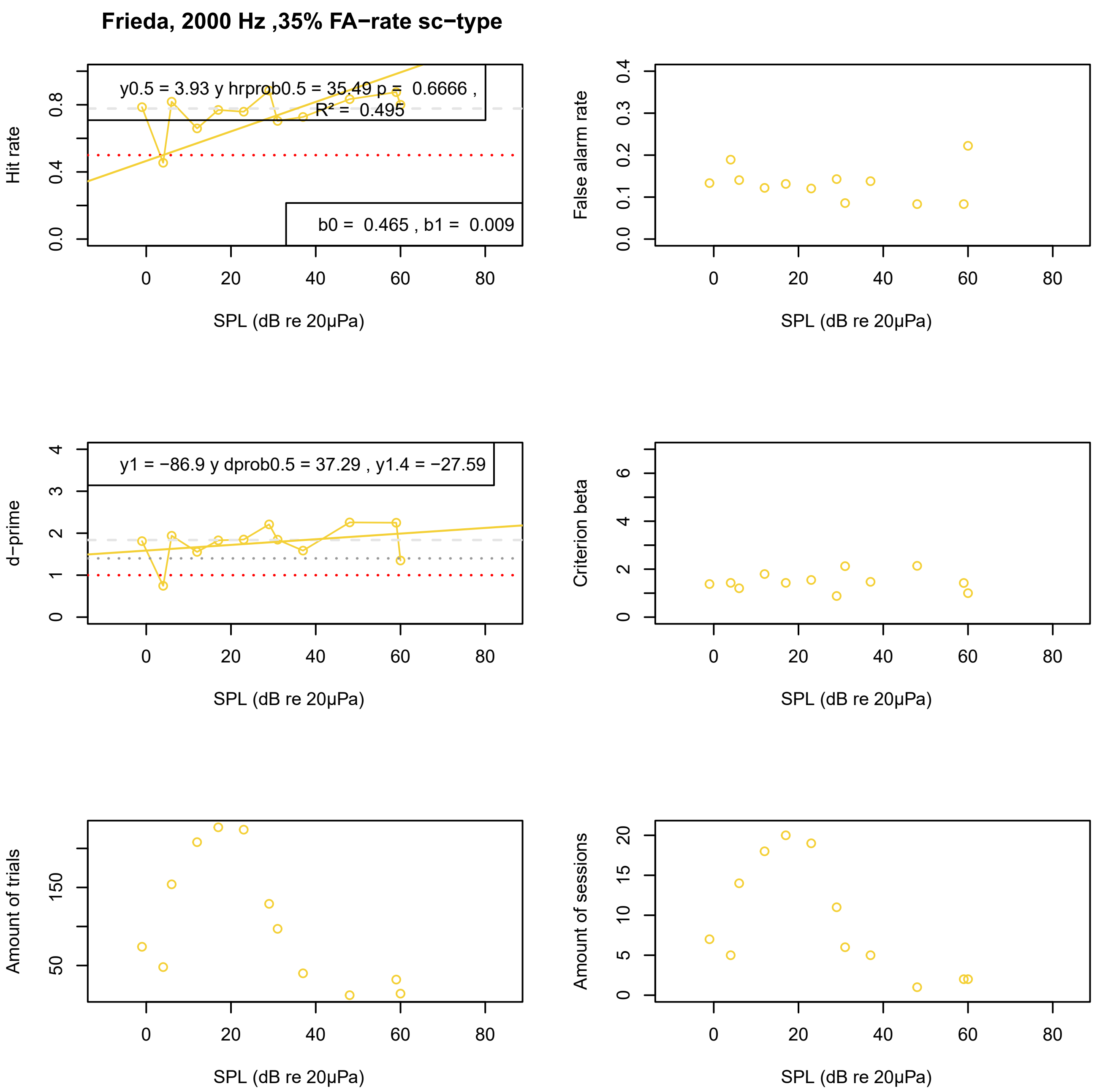

Frieda, 2000 Hz ,35% FA−rate sc−type
y0.5 = 3.93 y hrprob0.5 = 35.49 p = 0.6666 ,
R² = 0.495
b0 = 0.465 , b1 = 0.009
Hit rate
SPL (dB re 20µPa)
False alarm rate
SPL (dB re 20µPa)
y1 = −86.9 y dprob0.5 = 37.29 , y1.4 = −27.59
d−prime
SPL (dB re 20µPa)
Criterion beta
SPL (dB re 20µPa)
Amount of trials
SPL (dB re 20µPa)
Amount of sessions
SPL (dB re 20µPa)

Frieda, 4000 Hz ,35% FA−rate sc−type

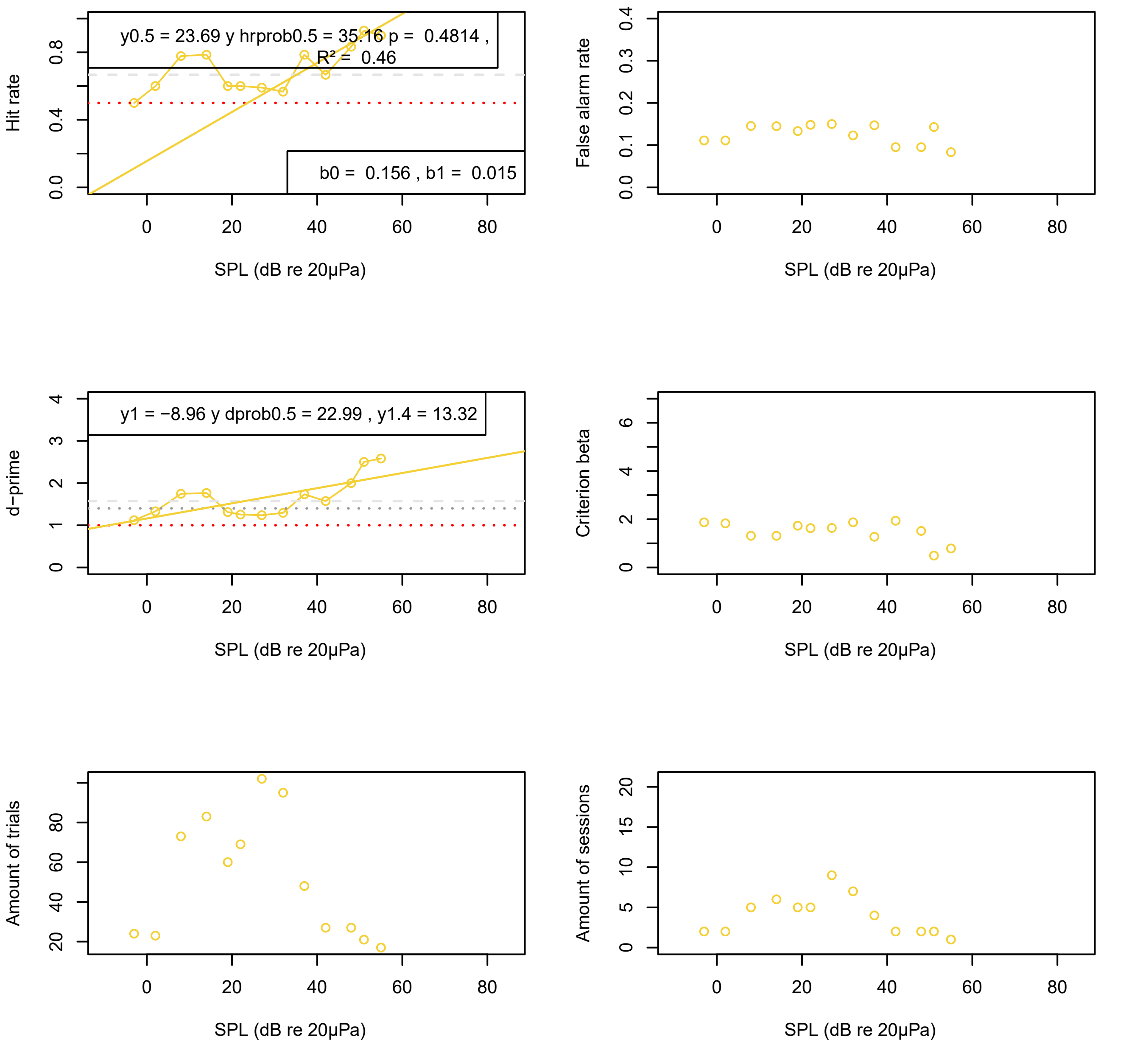

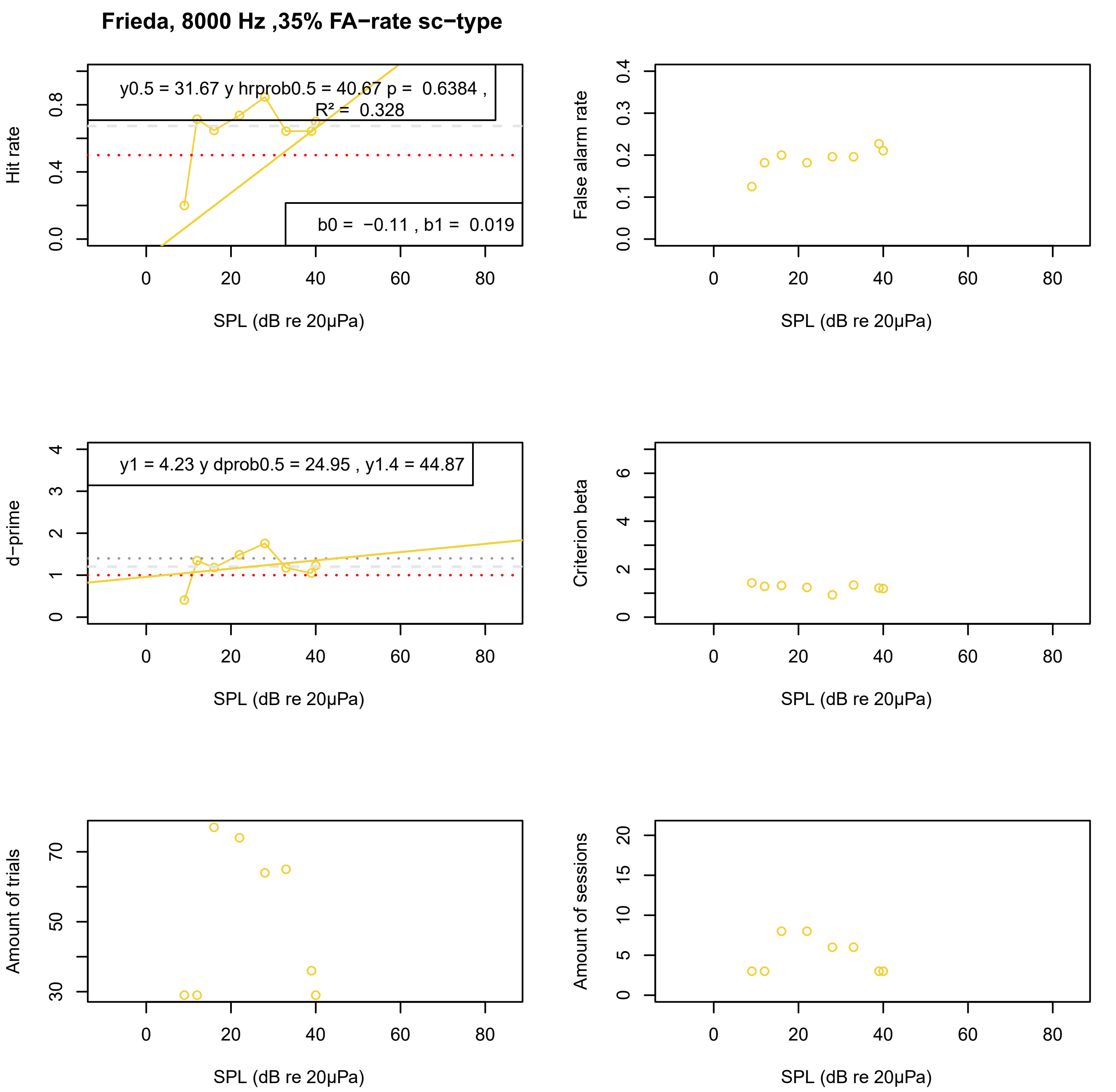
Frieda, 8000 Hz ,35% FA−rate sc−type
y0.5 = 31.67 y hrprob0.5 = 40.67 p = 0.6384 ,
R² = 0.328
b0 = −0.11 , b1 = 0.019
Hit rate
SPL (dB re 20µPa)
False alarm rate
y1 = 4.23 y dprob0.5 = 24.95 , y1.4 = 44.87
d−prime
Criterion beta
Amount of trials
Amount of sessions

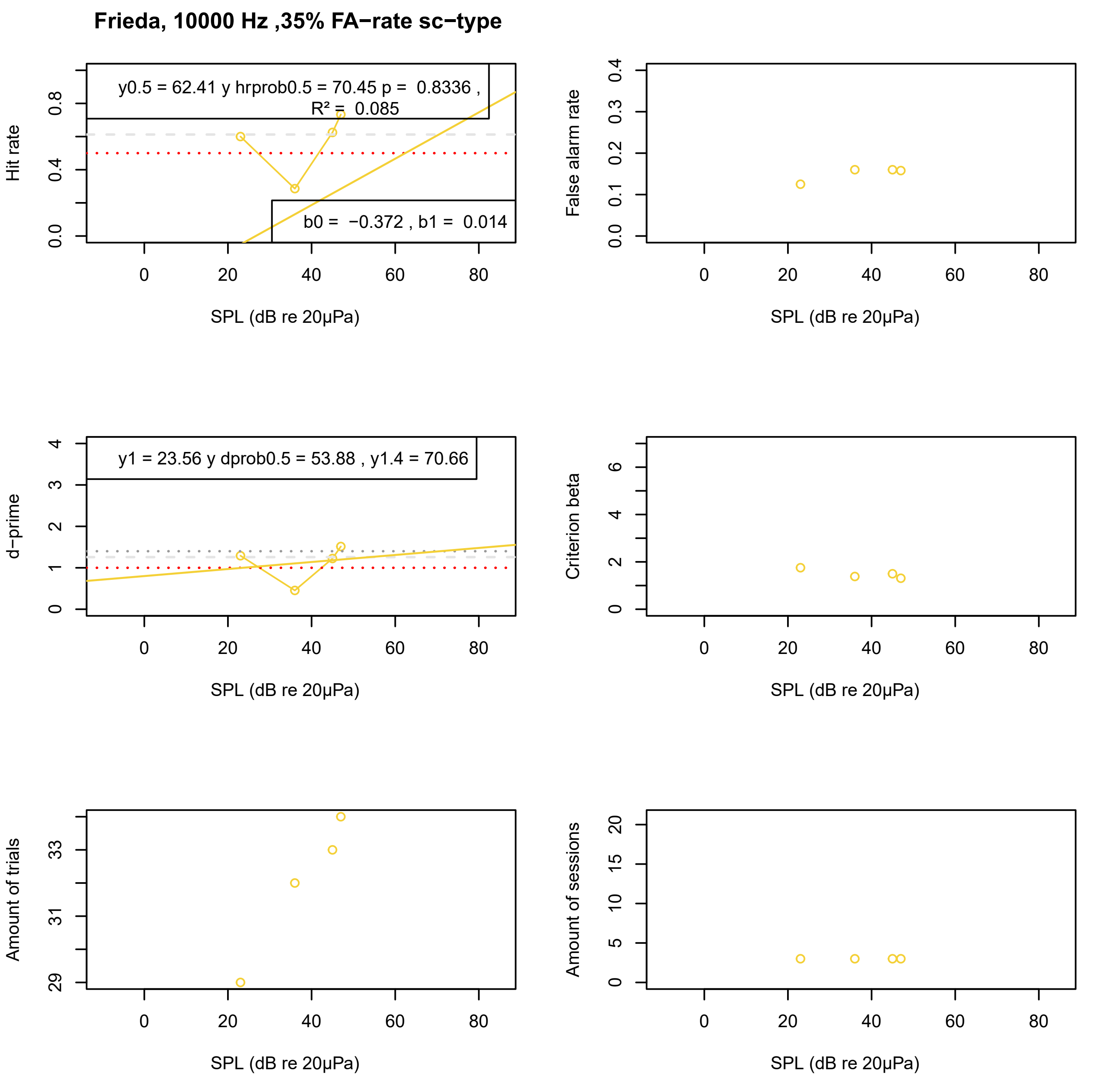

Frieda, 10000 Hz ,35% FA−rate sc−type
y0.5 = 62.41 y hrprob0.5 = 70.45 p = 0.8336 , R² = 0.085
b0 = −0.372 , b1 = 0.014
Hit rate
False alarm rate
y1 = 23.56 y dprob0.5 = 53.88 , y1.4 = 70.66
d−prime
Criterion beta
Amount of trials
Amount of sessions
SPL (dB re 20µPa)

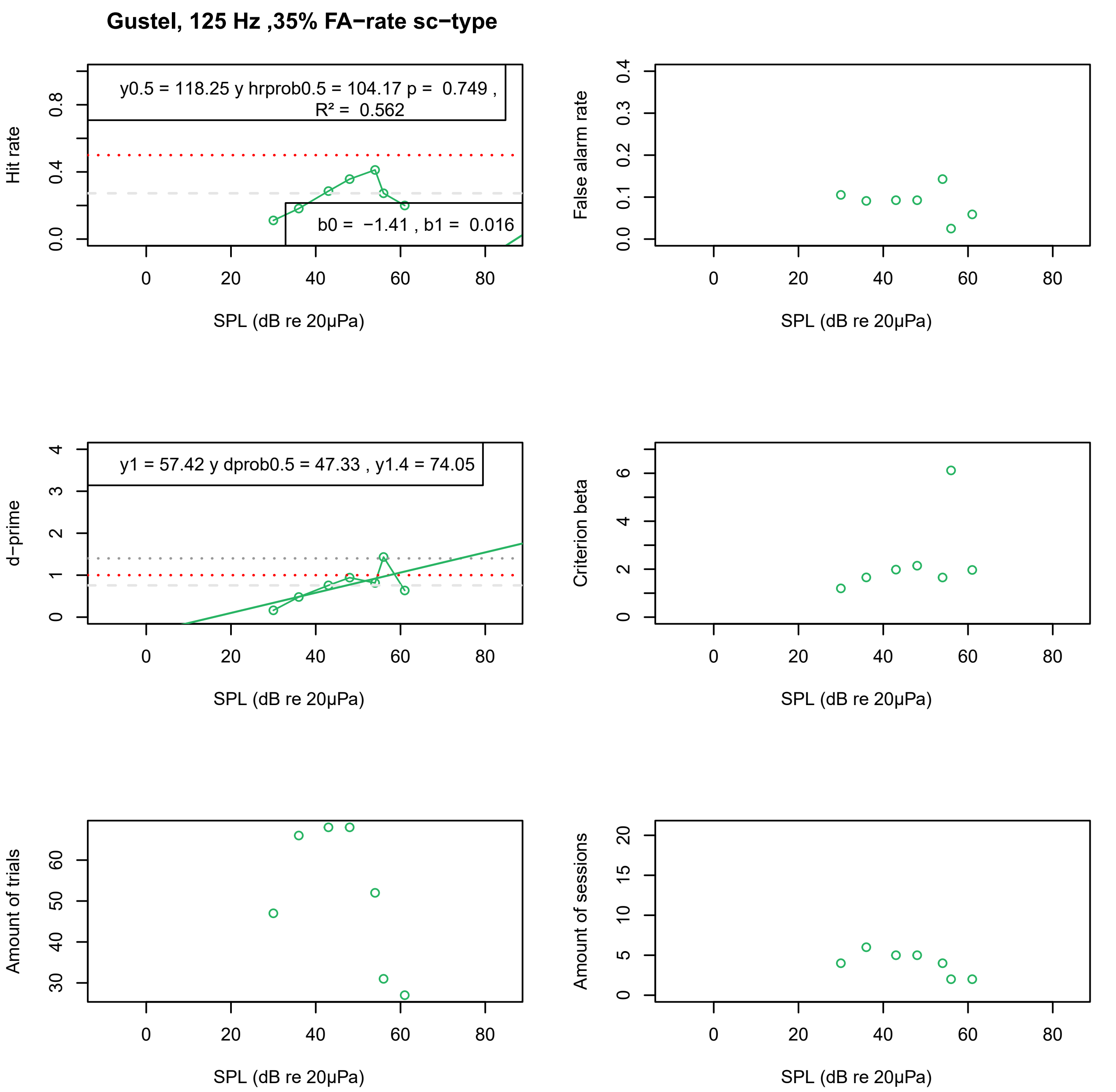
Gustel, 125 Hz ,35% FA−rate sc−type
y0.5 = 118.25 y hrprob0.5 = 104.17 p = 0.749 , R² = 0.562
b0 = −1.41 , b1 = 0.016
Hit rate
False alarm rate
y1 = 57.42 y dprob0.5 = 47.33 , y1.4 = 74.05
d−prime
Criterion beta
Amount of trials
Amount of sessions
SPL (dB re 20µPa)

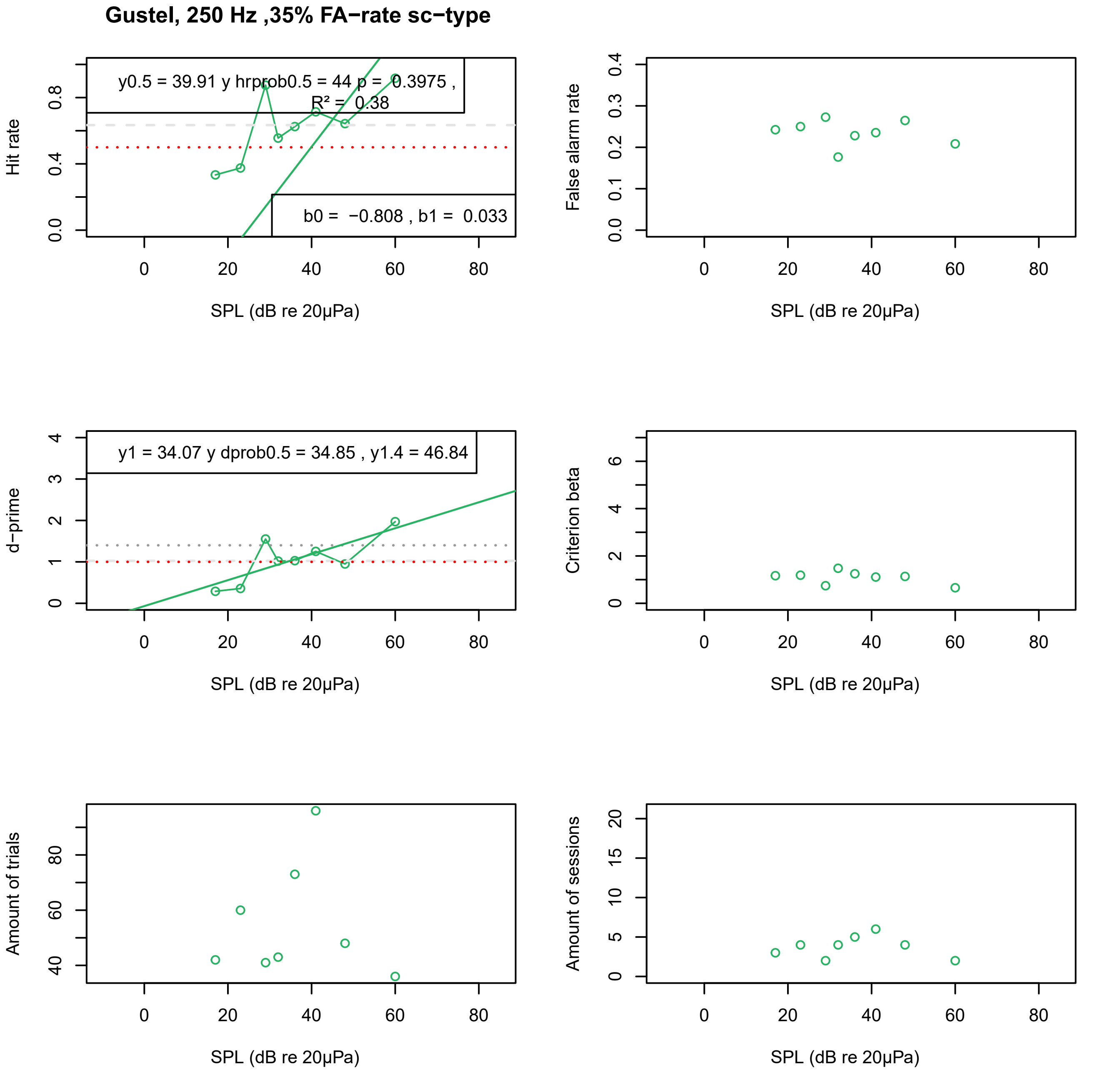

Gustel, 250 Hz ,35% FA−rate sc−type
y0.5 = 39.91 y hrprob0.5 = 44 p = 0.3975 ,
R² = 0.38
b0 = −0.808 , b1 = 0.033
Hit rate
False alarm rate
y1 = 34.07 y dprob0.5 = 34.85 , y1.4 = 46.84
d−prime
Criterion beta
Amount of trials
Amount of sessions
SPL (dB re 20µPa)

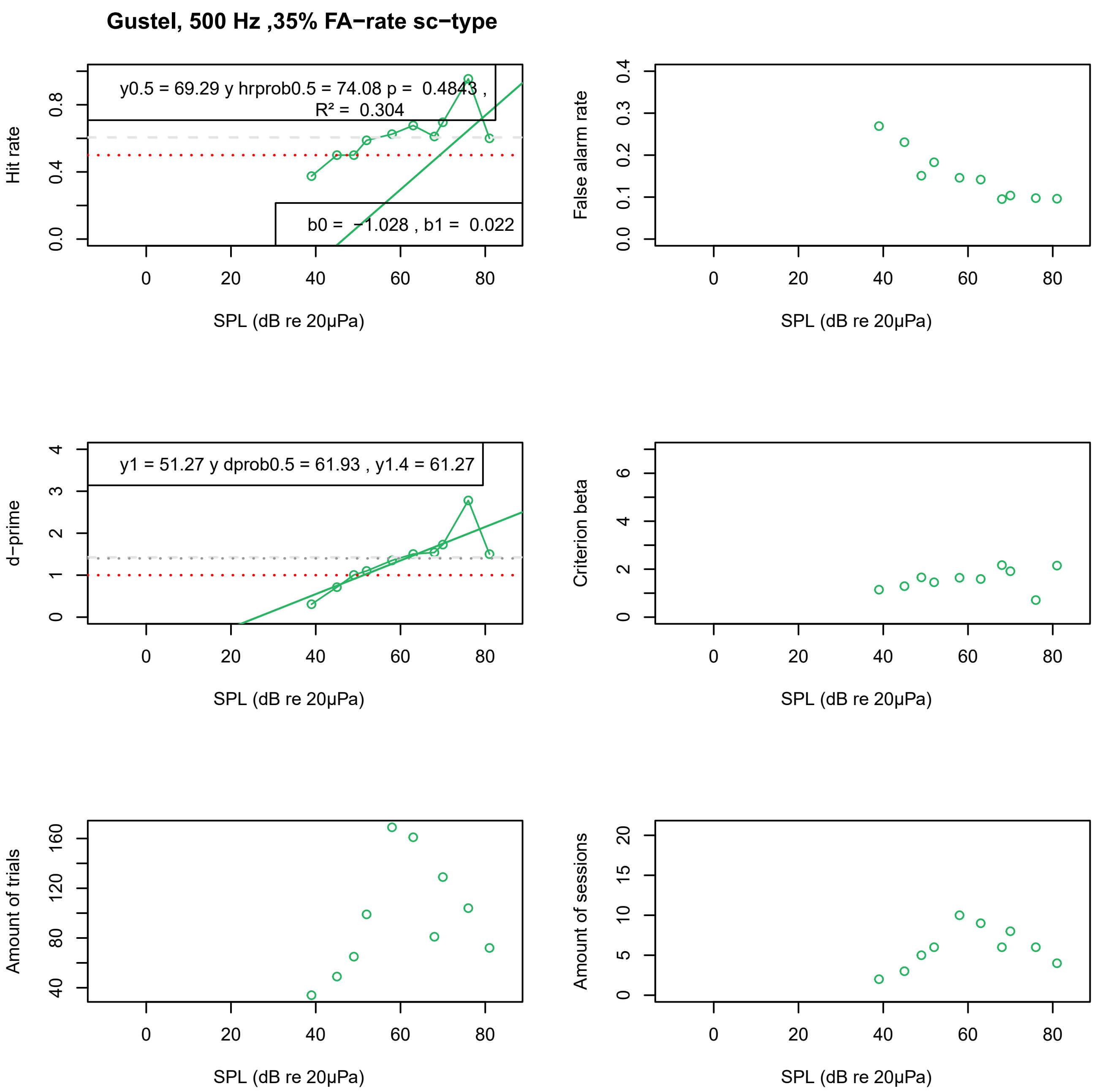

Gustel, 500 Hz ,35% FA−rate sc−type
y0.5 = 69.29 y hrprob0.5 = 74.08 p = 0.4843 , R² = 0.304
b0 = −1.028 , b1 = 0.022
Hit rate
False alarm rate
y1 = 51.27 y dprob0.5 = 61.93 , y1.4 = 61.27
d−prime
Criterion beta
Amount of trials
Amount of sessions
SPL (dB re 20µPa)

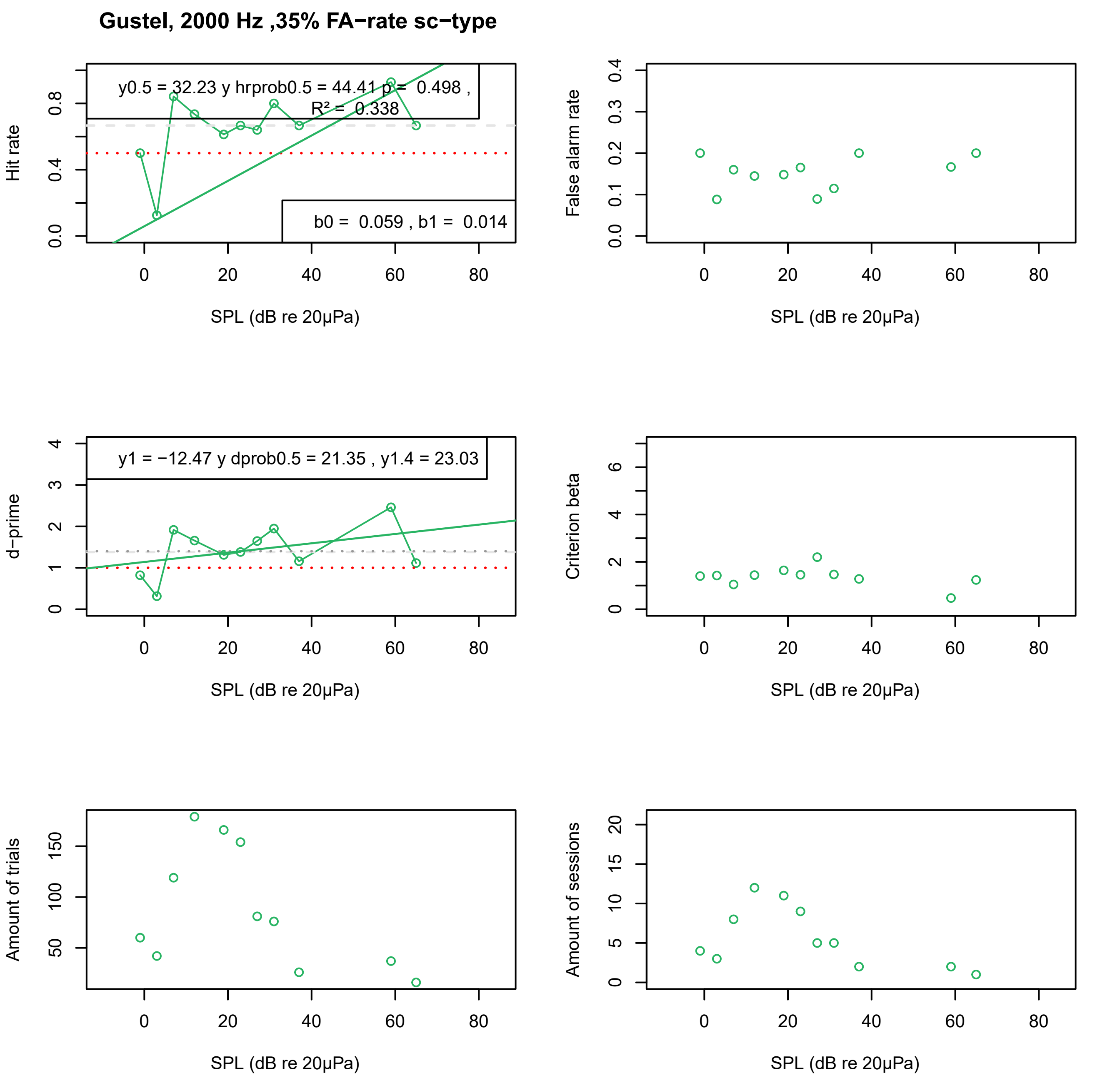

Gustel, 2000 Hz ,35% FA−rate sc−type
y0.5 = 32.23 y hrprob0.5 = 44.41 p = 0.498 ,
R² = 0.338
b0 = 0.059 , b1 = 0.014
Hit rate
False alarm rate
y1 = −12.47 y dprob0.5 = 21.35 , y1.4 = 23.03
d−prime
Criterion beta
Amount of trials
Amount of sessions
SPL (dB re 20μPa)

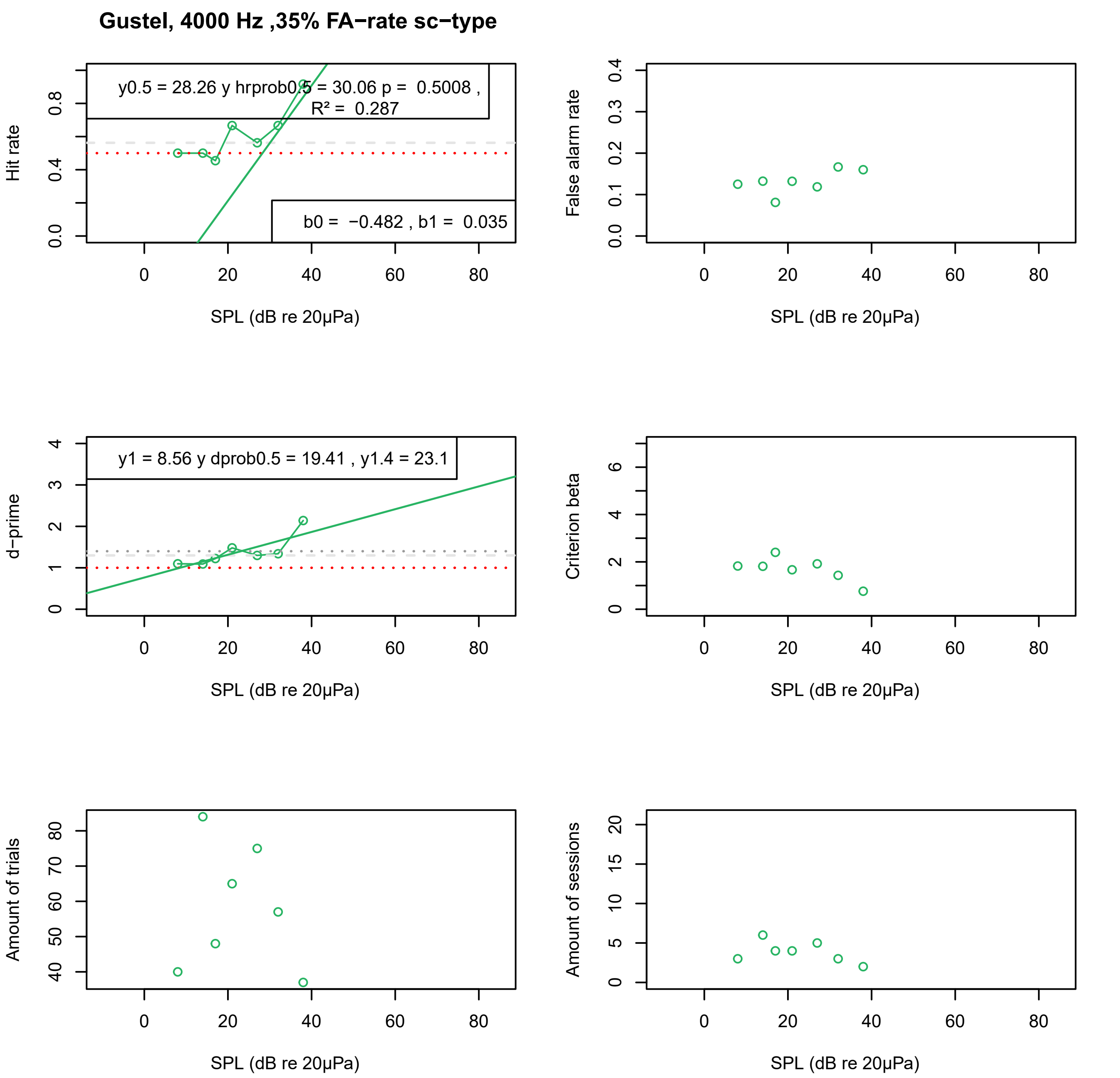
Gustel, 4000 Hz ,35% FA−rate sc−type
y0.5 = 28.26 y hrprob0.5 = 30.06 p = 0.5008 , R² = 0.287
b0 = −0.482 , b1 = 0.035
Hit rate
False alarm rate
y1 = 8.56 y dprob0.5 = 19.41 , y1.4 = 23.1
d−prime
Criterion beta
Amount of trials
Amount of sessions
SPL (dB re 20µPa)

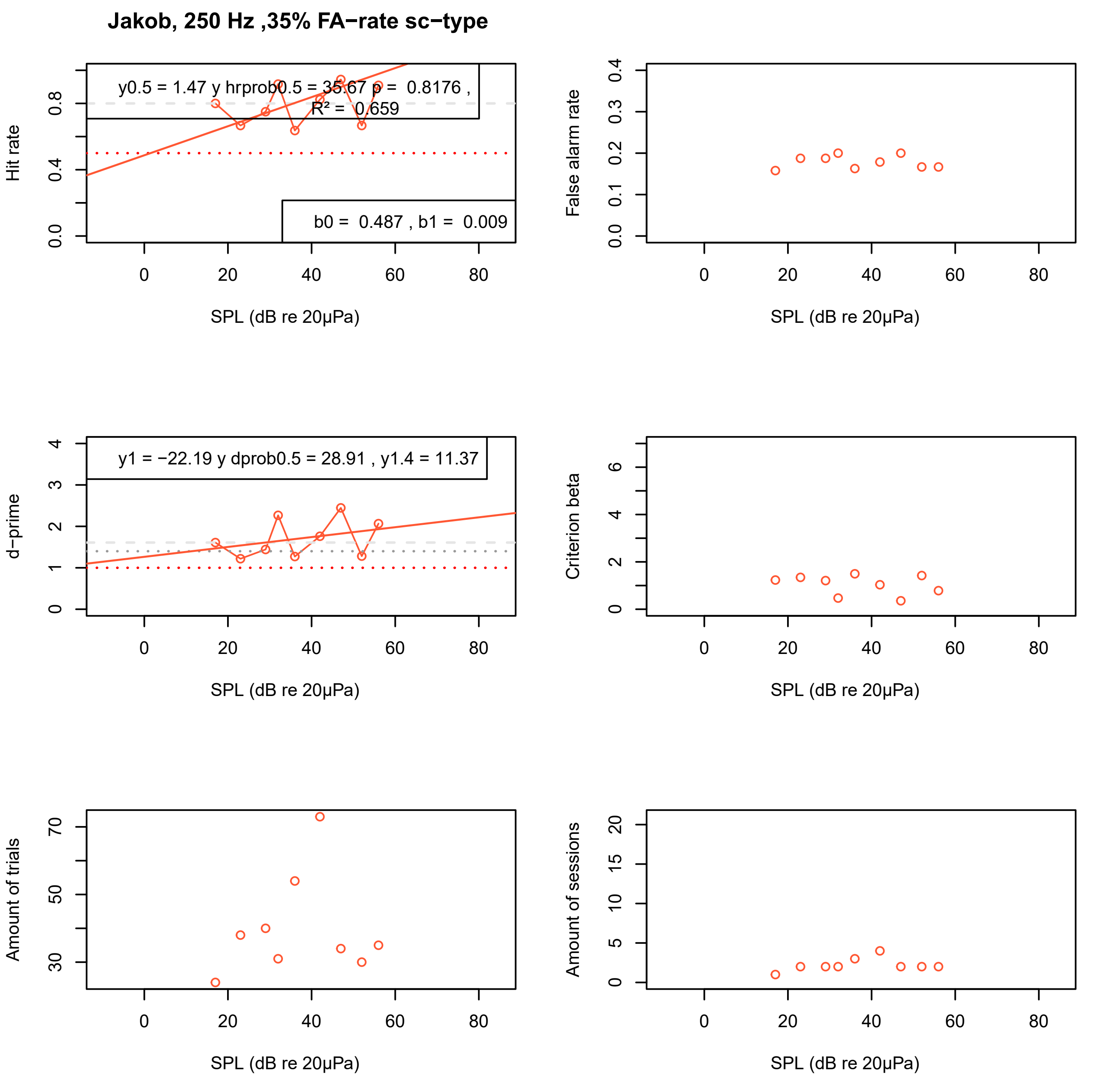
Jakob, 250 Hz ,35% FA−rate sc−type
y0.5 = 1.47 y hrprob0.5 = 35.67 p = 0.8176 ,
R² = 0.659
b0 = 0.487 , b1 = 0.009
Hit rate
False alarm rate
y1 = −22.19 y dprob0.5 = 28.91 , y1.4 = 11.37
d−prime
Criterion beta
Amount of trials
Amount of sessions
SPL (dB re 20μPa)

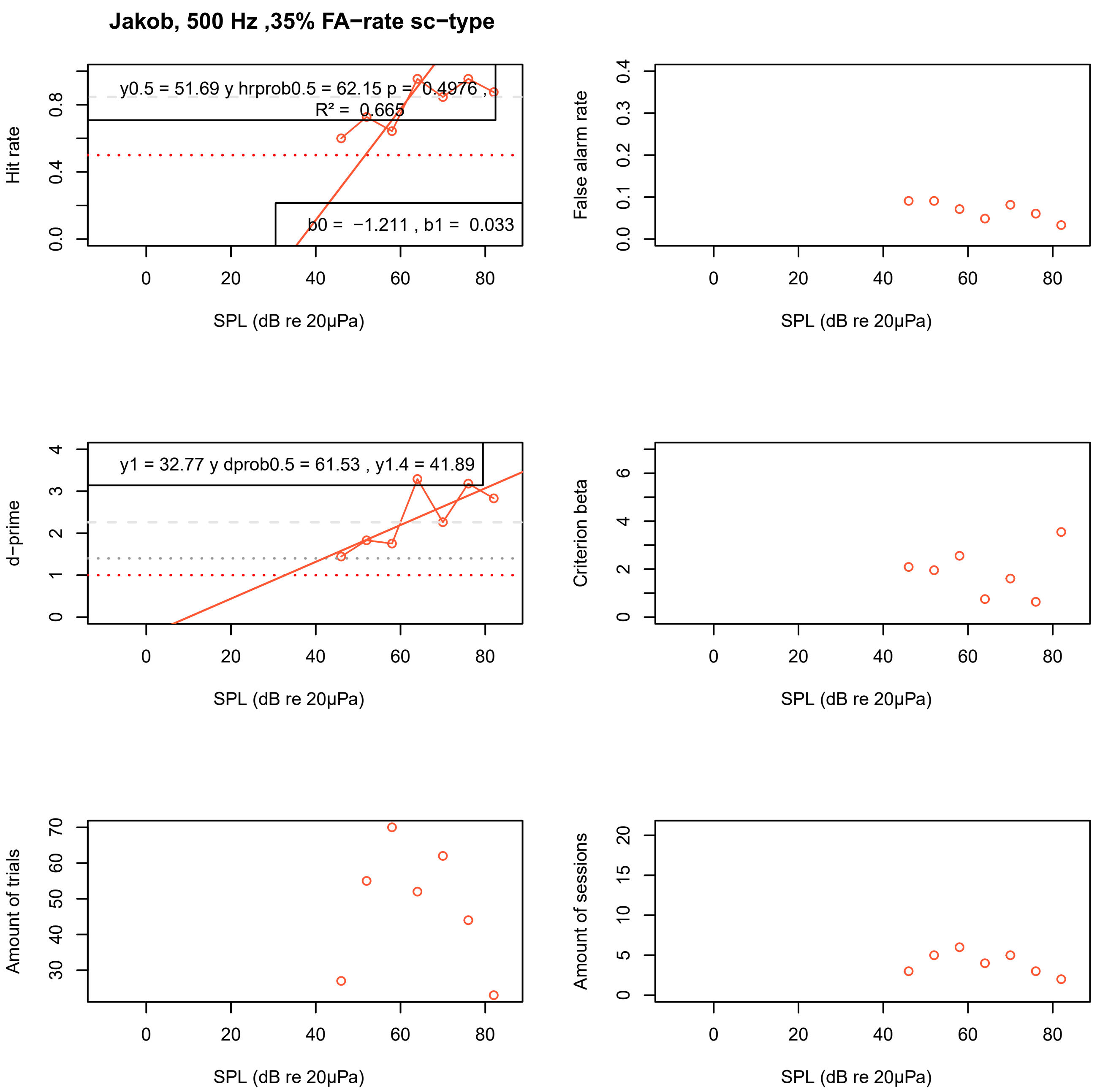

Jakob, 500 Hz ,35% FA−rate sc−type
y0.5 = 51.69 y hrprob0.5 = 62.15 p = 0.4976 ,
R² = 0.665
b0 = −1.211 , b1 = 0.033
Hit rate
False alarm rate
y1 = 32.77 y dprob0.5 = 61.53 , y1.4 = 41.89
d−prime
Criterion beta
Amount of trials
Amount of sessions
SPL (dB re 20μPa)

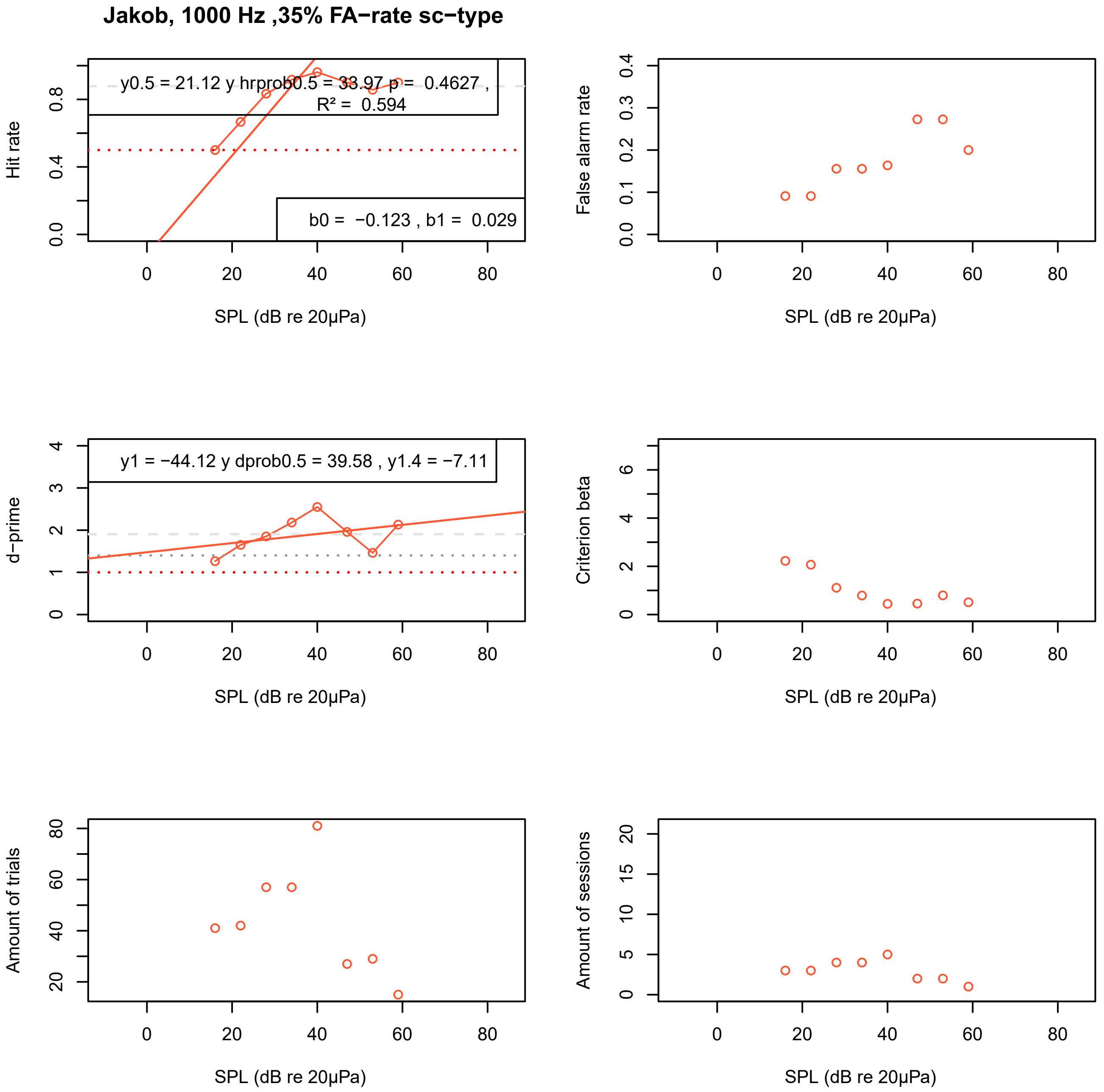

Jakob, 1000 Hz ,35% FA−rate sc−type
y0.5 = 21.12 y hrprob0.5 = 33.97 p = 0.4627 ,
R² = 0.594
b0 = −0.123 , b1 = 0.029
Hit rate
SPL (dB re 20µPa)
False alarm rate
SPL (dB re 20µPa)
y1 = −44.12 y dprob0.5 = 39.58 , y1.4 = −7.11
d−prime
SPL (dB re 20µPa)
Criterion beta
SPL (dB re 20µPa)
Amount of trials
SPL (dB re 20µPa)
Amount of sessions
SPL (dB re 20µPa)

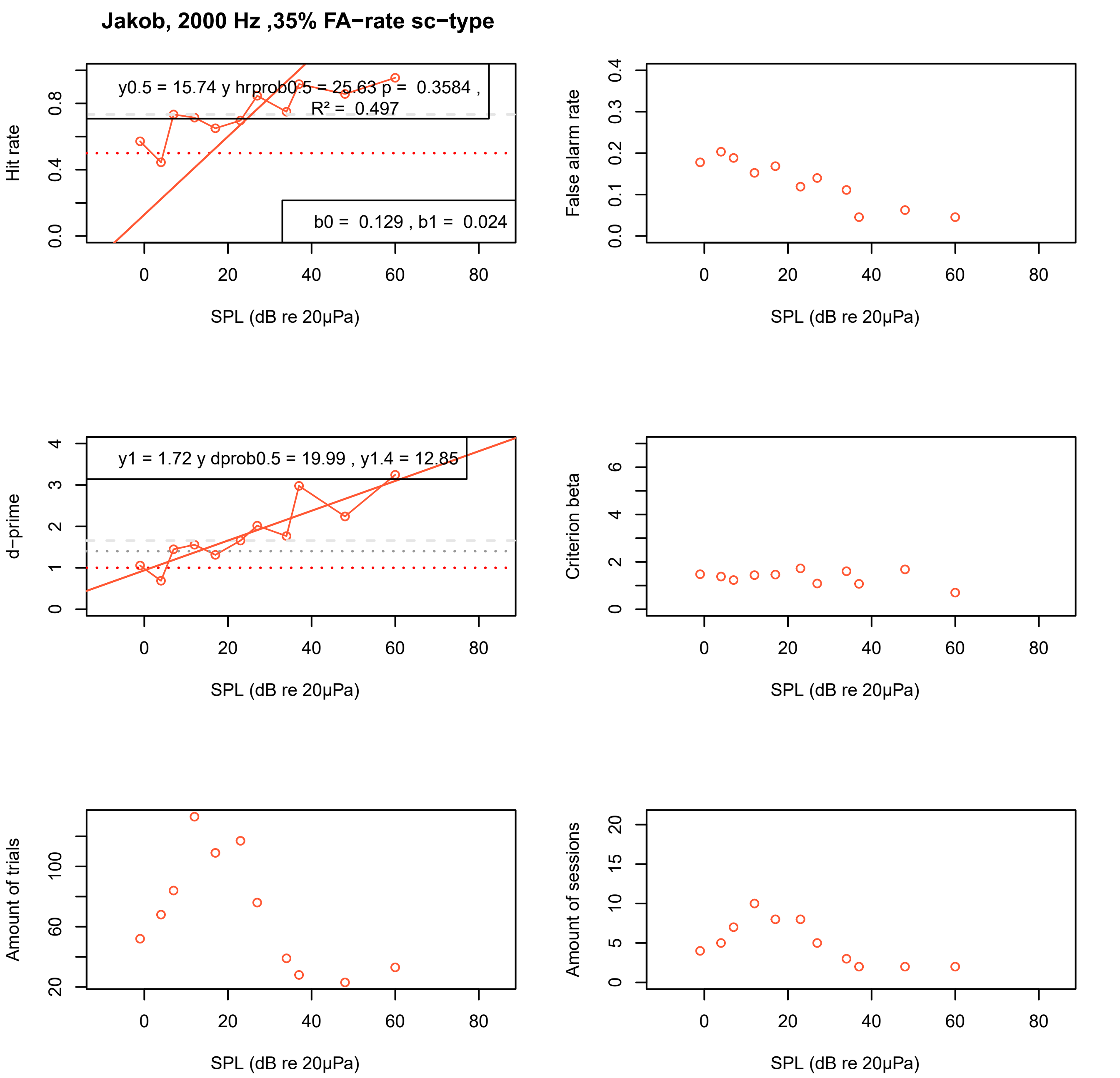
Jakob, 2000 Hz ,35% FA−rate sc−type
y0.5 = 15.74 y hrprob0.5 = 25.63 p = 0.3584 ,
R² = 0.497
b0 = 0.129 , b1 = 0.024
Hit rate
False alarm rate
y1 = 1.72 y dprob0.5 = 19.99 , y1.4 = 12.85
d−prime
Criterion beta
Amount of trials
Amount of sessions
SPL (dB re 20µPa)

Jakob, 4000 Hz ,35% FA−rate sc−type

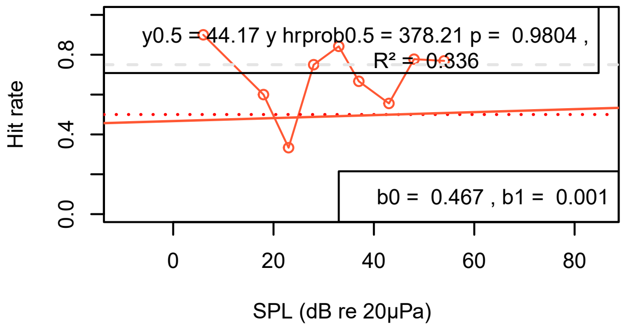

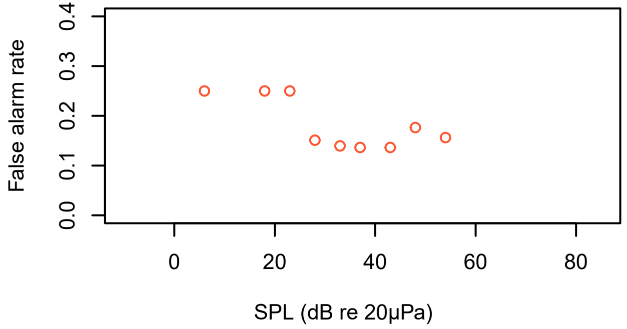

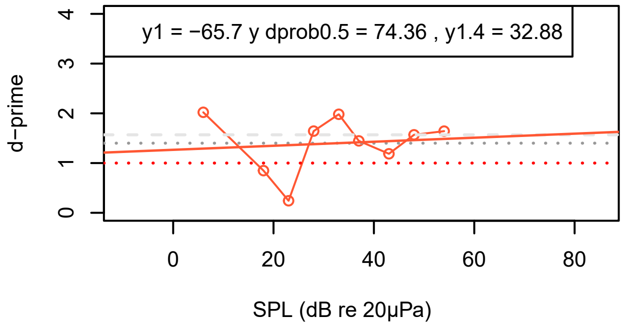

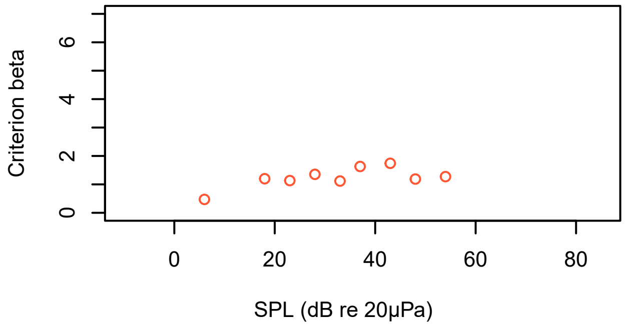

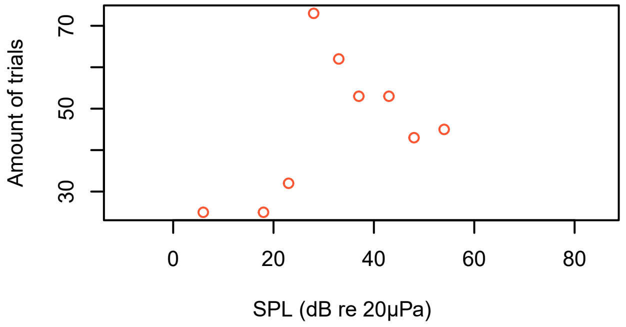

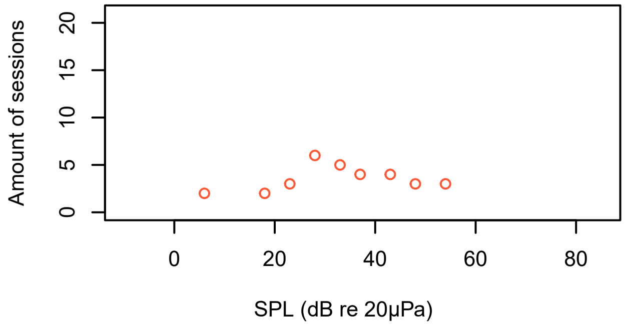

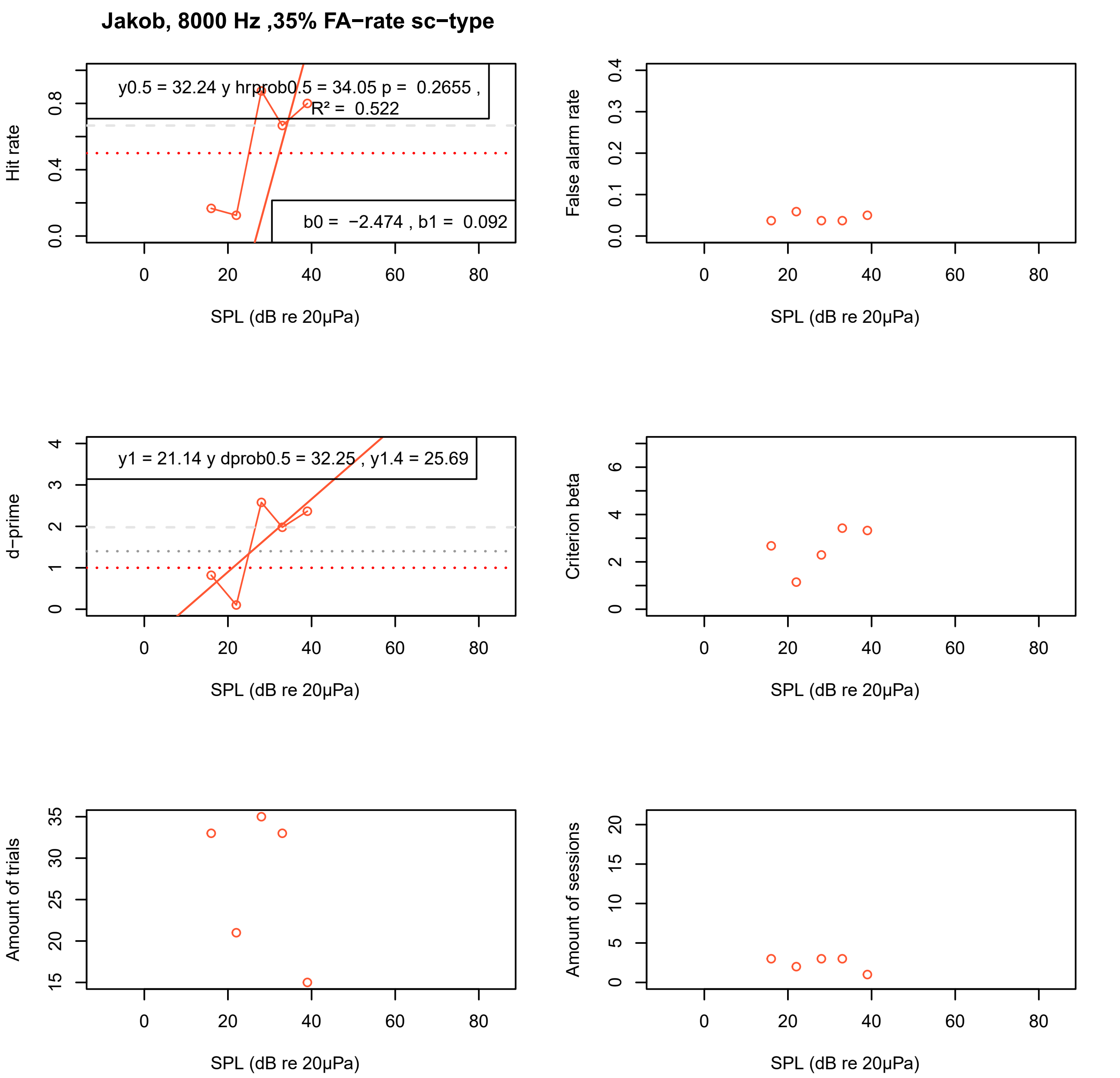

Jakob, 8000 Hz ,35% FA−rate sc−type
y0.5 = 32.24 y hrprob0.5 = 34.05 p = 0.2655 , R² = 0.522
b0 = −2.474 , b1 = 0.092
Hit rate
False alarm rate
y1 = 21.14 y dprob0.5 = 32.25 , y1.4 = 25.69
d−prime
Criterion beta
Amount of trials
Amount of sessions
SPL (dB re 20µPa)

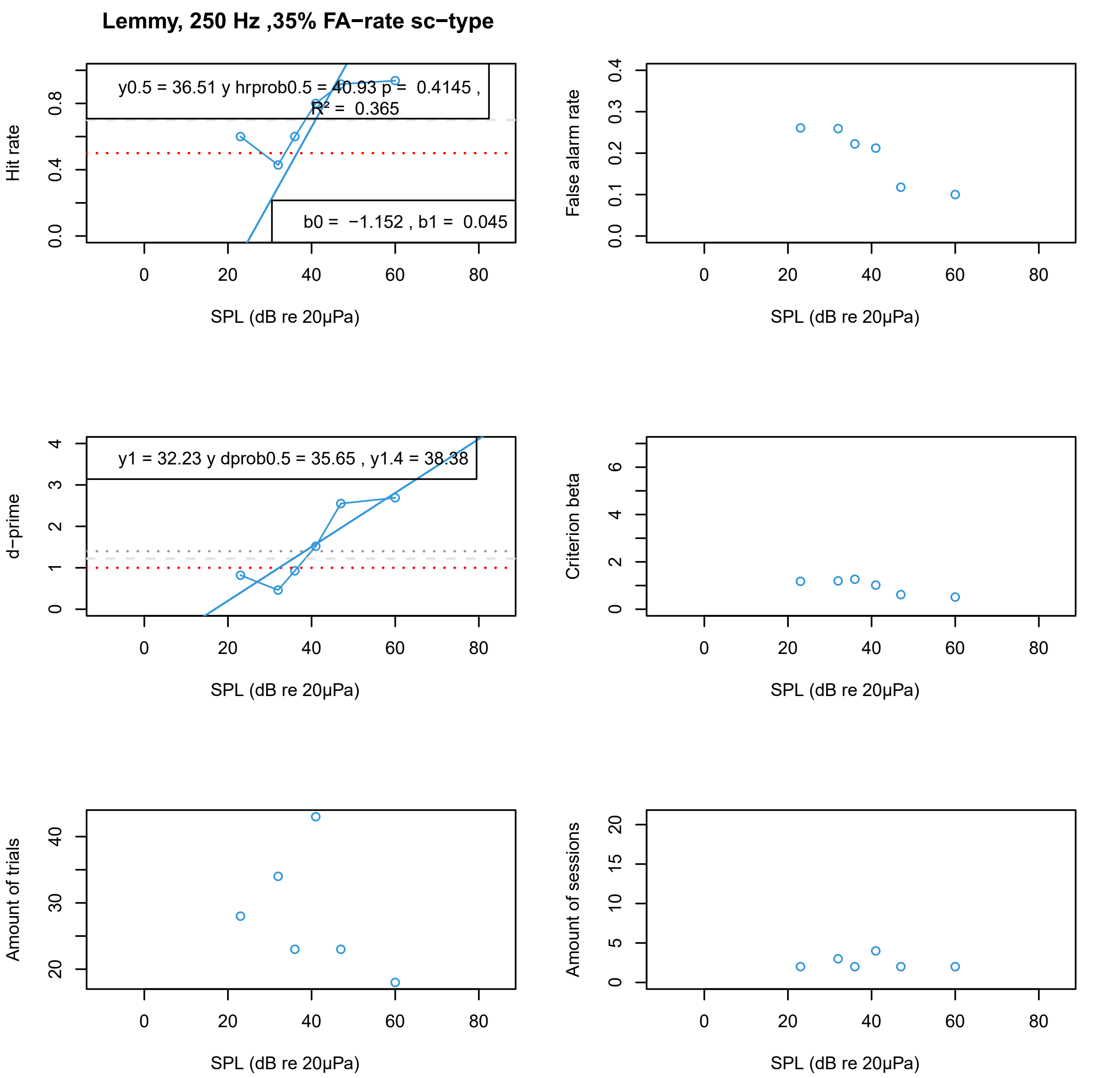

Lemmy, 250 Hz ,35% FA−rate sc−type
y0.5 = 36.51 y hrprob0.5 = 40.93 p = 0.4145 ,
R² = 0.365
b0 = −1.152 , b1 = 0.045
Hit rate
False alarm rate
y1 = 32.23 y dprob0.5 = 35.65 , y1.4 = 38.38
d−prime
Criterion beta
Amount of trials
Amount of sessions
SPL (dB re 20µPa)

Lemmy, 500 Hz ,35% FA−rate sc−type

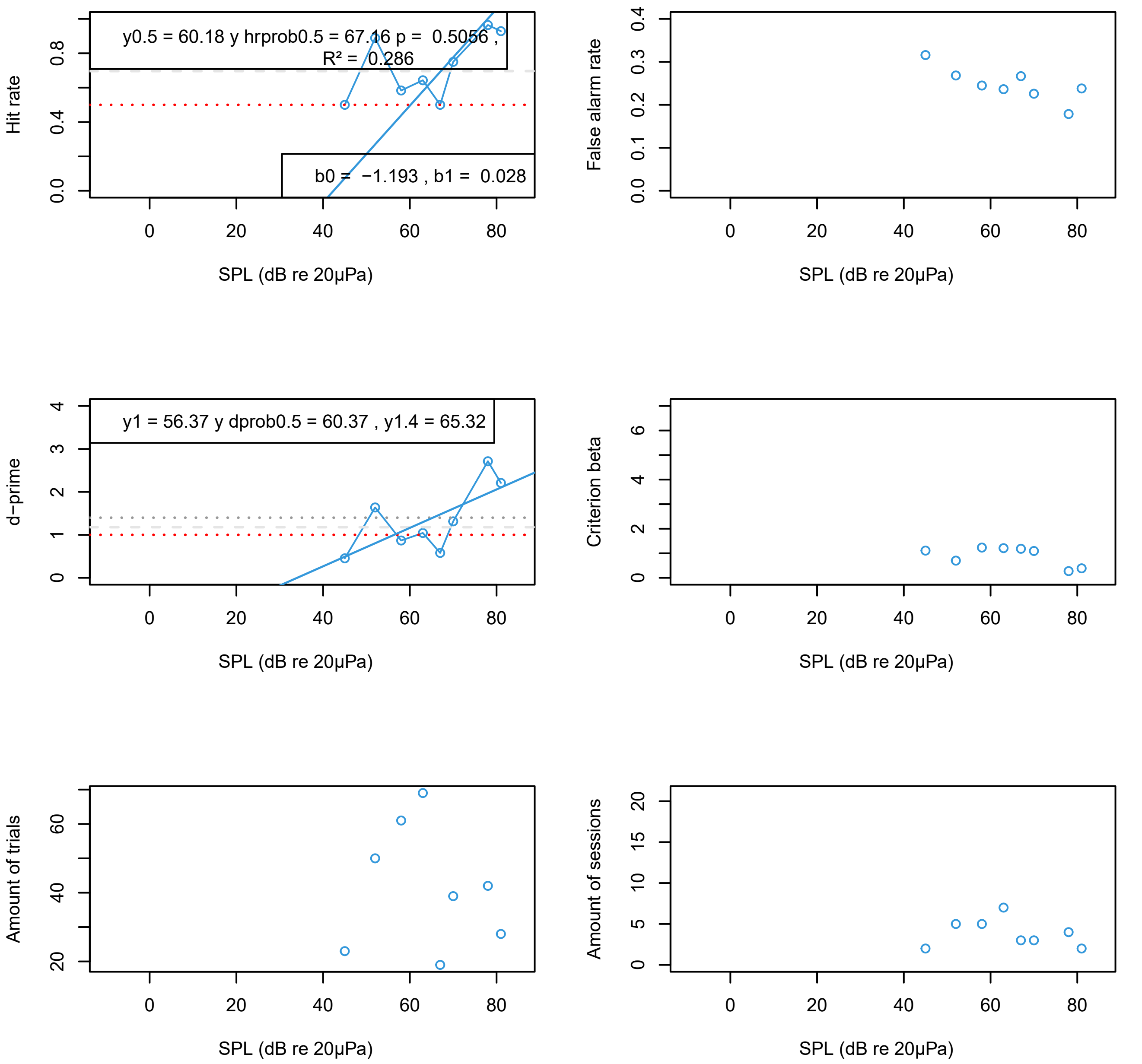

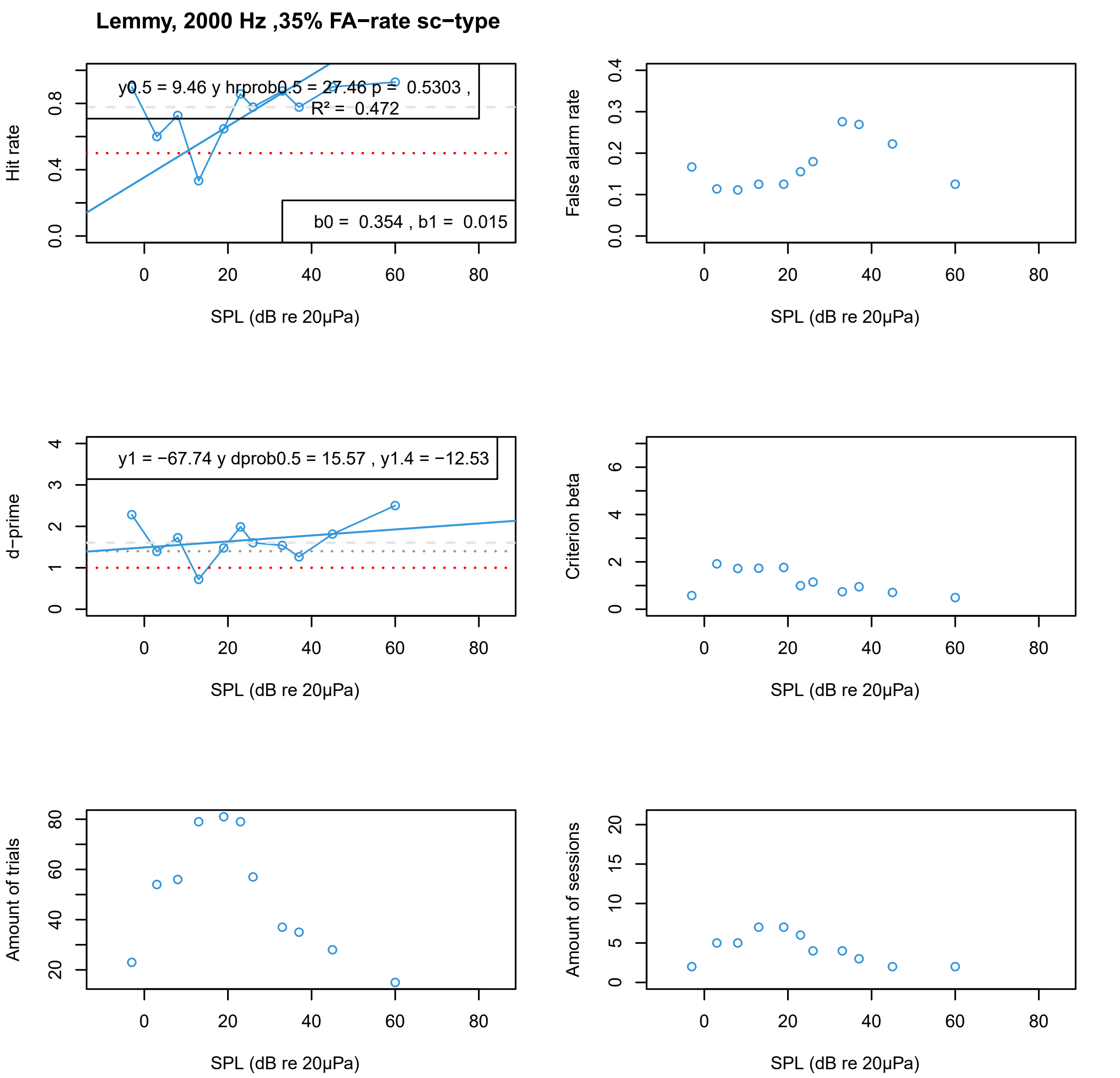

Lemmy, 2000 Hz ,35% FA−rate sc−type
y0.5 = 9.46 y hrprob0.5 = 27.46 p = 0.5303 ,
R² = 0.472
b0 = 0.354 , b1 = 0.015
Hit rate
False alarm rate
y1 = −67.74 y dprob0.5 = 15.57 , y1.4 = −12.53
d−prime
Criterion beta
Amount of trials
Amount of sessions
SPL (dB re 20µPa)

Lemmy, 4000 Hz ,35% FA−rate sc−type

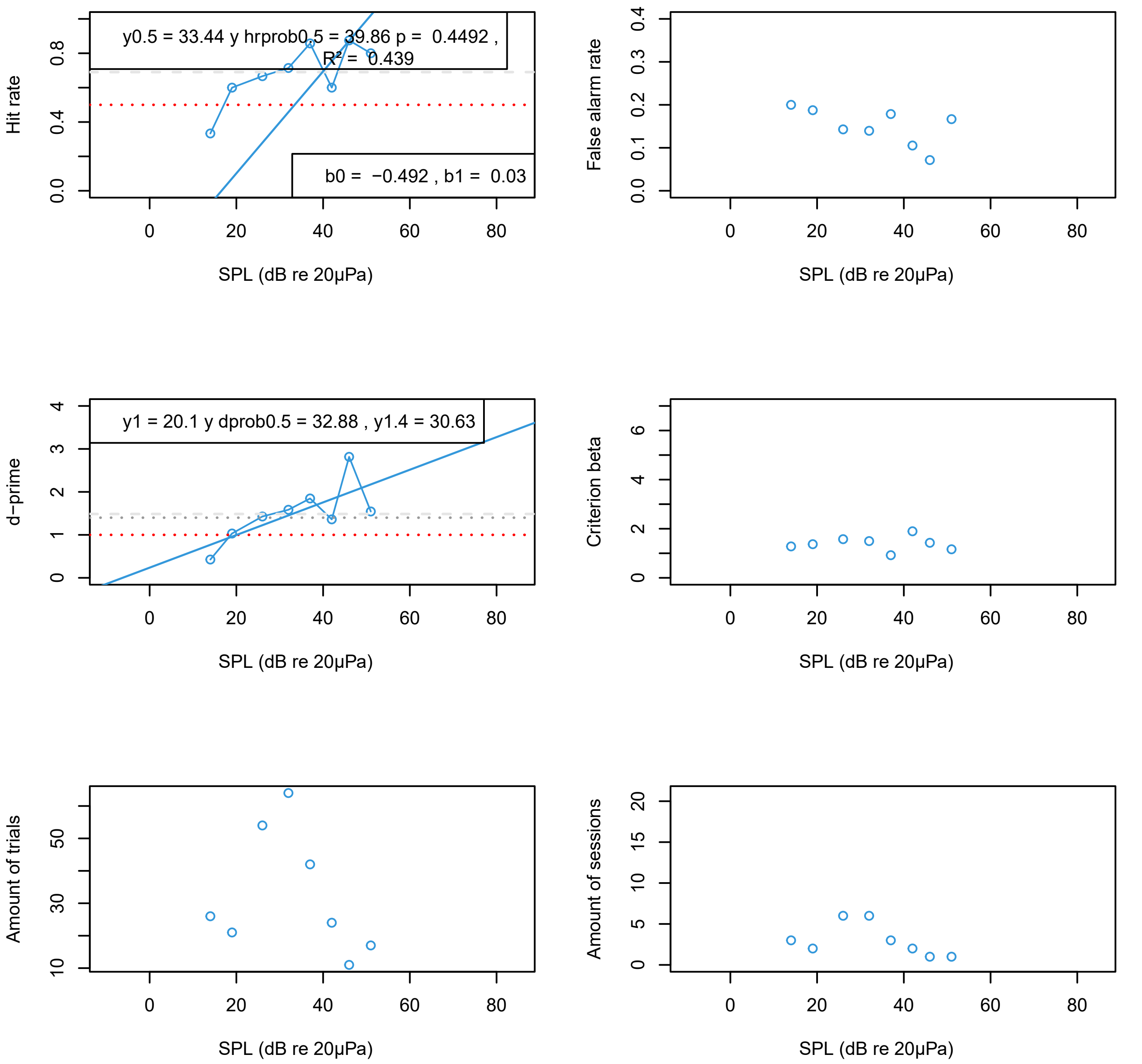

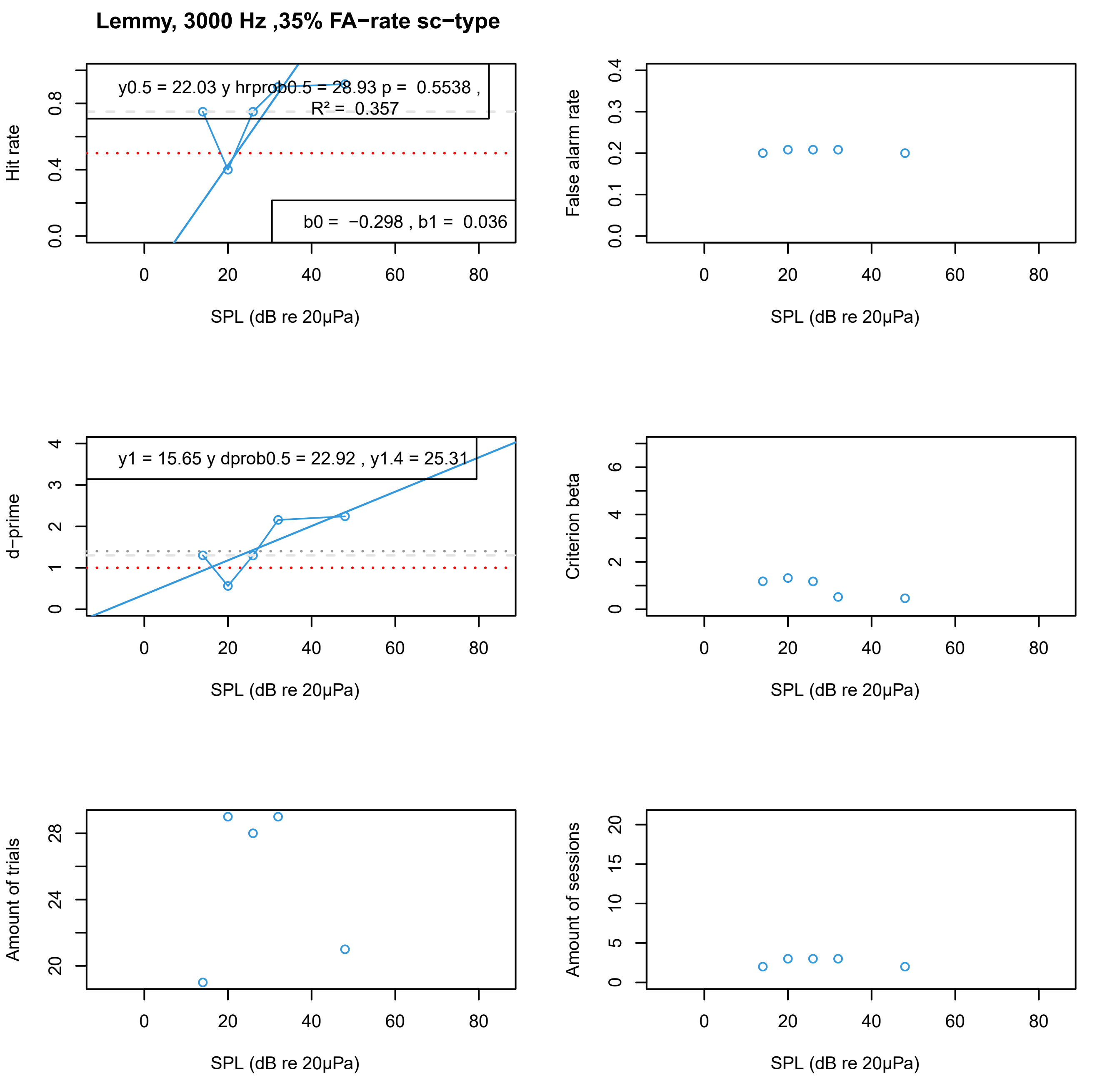
Lemmy, 3000 Hz ,35% FA−rate sc−type
y0.5 = 22.03 y hrprob0.5 = 28.93 p = 0.5538 ,
R² = 0.357
b0 = −0.298 , b1 = 0.036
Hit rate
False alarm rate
y1 = 15.65 y dprob0.5 = 22.92 , y1.4 = 25.31
d−prime
Criterion beta
Amount of trials
Amount of sessions
SPL (dB re 20μPa)
