# Supplementary material for: Biological relevance and methodological implications of unexpected hearing thresholds in a diving bird
Source: Sci Rep. 2024 Dec 23;14:30592. doi: 10.1038/s41598-024-82942-2 (PMC11666583; doi:10.1038/s41598-024-82942-2)
Supplement: Supplementary file 8 — Supplementary Material 8 [file 41598_2024_82942_MOESM8_ESM.docx]

**SUPPLEMENTARY FIGURES**


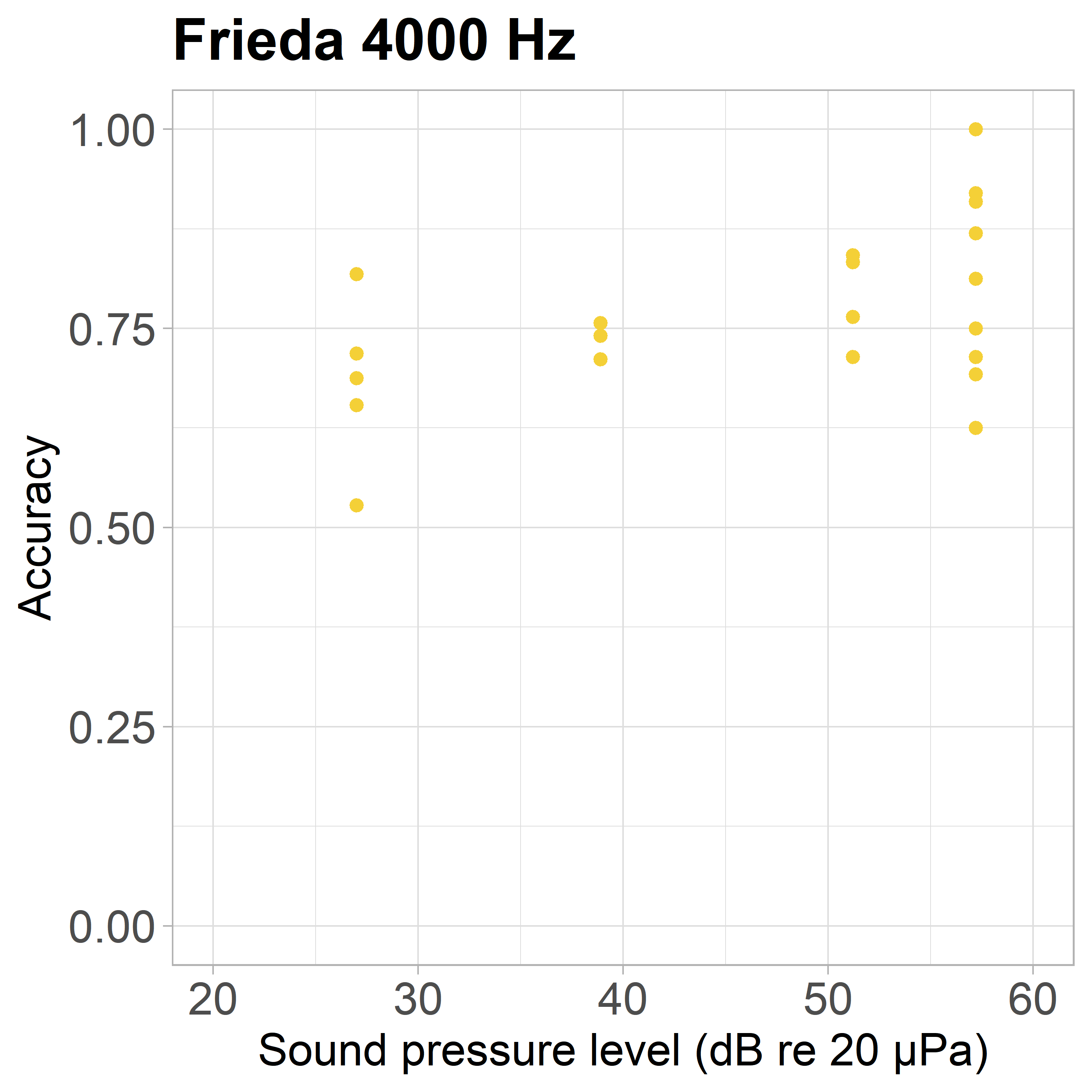

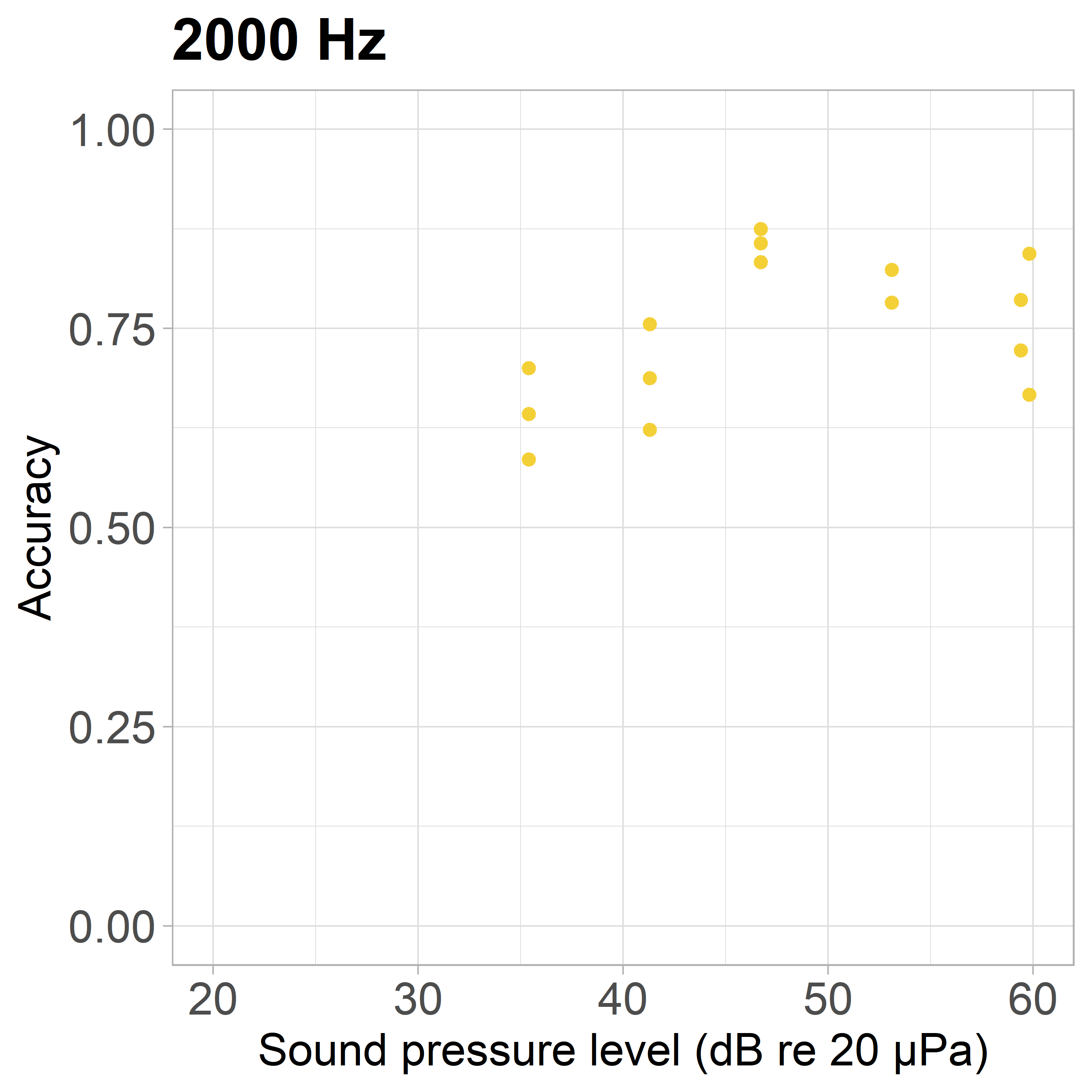


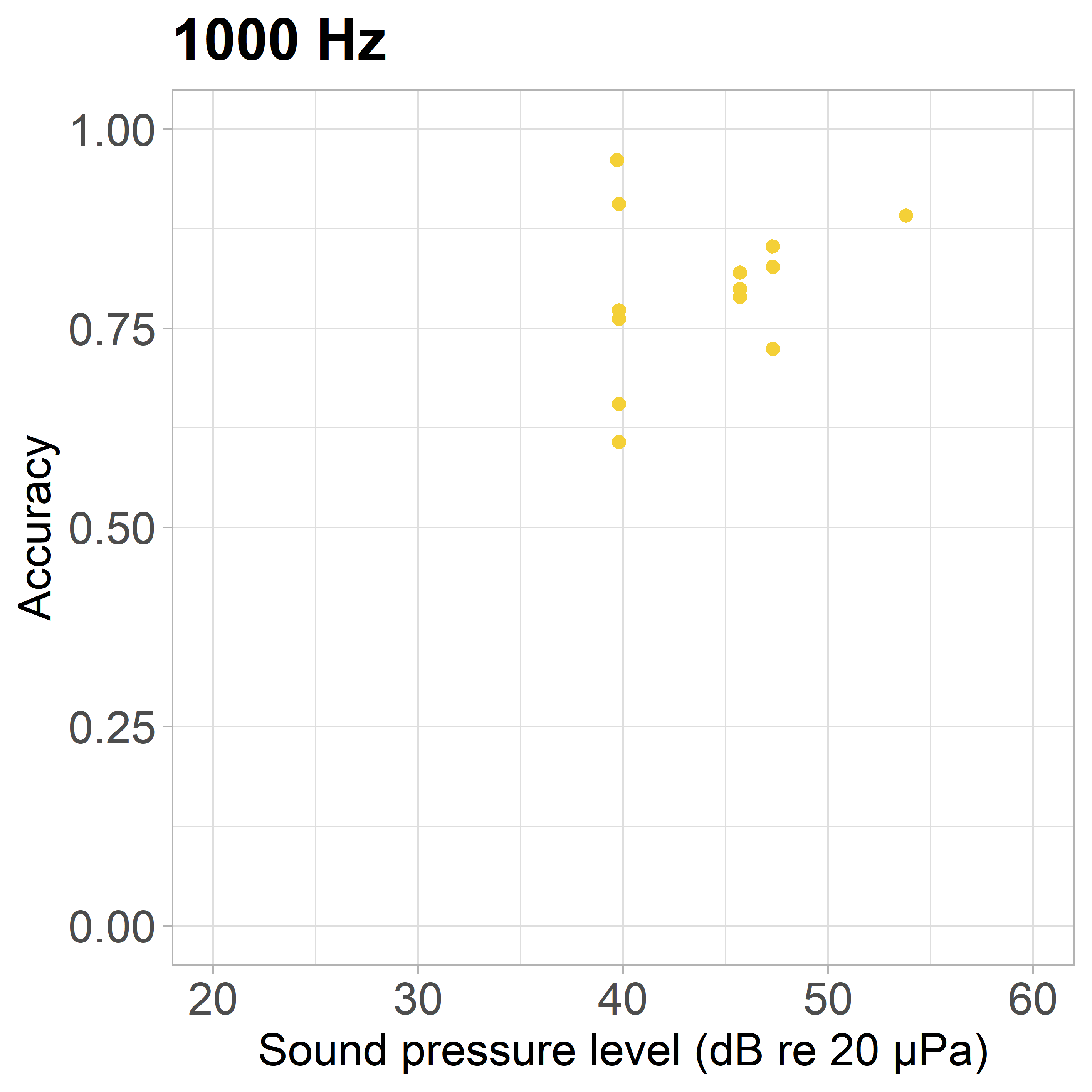

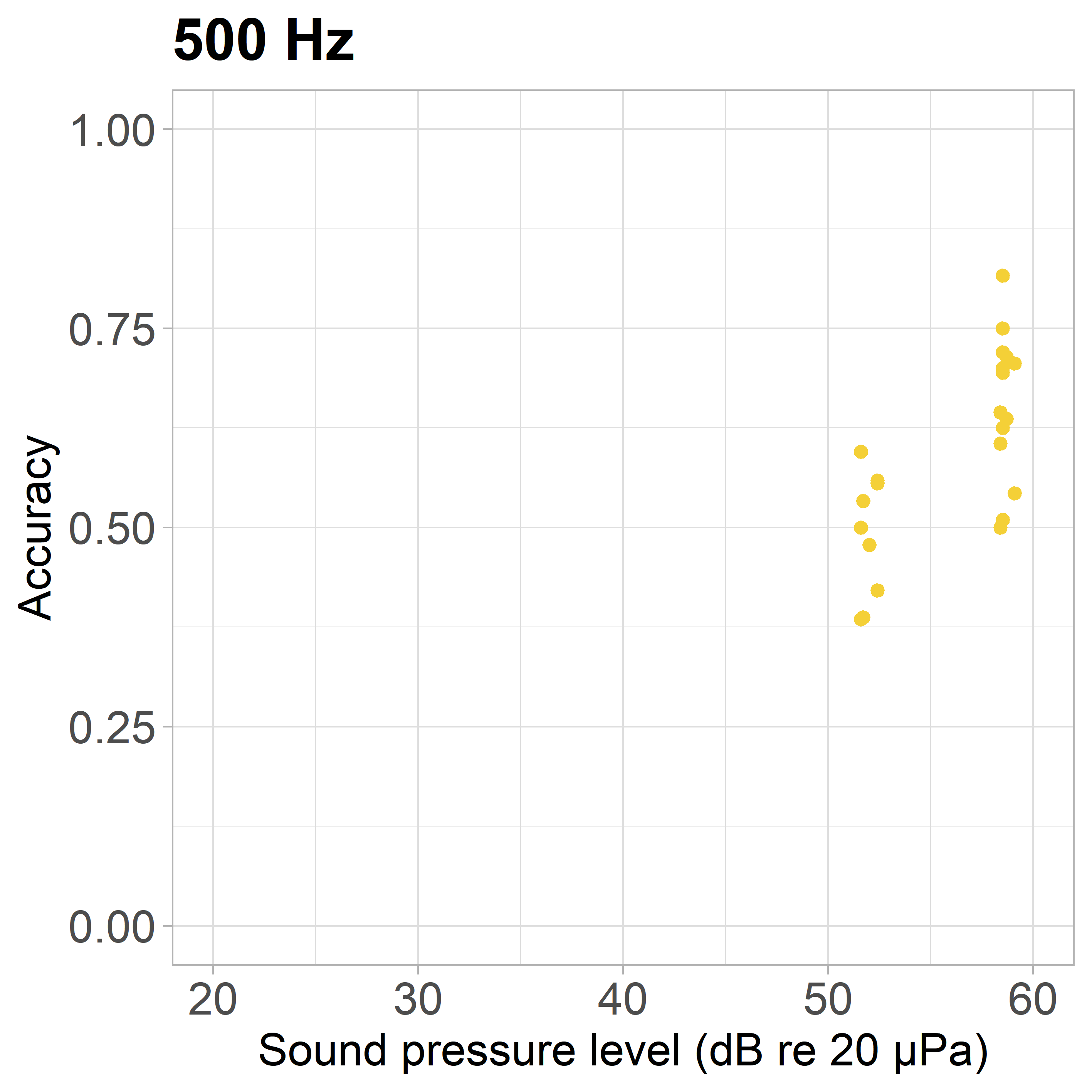


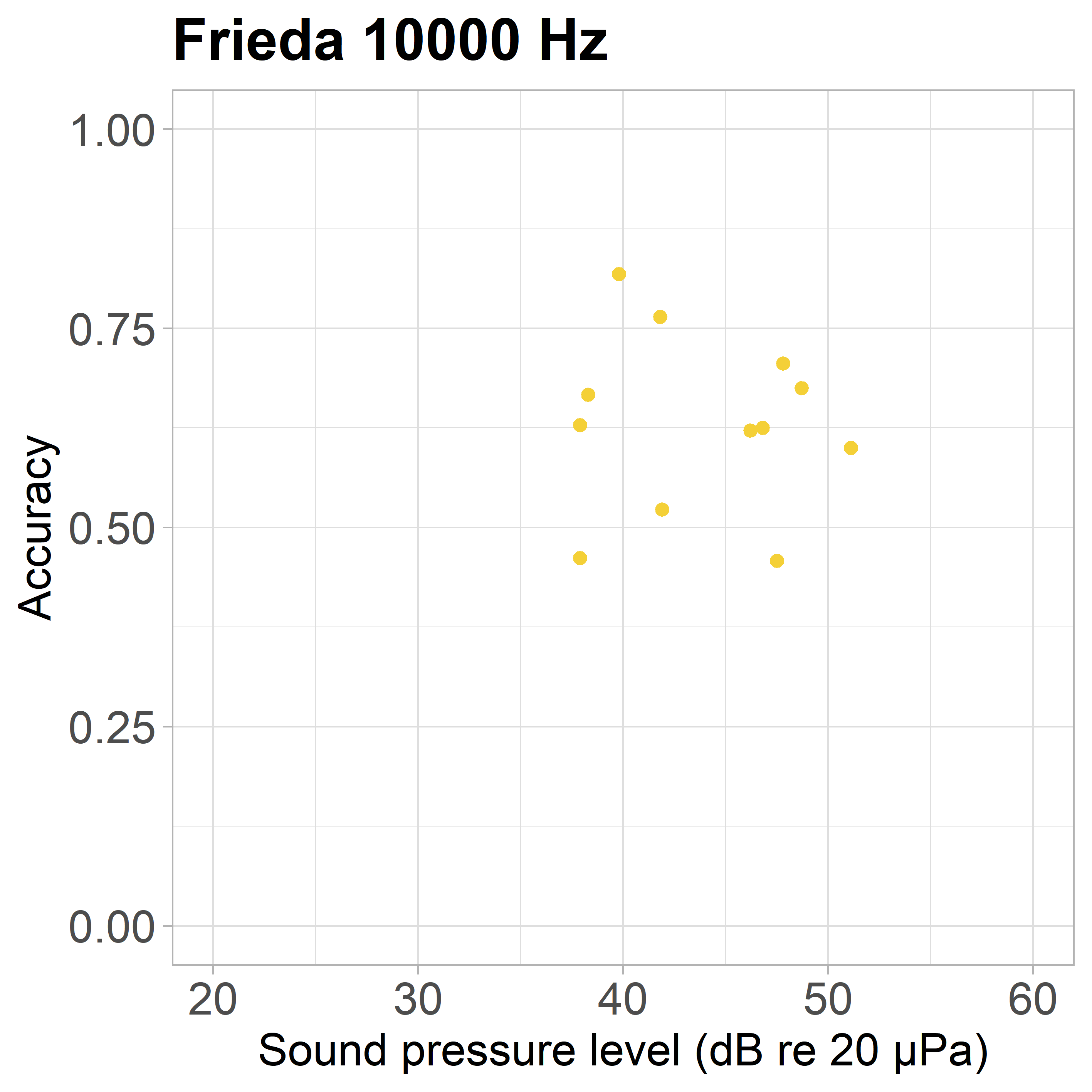

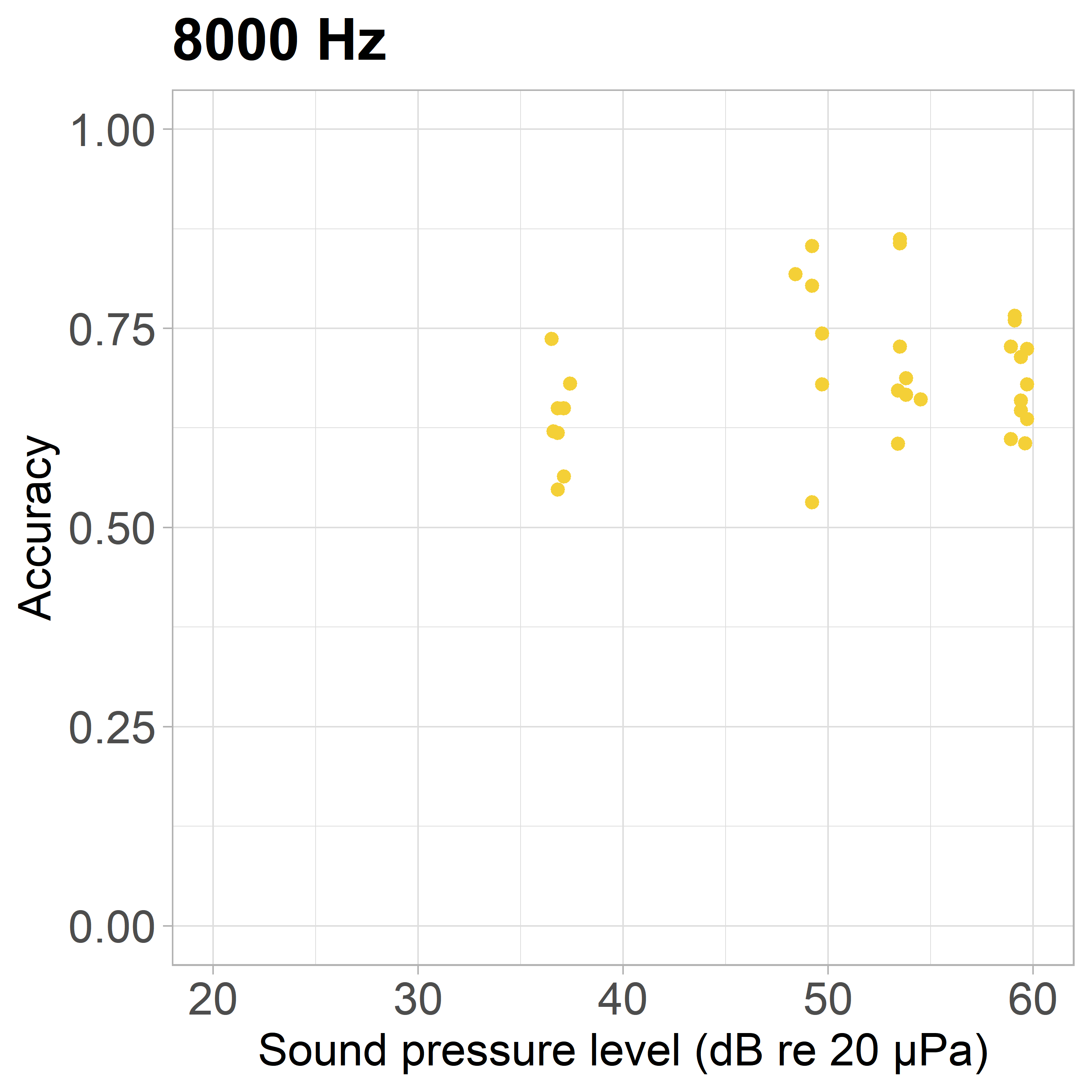


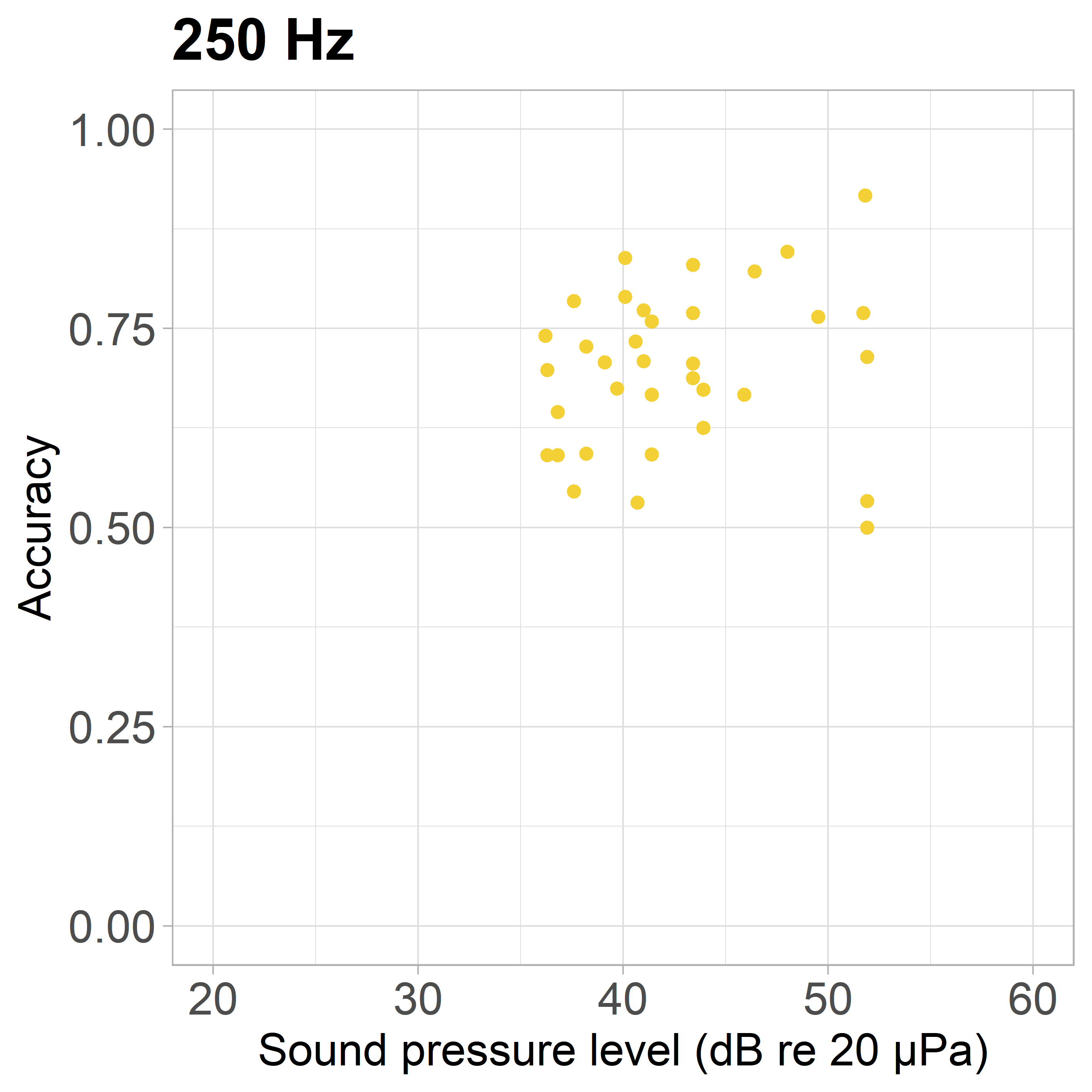


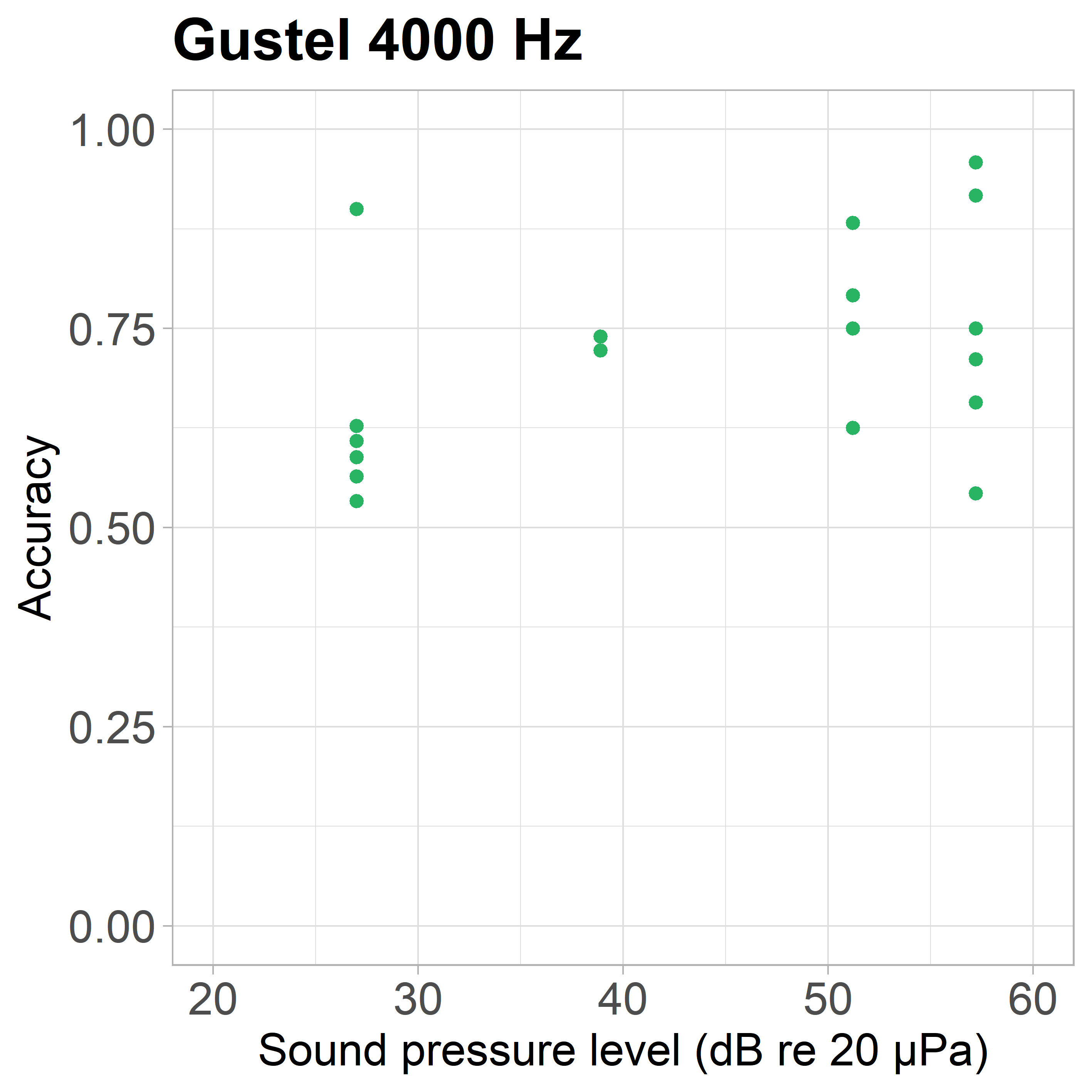

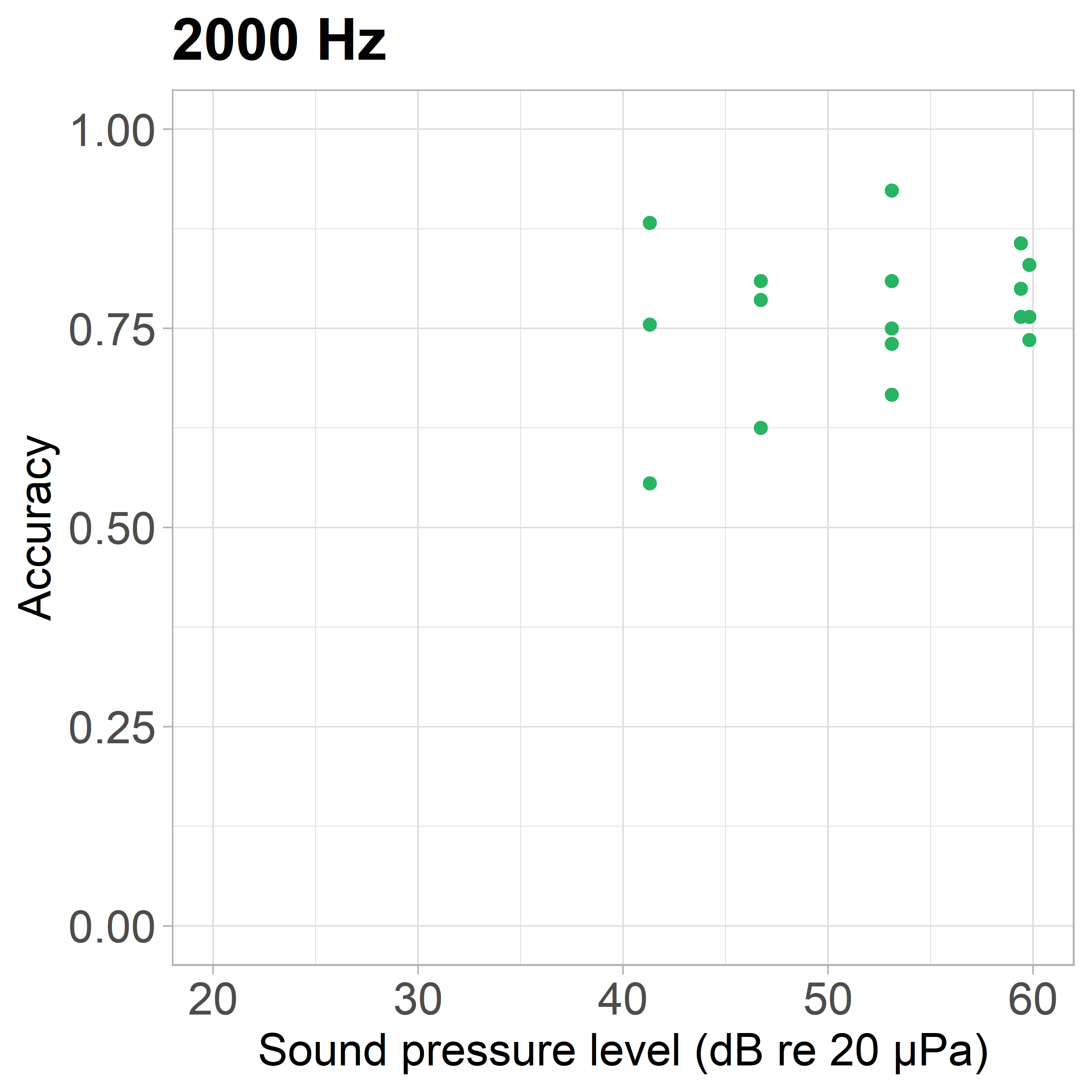

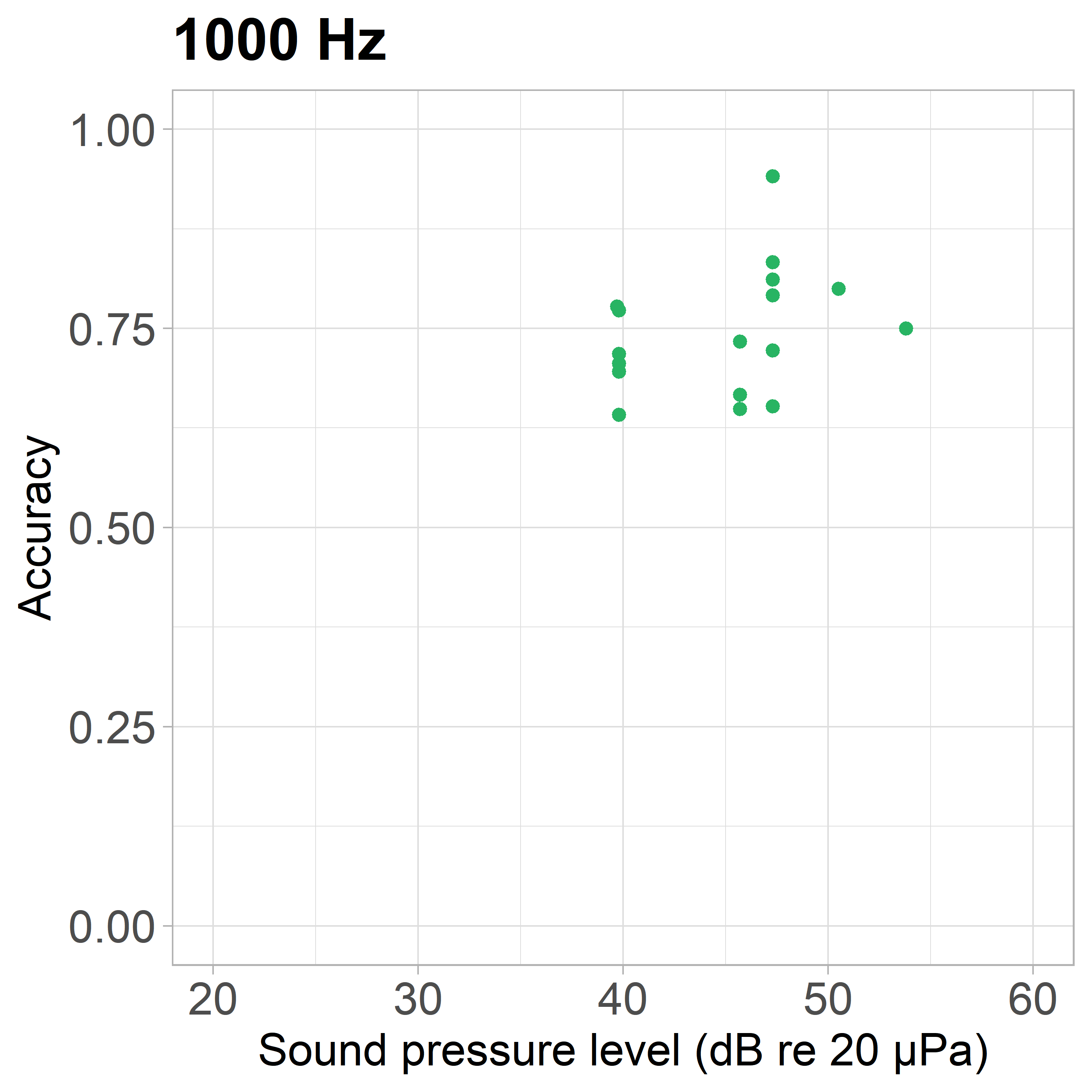

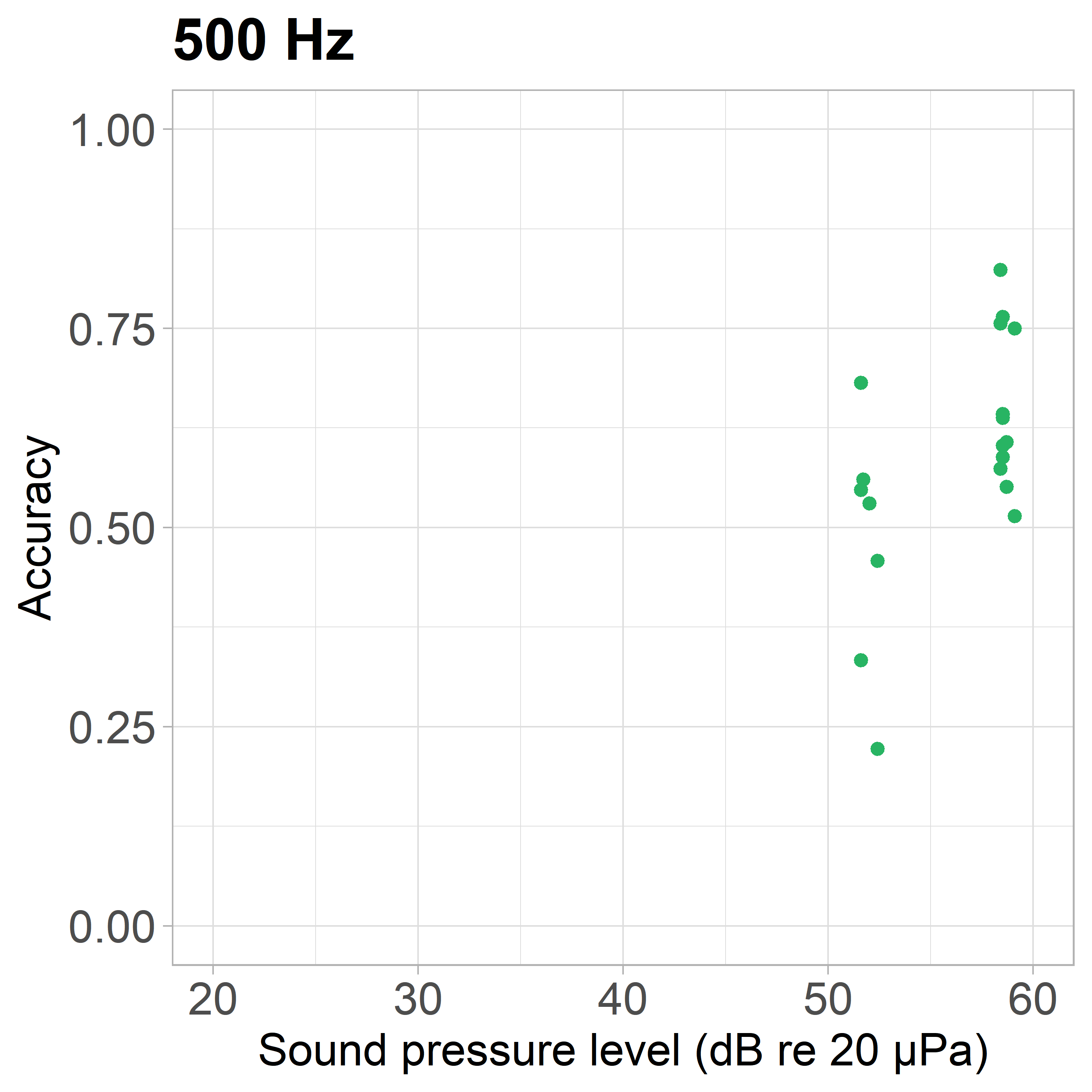


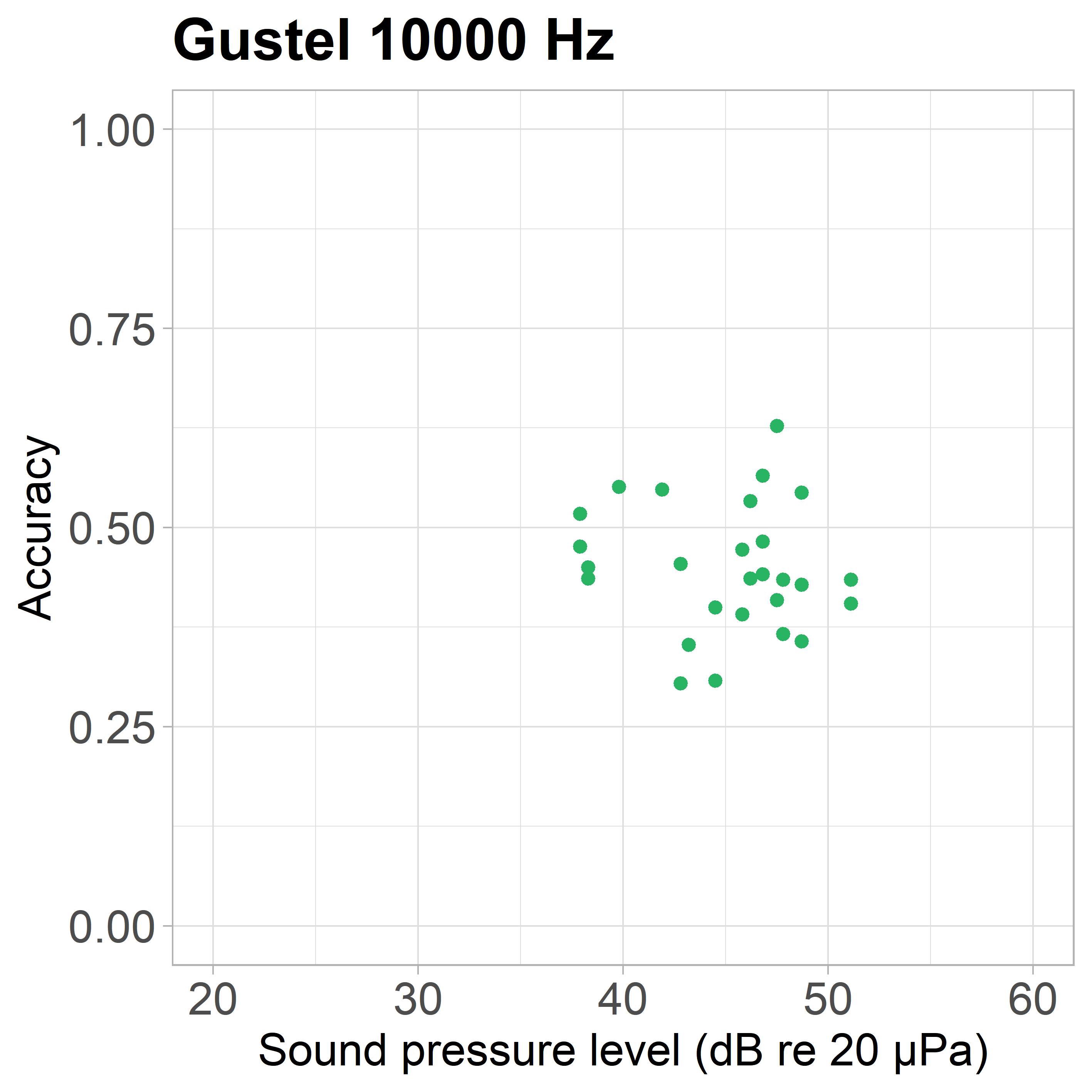

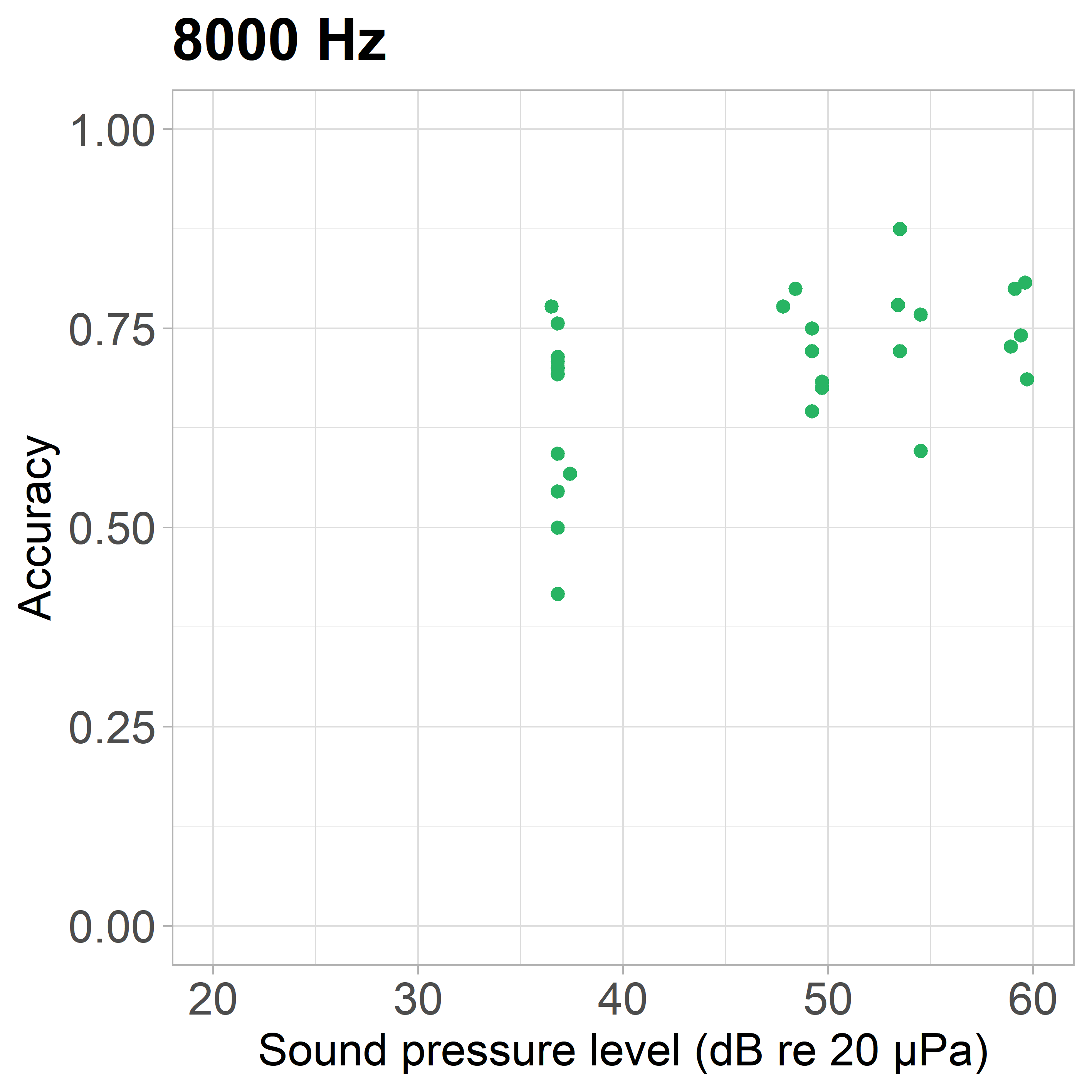


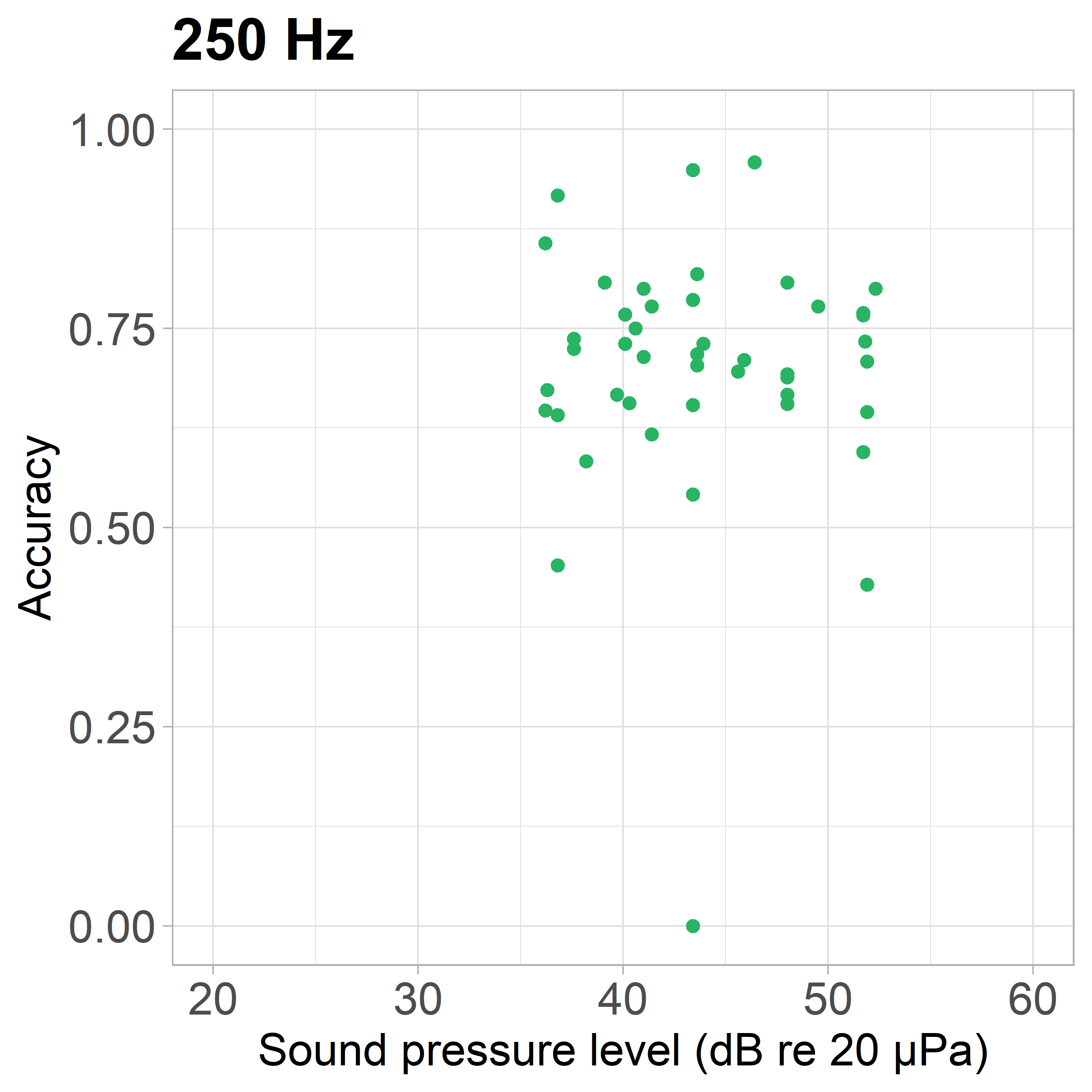


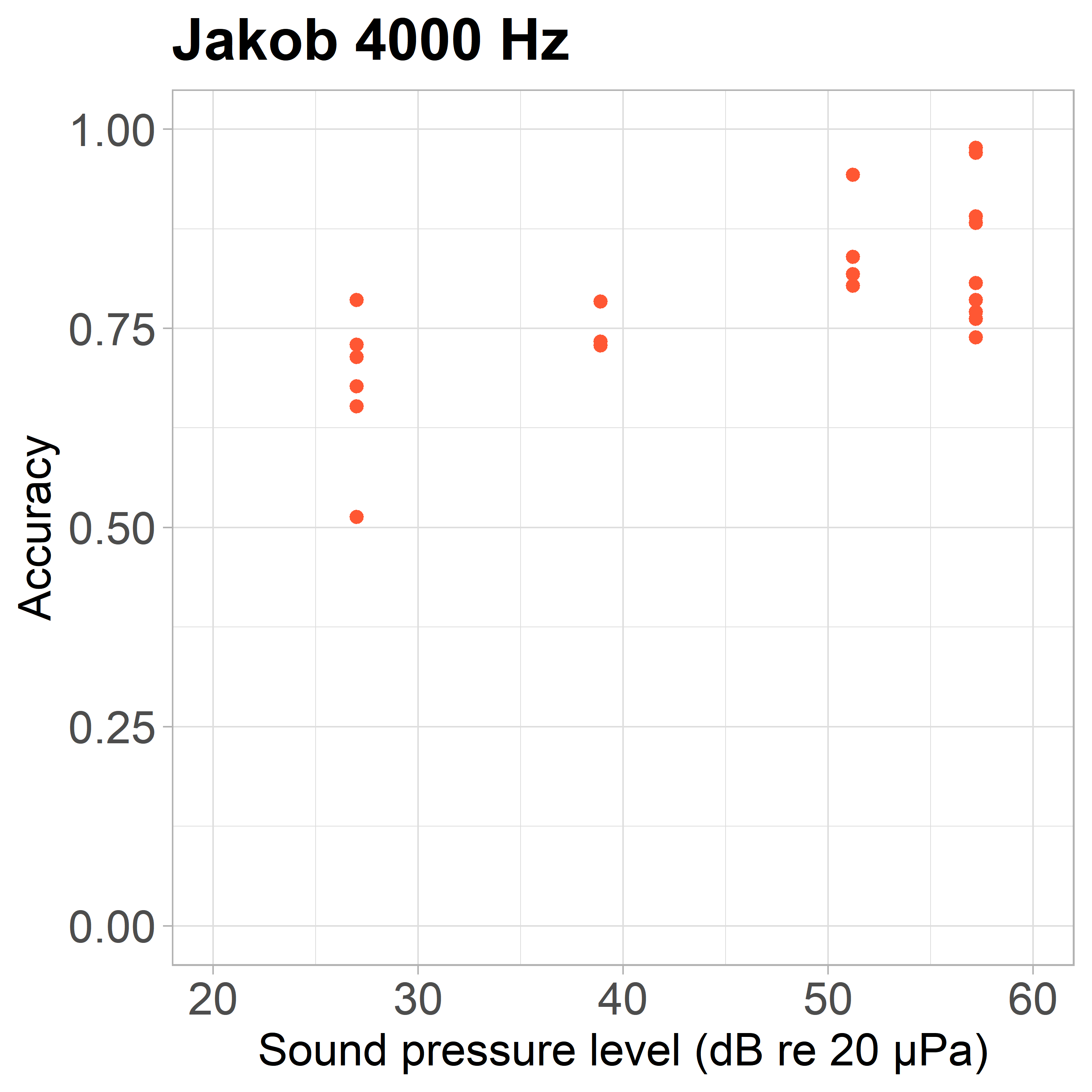

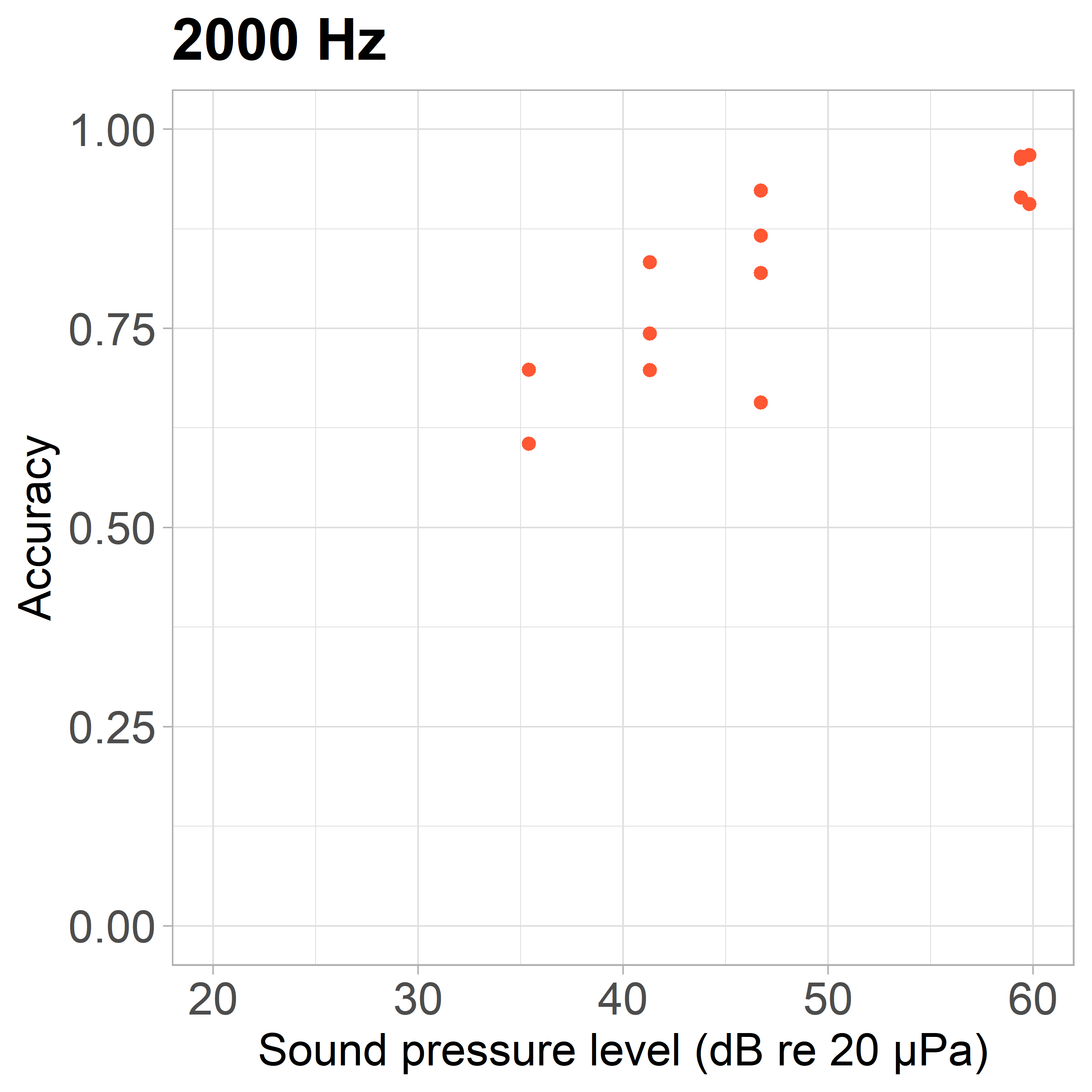

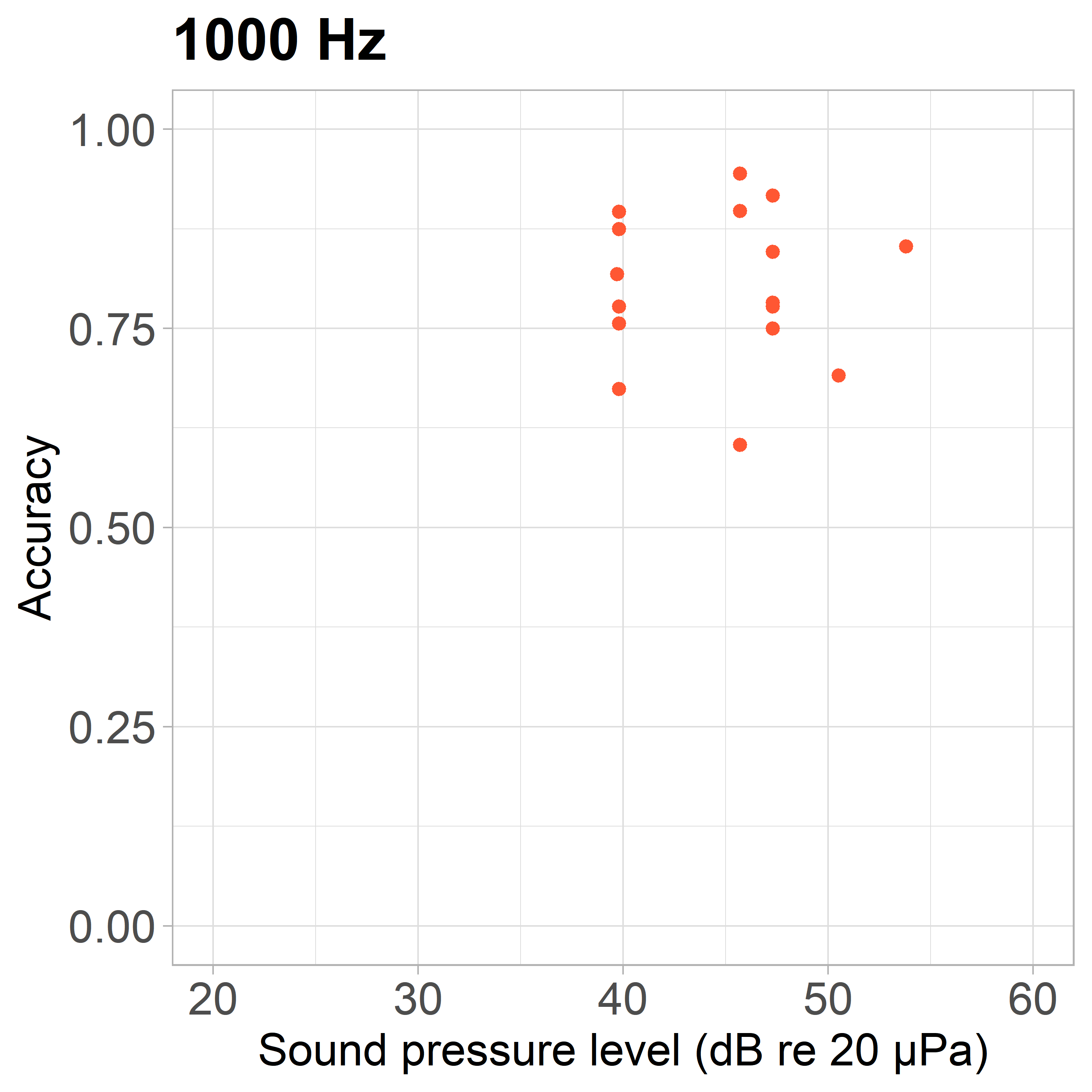

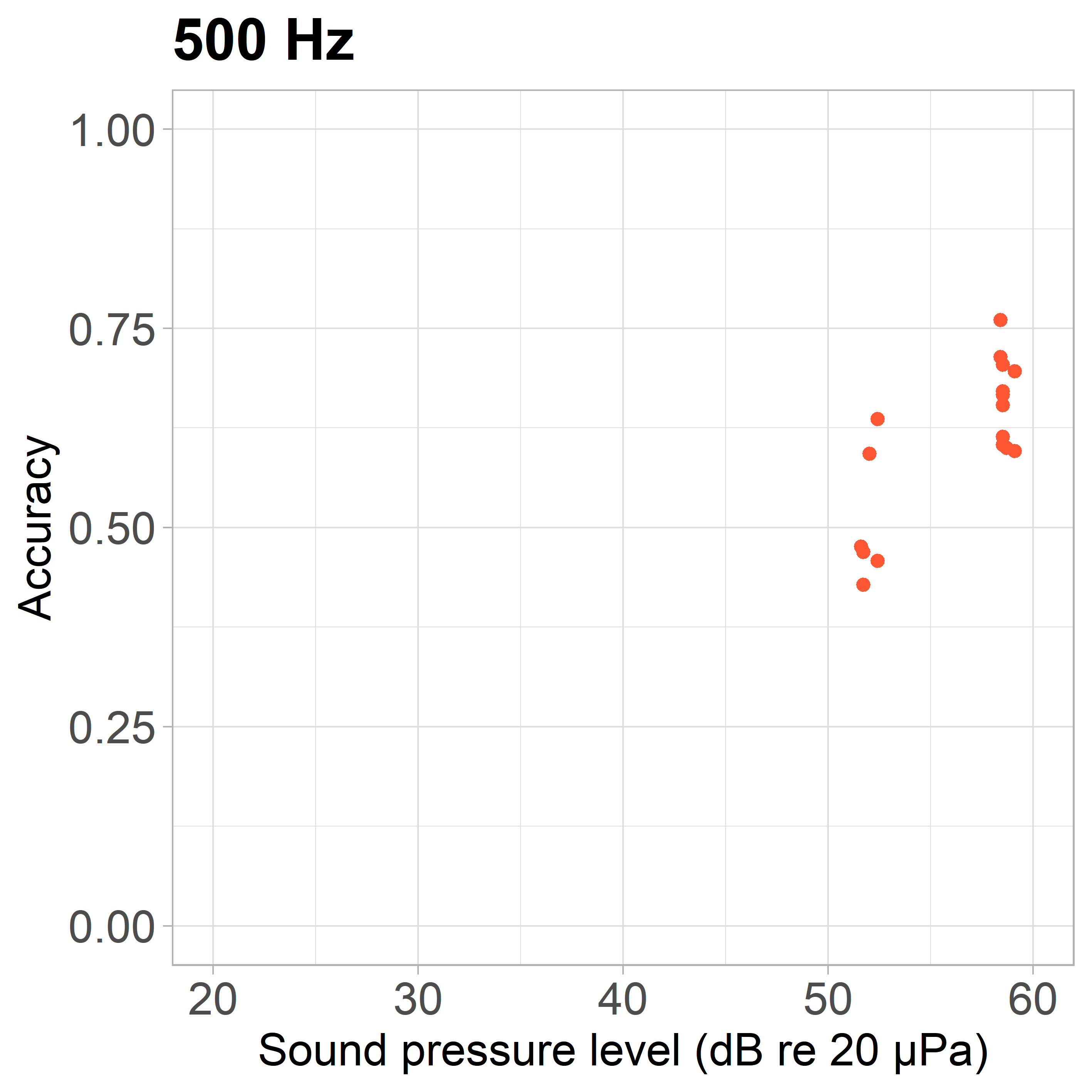


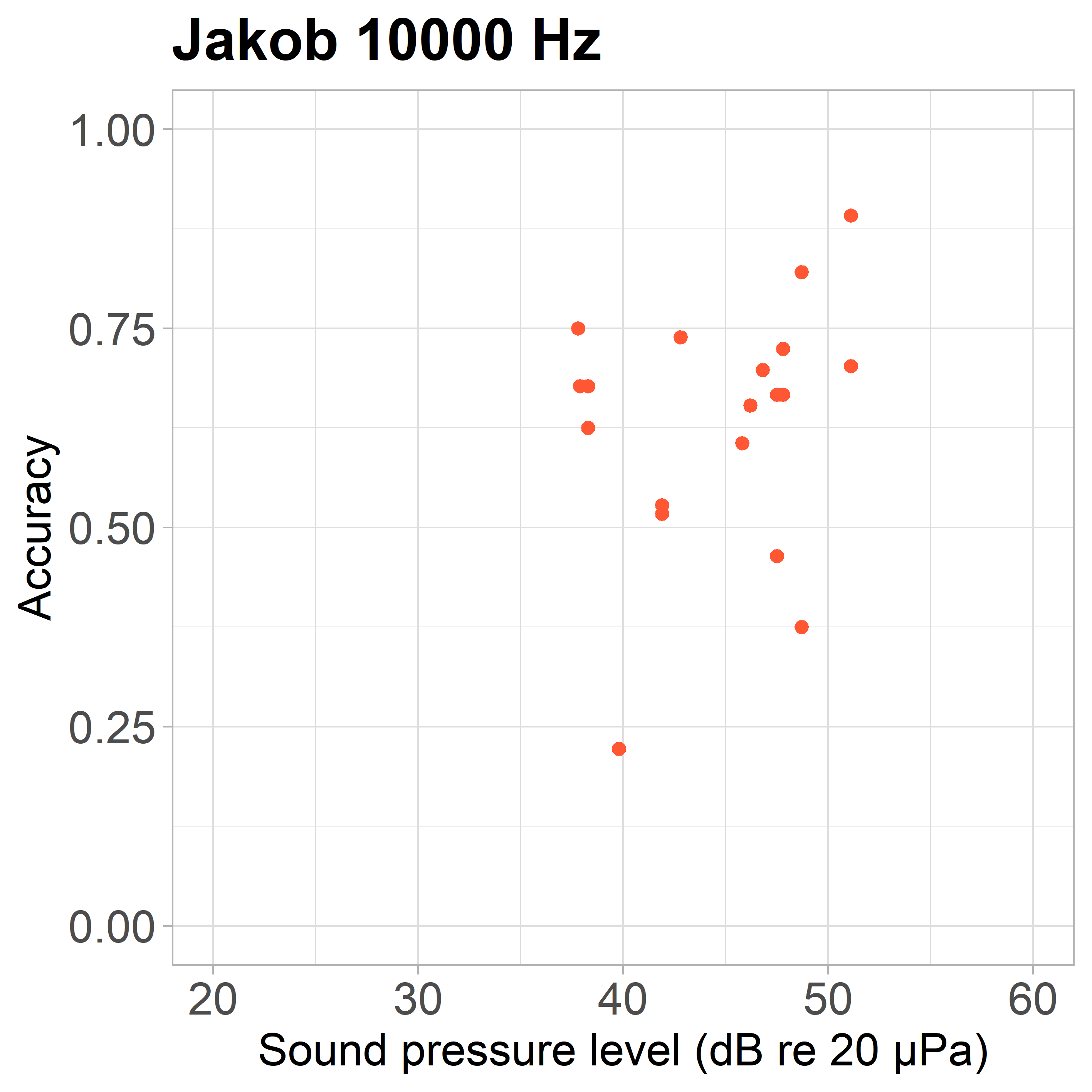

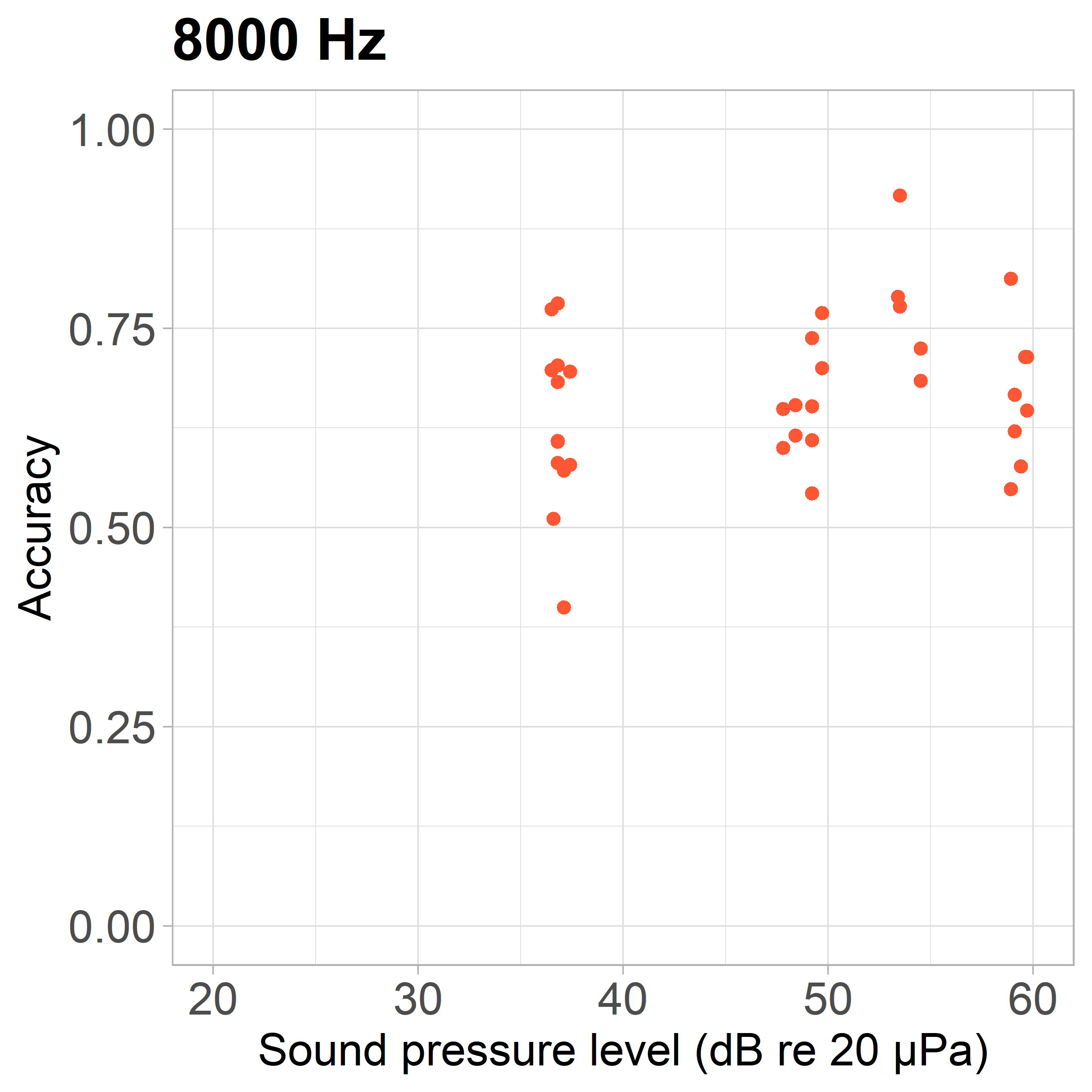


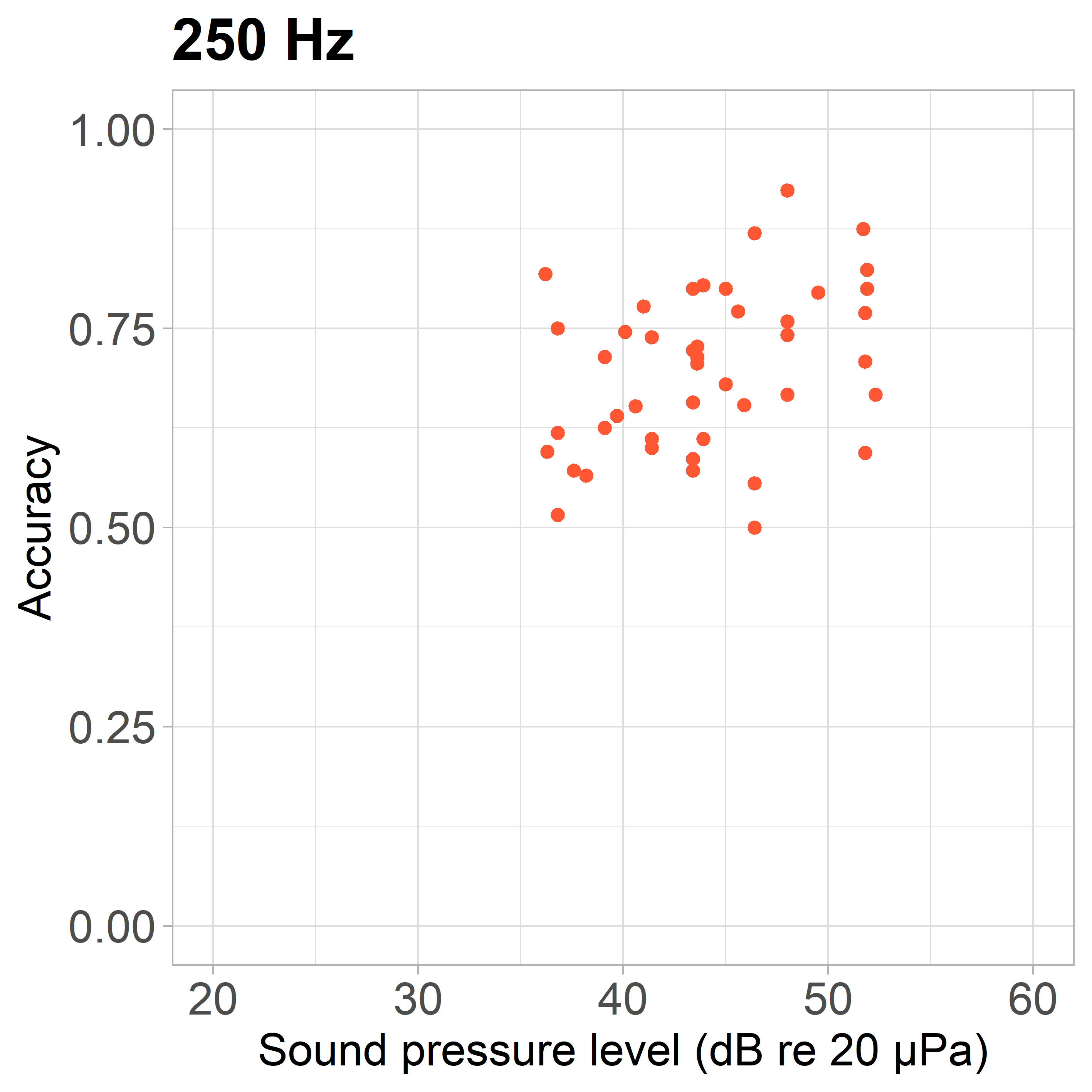


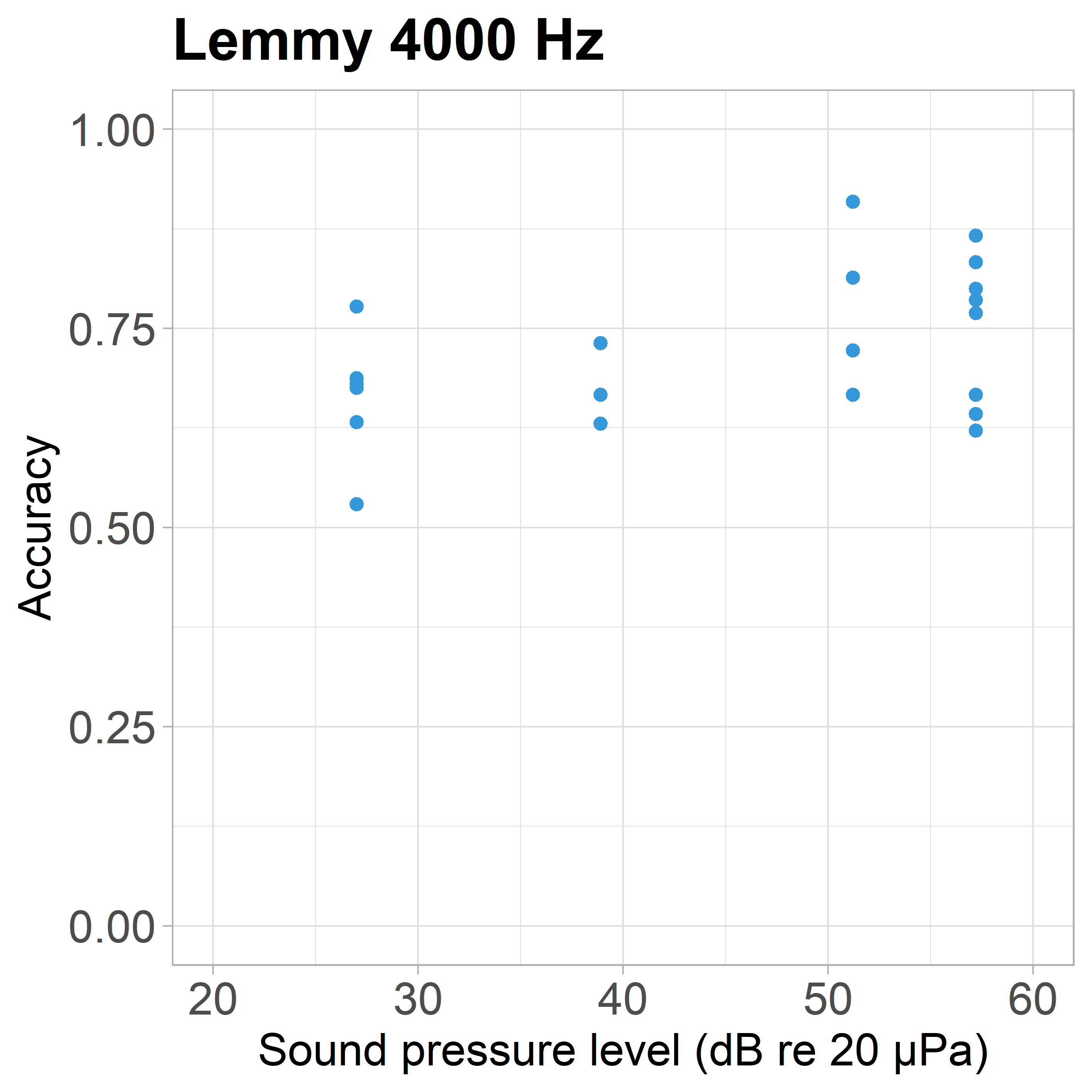

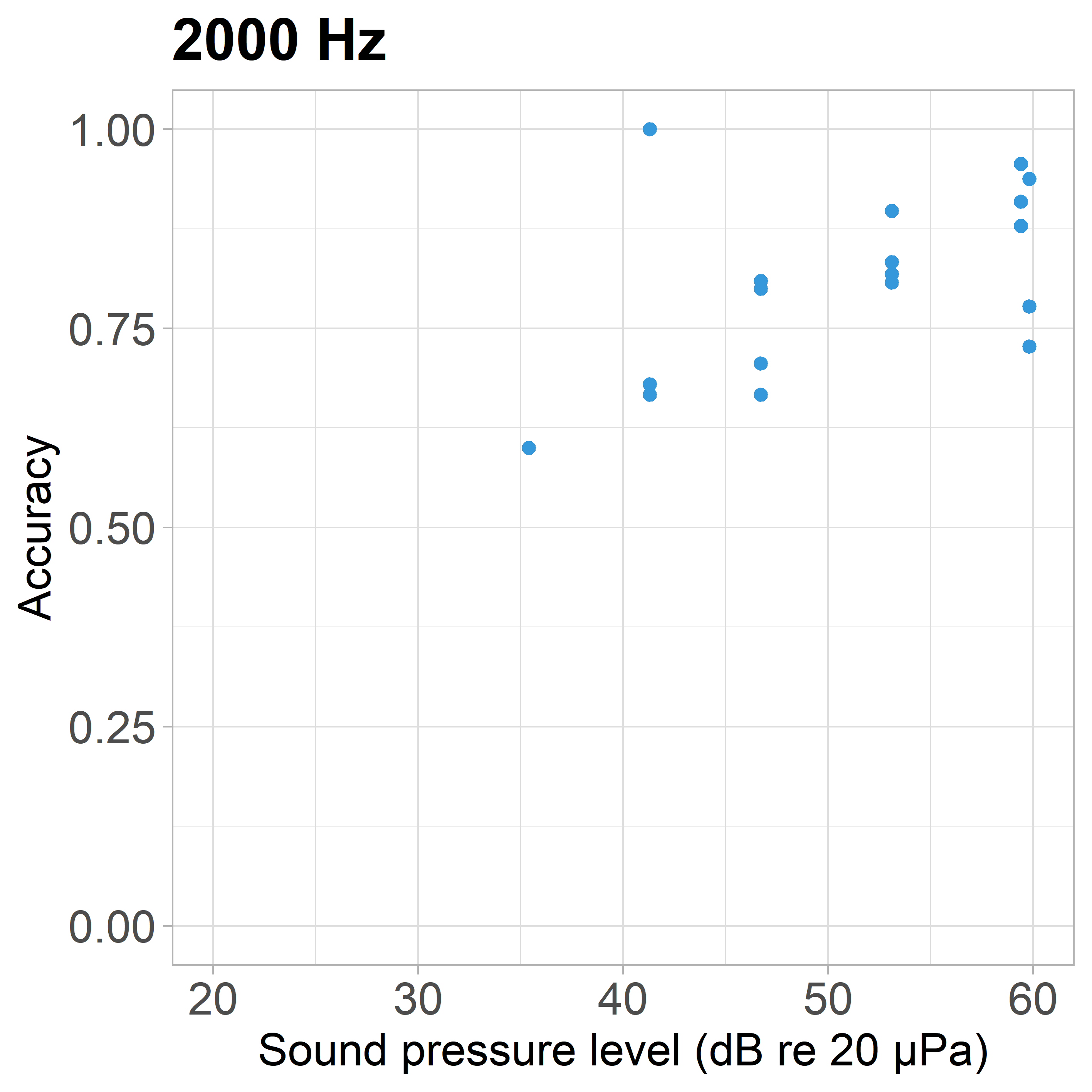

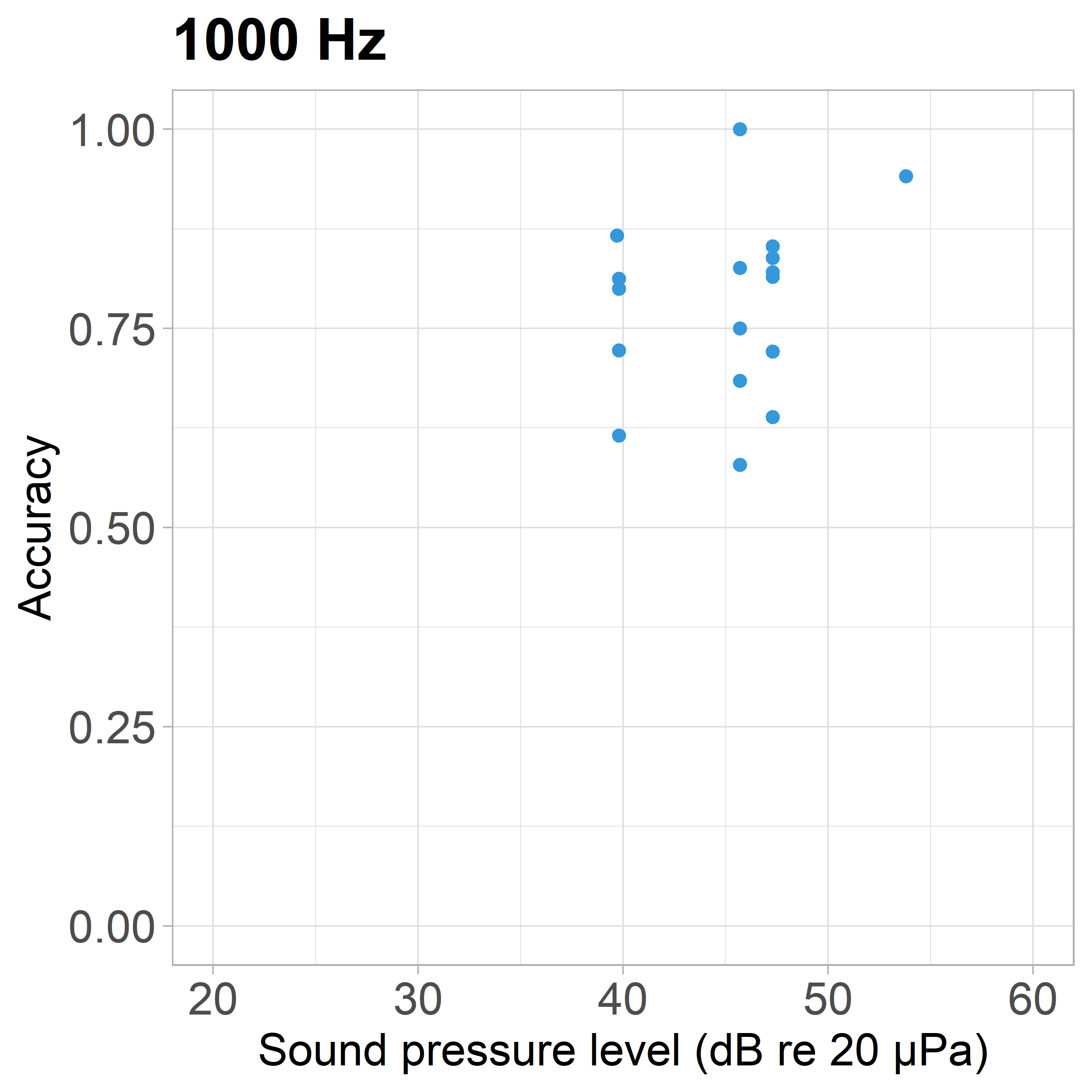

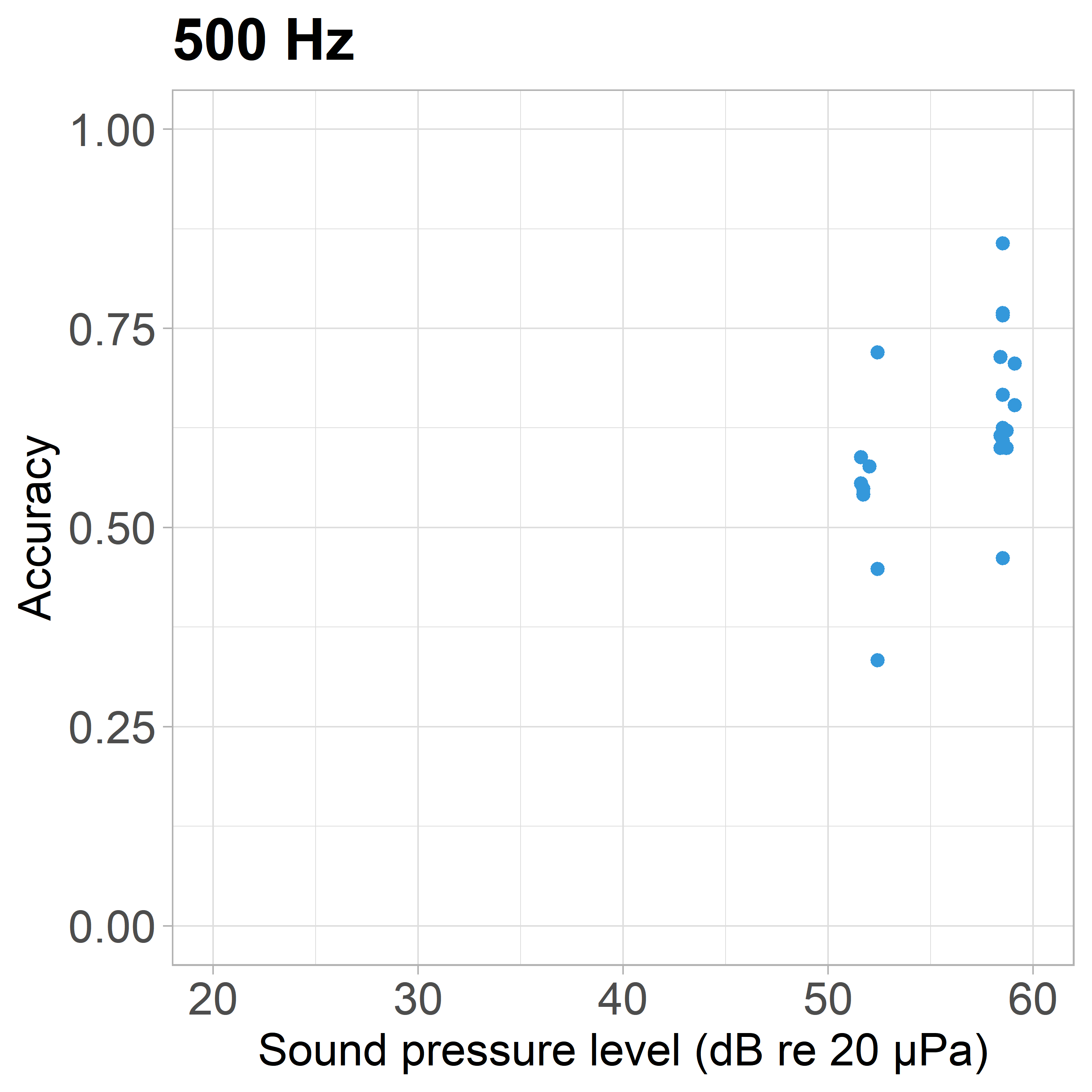


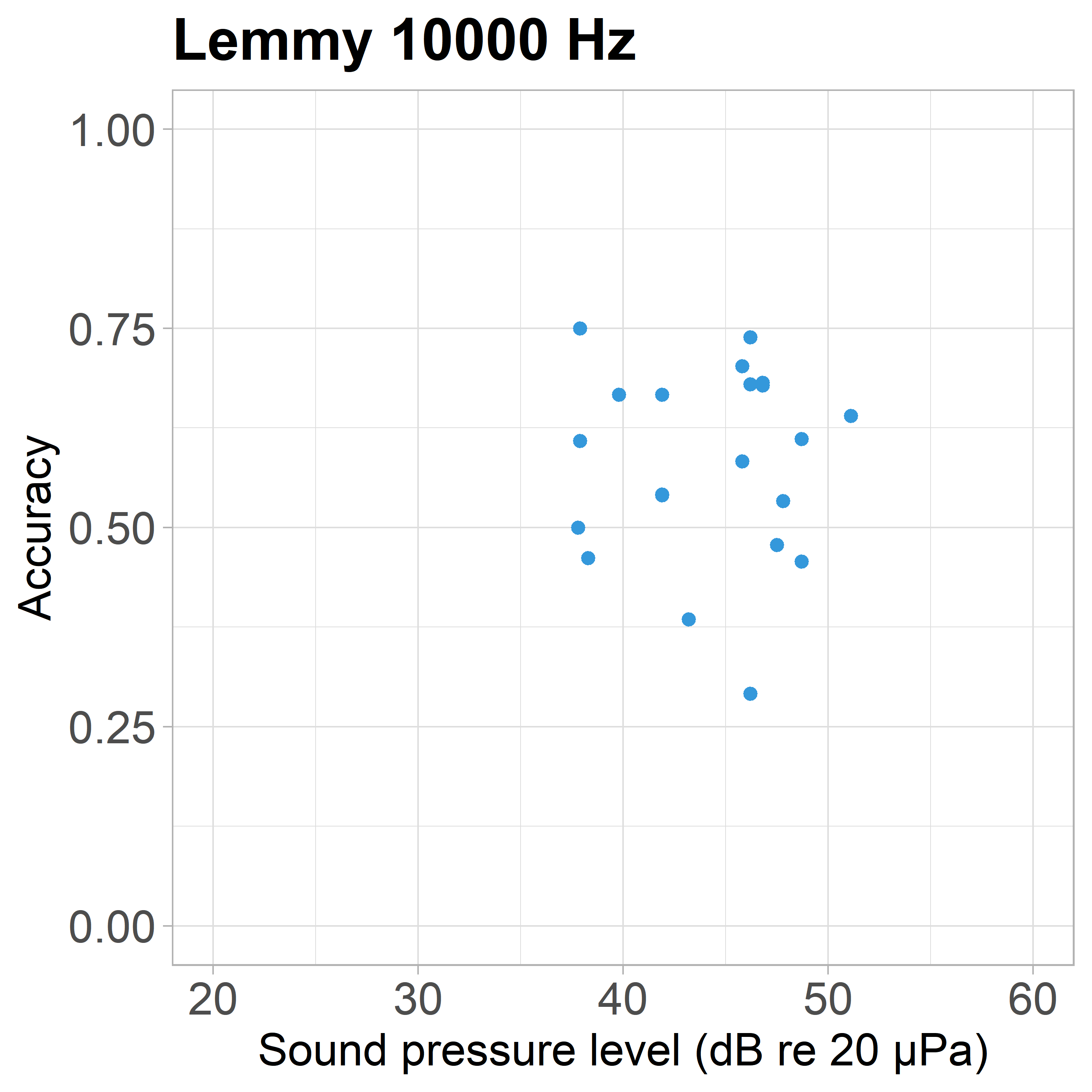

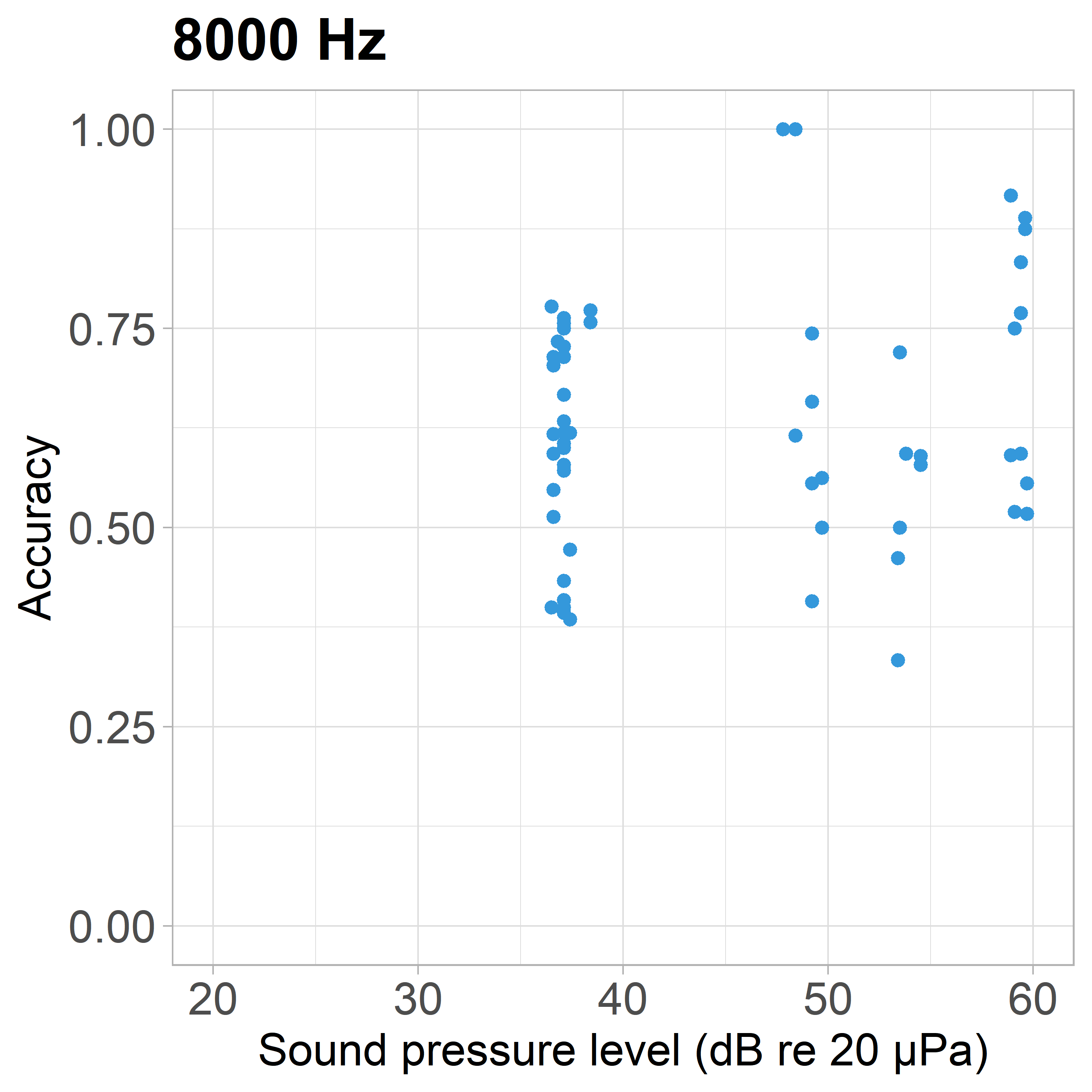


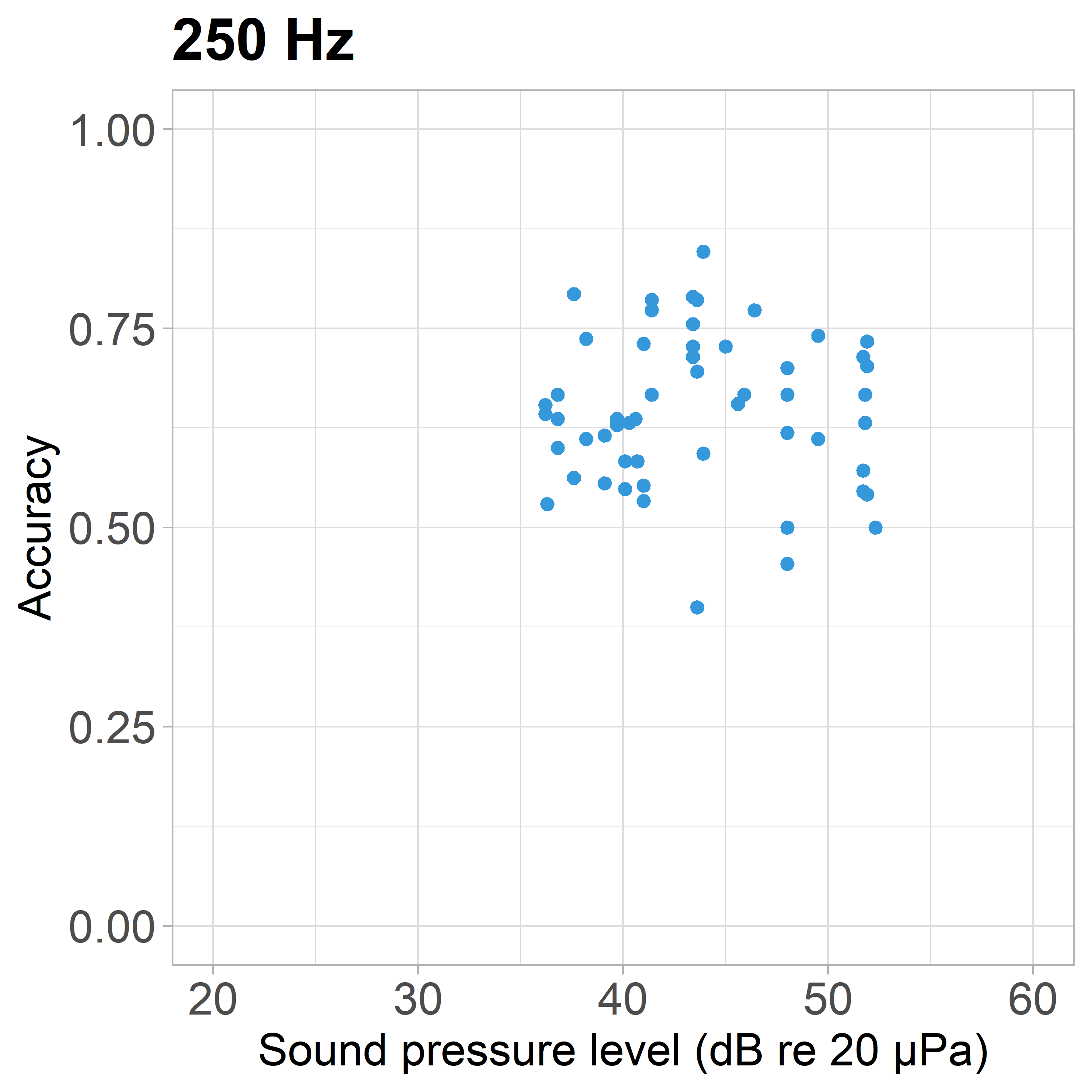


Figure S1: Accuracy at c session type (= (Hit + True negative)/ ((Hit + Miss) + (True negative + False Alarm))) for each of the four penguins (Frieda= yellow, Gustel = green, Jakob = red and Lemmy = blue) at 7 different frequencies and reducing sound pressure levels. Each dot is one session with 20 - 80 trials.

See pdf attachment file *Figure S2_SDT_FA35_20240305_revised.pdf for Fig S2*

Figure S2: The Hit rate, detectability (d’), Fa rate, criterion beta, amount of trials, and amount of sessions as a function of sound pressure level (SPL) in psychophysical trials of the in-air hearing abilities of the four Humboldt penguins. Coloured lines are regression lines used to determine a threshold of the bird’s performance at cumulative normal distribution function probability = 0.5 for each frequency (indicated by a grey dashed horizontal line), hit rate = 50 % (indicated by a red dotted horizontal line), d’ = 1 (indicated by a red dotted horizontal line) and d’ = 1.4 (indicated by a grey dotted horizontal line).


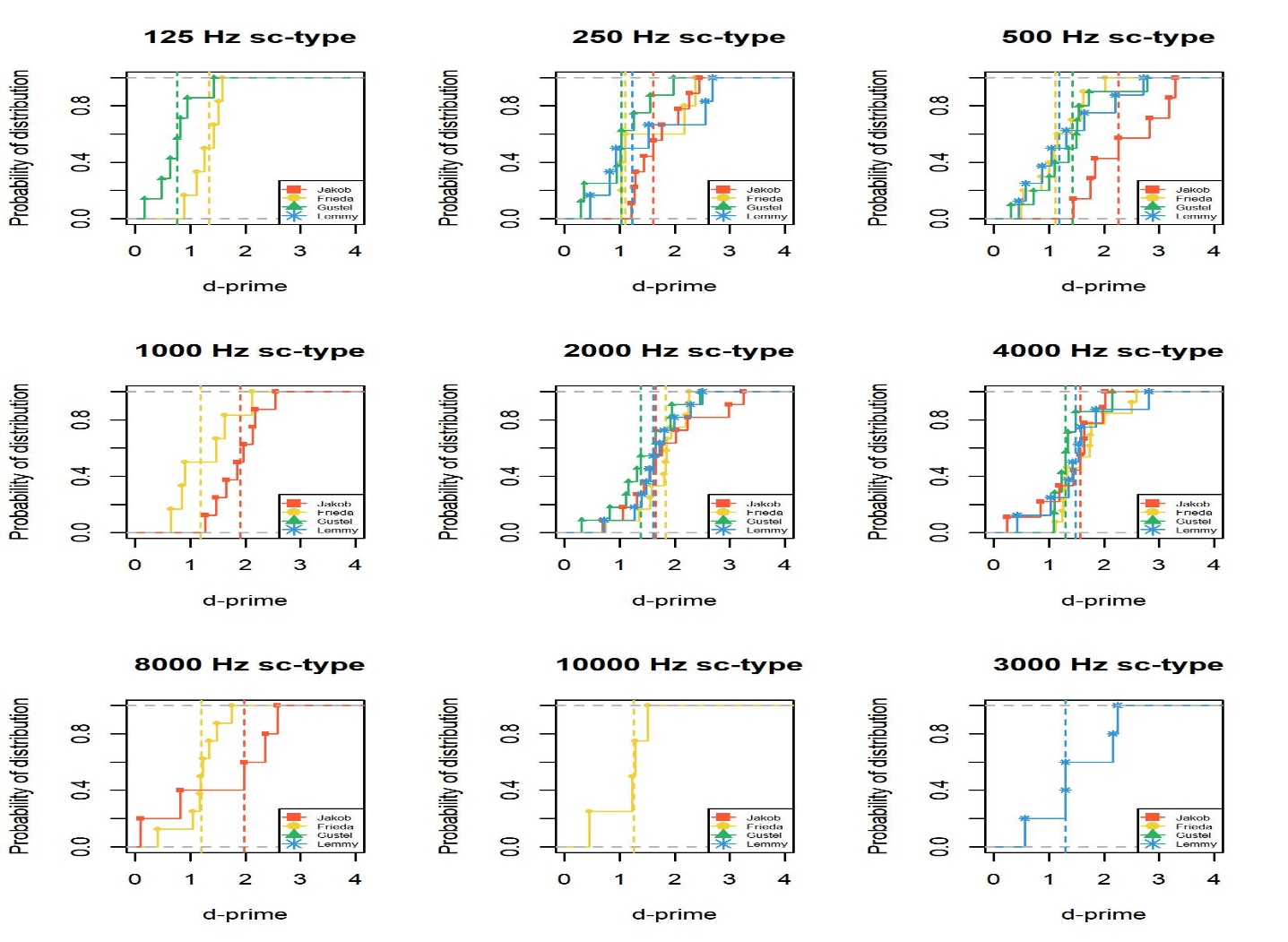


Figure S3: Cumulative normal distribution as a function of d-prime for 35 % FA - rate, for all individuals at each frequency, with the dashed line at the 50 % quantile of probability of distribution for each individual at each frequency. Where the dashed line intercepts the x - axis, the limit for the regression for the threshold determination criterion was set.

Figure S4: In air audiogram of four Humboldt penguins with data point of d-prime values (top = d’prob0.5, bottom = d’1.4) as threshold detection criterion. Jakob = red square, Frieda = yellow dot, Gustel = green triangle, Lemmy = blue star.


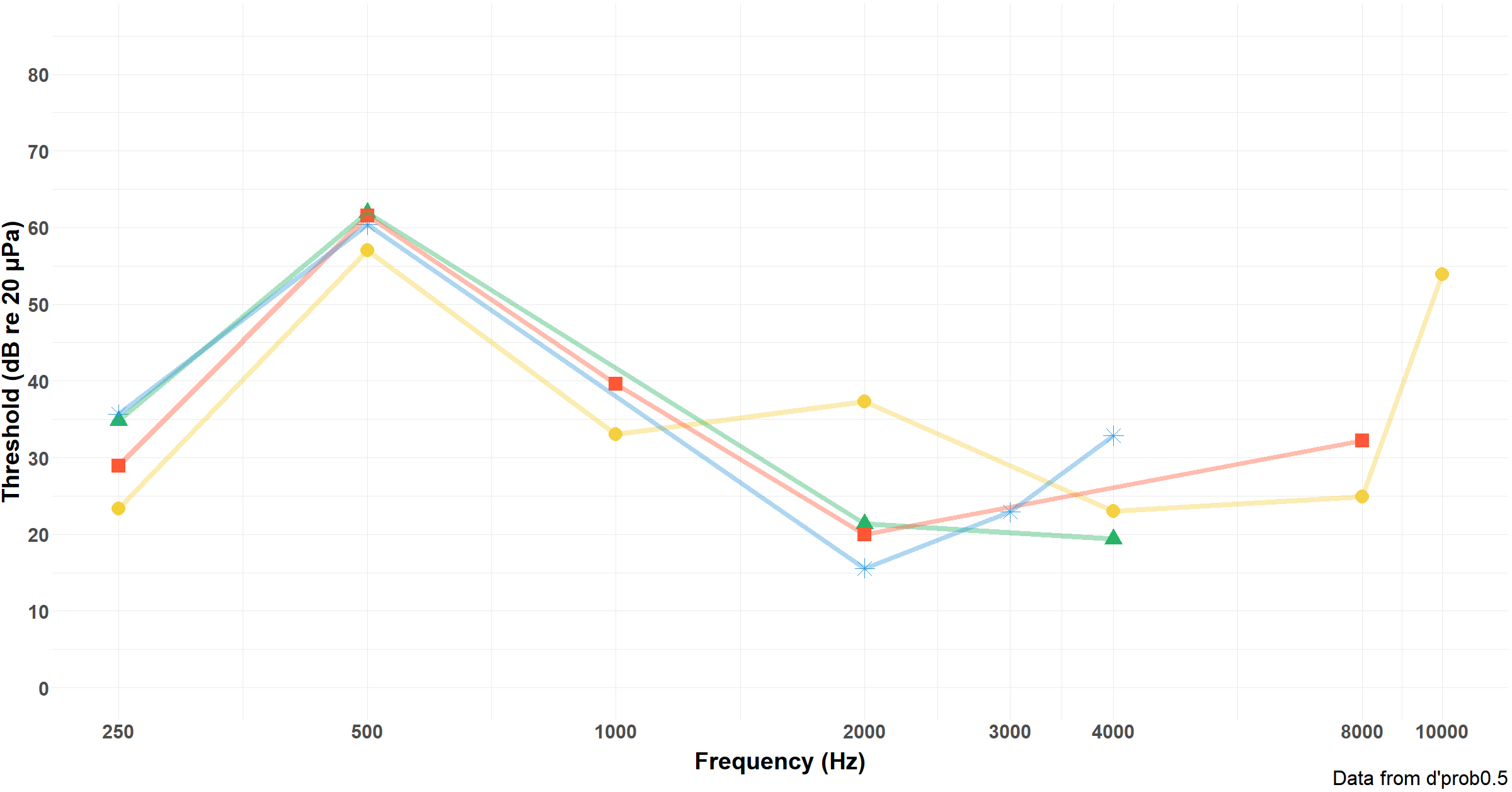

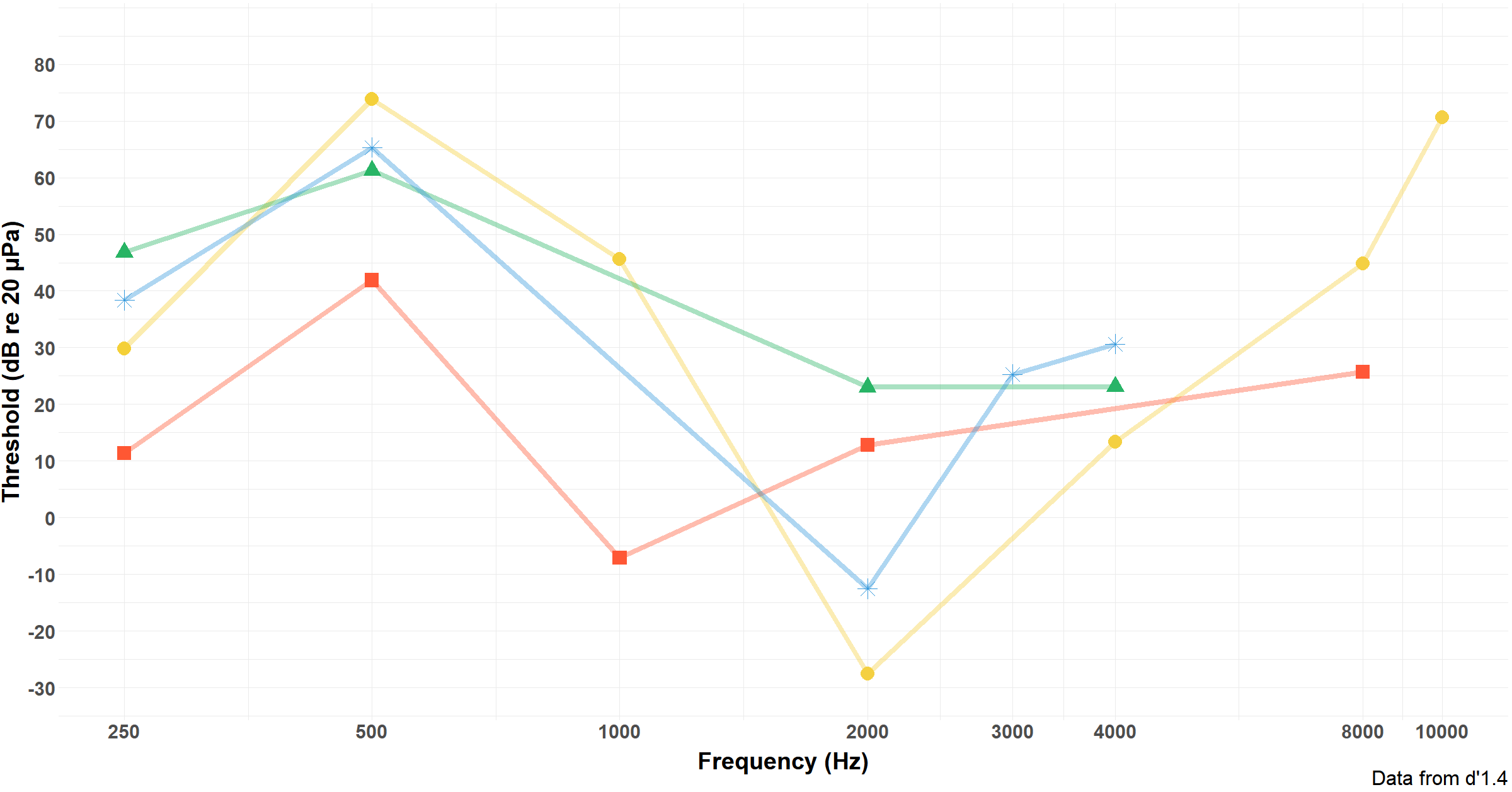


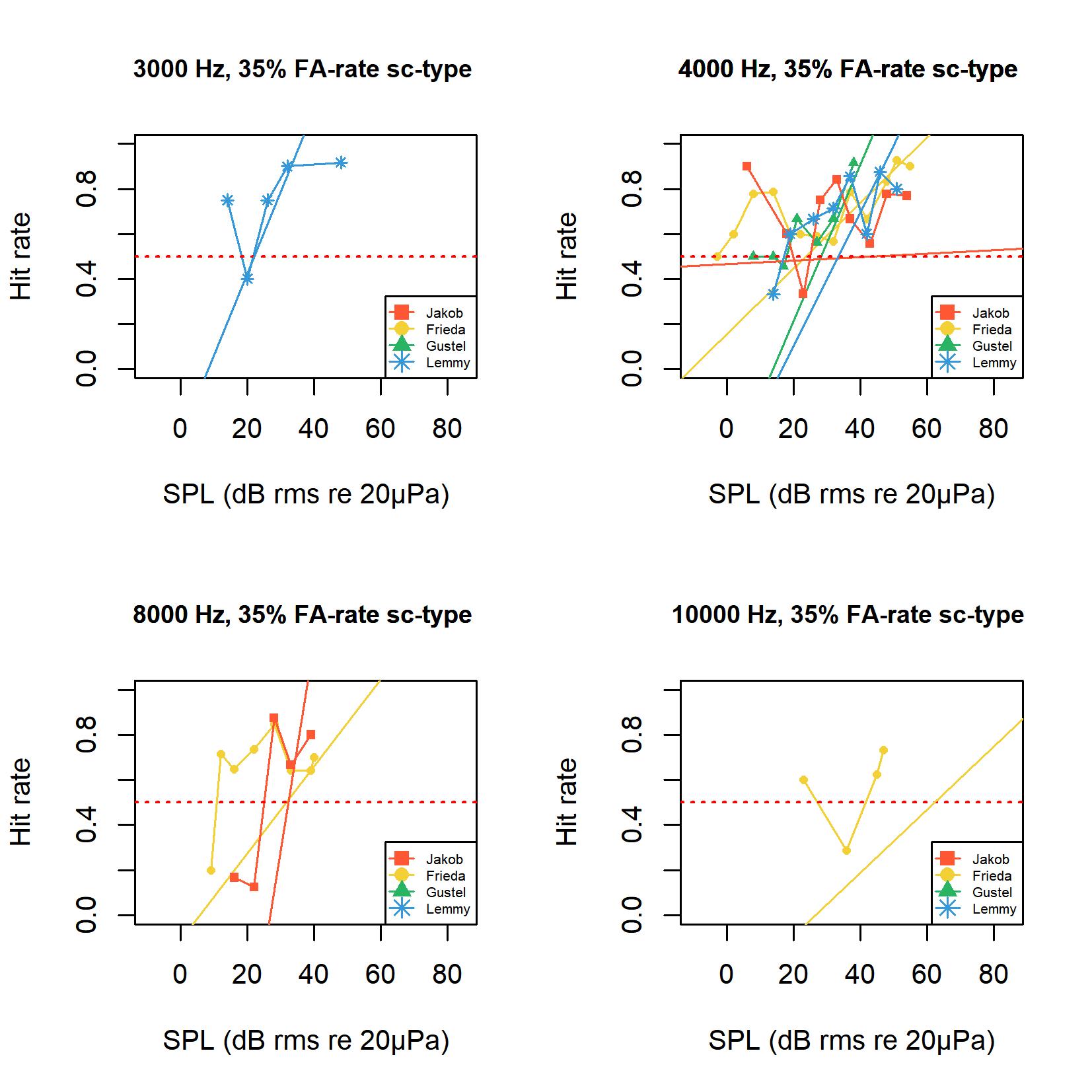


Figure S5: Hit rate as a function of sound pressure level (SPL) shown per frequency for one to four animals after cleaning the Data. Psychometric functions generated by probit fitting for each tested frequency and individual.
